# Leveraging Large Language Models for High-Quality Lay Summaries: Efficacy of ChatGPT-4 with Custom Prompts in a Consecutive Series of Prostate Cancer Manuscripts

**DOI:** 10.3390/curroncol32020102

**Published:** 2025-02-11

**Authors:** Emily Rinderknecht, Anna Schmelzer, Anton Kravchuk, Christopher Goßler, Johannes Breyer, Christian Gilfrich, Maximilian Burger, Simon Engelmann, Veronika Saberi, Clemens Kirschner, Dominik von Winning, Roman Mayr, Christian Wülfing, Hendrik Borgmann, Stephan Buse, Maximilian Haas, Matthias May

**Affiliations:** 1 Department of Urology, St. Josef Medical Center, University of Regensburg, 93053 Regensburg, Germany; 2 Working Group on Artificial Intelligence and Digitalization of the German Society of Urology; 3 Department of Urology, St. Elisabeth Hospital Straubing, 94315 Straubing, Germany; 4 Department of Urology, Asklepios Klinik Altona, 22763 Hamburg, Germany; 5 Department of Urology, Brandenburg Medical School Theodor Fontane (MHB), 14770 Brandenburg an der Havel, Germany; 6 Department of Urology, Alfried Krupp Krankenhaus, 45131 Essen, Germany

**Keywords:** patient communication, artificial intelligence in healthcare, language model applications, plain language summaries, lay abstracts, prompt design, medical application, text generation, scientific literacy, readability metrics

## Abstract

Clear and accessible lay summaries are essential for enhancing the public understanding of scientific knowledge. This study aimed to evaluate whether ChatGPT-4 can generate high-quality lay summaries that are both accurate and comprehensible for prostate cancer research in *Current Oncology*. To achieve this, it systematically assessed ChatGPT-4’s ability to summarize 80 prostate cancer articles published in the journal between July 2022 and June 2024 using two distinct prompt designs: a basic “simple” prompt and an enhanced “extended” prompt. Readability was assessed using established metrics, including the Flesch–Kincaid Reading Ease (FKRE), while content quality was evaluated with a 5-point Likert scale for alignment with source material. The extended prompt demonstrated significantly higher readability (median FKRE: 40.9 vs. 29.1, *p* < 0.001), better alignment with quality thresholds (86.2% vs. 47.5%, *p* < 0.001), and reduced the required reading level, making content more accessible. Both prompt designs produced content with high comprehensiveness (median Likert score: 5). This study highlights the critical role of tailored prompt engineering in optimizing large language models (LLMs) for medical communication. Limitations include the exclusive focus on prostate cancer, the use of predefined prompts without iterative refinement, and the absence of a direct comparison with human-crafted summaries. These findings underscore the transformative potential of LLMs like ChatGPT-4 to streamline the creation of lay summaries, reduce researchers’ workload, and enhance public engagement. Future research should explore prompt variability, incorporate patient feedback, and extend applications across broader medical domains.

## 1. Introduction

Lay summaries aim to make the findings of scientific medical literature transparent and accessible to broader audiences, including study participants, patients, stakeholders, and the general public. Within the European Union, the integration of lay summaries is mandatory for clinical trials under the European Union Clinical Trials Regulation (EU CTR) 536/2014 [1,2]. These summaries are intended to convey complex scientific content in plain language, ensuring clarity and comprehension for readers with varying levels of scientific literacy [1].

The importance of lay summaries has grown significantly, with many renowned publishers, such as Elsevier’s *European Urology* family of journals, requiring them [3]. Similarly, the Multidisciplinary Digital Publishing Institute (MDPI) mandates lay summaries for patient-oriented research in select journals, including *Cancers* [4]. However, despite its prominence as a high-impact MDPI journal, *Current Oncology* does not currently require lay summaries from its authors, highlighting a gap in accessibility for non-specialist audiences. Given its clinical relevance and broad readership, evaluating strategies to enhance the accessibility of its published research is essential.

Effective lay summaries must consider the target audience and translate intricate scientific concepts into accurate yet comprehensible narratives [5]. Guidelines such as the Good Lay Summary Practice, published in 2021 by the European Union, emphasize that lay summaries should be understandable to individuals with a reading age of approximately 12 years [5,6]. Despite these efforts, research consistently highlights that medical documents intended for laypersons often fail to meet adequate readability standards [6,7,8,9,10]. The challenge lies in bridging the gap between scientific accuracy and plain language, a task many professionals struggle to achieve [11].

Large language models (LLMs), such as ChatGPT, offer a promising solution by automating the generation of lay summaries through advanced natural language processing capabilities [12]. Preliminary studies, including those by Eppler et al., suggest that ChatGPT-3.5 can generate moderately accurate summaries, though expert verification remains essential [13]. Recent work by Shyr et al. demonstrated the potential of ChatGPT-4 in creating accessible and accurate lay summaries, emphasizing its scalability and efficiency when integrated into platforms like ResearchMatch [14]. However, these studies noted the critical importance of prompt design, as it significantly influences the quality and readability of generated summaries [13,14].

Building upon these insights, the BioLaySumm 2024 Shared Task—an international benchmarking challenge on lay summarization models, hosted at the BioNLP Workshop of the Association for Computational Linguistics in 2024—has further demonstrated the growing role of LLMs in biomedical lay summarization. The competition revealed a marked shift towards generative AI approaches. The results underscored that LLM-generated summaries can achieve high levels of relevance and factual consistency [15]. However, many participating teams opted for hybrid approaches, combining different computational methods with the use of generative AI to enhance readability and accuracy [15].

Readability metrics, such as the Flesch–Kincaid Reading Ease (FKRE), Gunning Fog Score (GFS), and Smog Index (SI), offer standardized methods for evaluating text accessibility. These metrics account for sentence complexity, word structure, and syllable count to determine the reading level and ease of comprehension. By leveraging these tools, this study seeks to systematically evaluate ChatGPT-4’s performance in generating lay summaries, focusing on prostate cancer articles published in *Current Oncology* between July 2022 and June 2024. Specifically, this research study aims to (i) assess the readability and factual consistency of ChatGPT-4-generated lay summaries, (ii) investigate how variations in prompt design impact summary quality, and (iii) identify and address existing limitations in the automatic generation of lay summaries. Through this analysis, we seek to determine whether ChatGPT-4 can produce lay summaries that meet established readability standards and correctly communicate key findings to non-expert audiences. As a leading journal in oncology, *Current Oncology* provides an ideal framework to evaluate the applicability of LLMs in enhancing the accessibility of biomedical research by optimizing the quality of lay summaries.

## 2. Materials and Methods

This study was conducted as a retrospective analysis of articles published in *Current Oncology* between 1 July 2022, and 30 June 2024. The primary objective was to evaluate the effectiveness of ChatGPT-4 in generating high-quality lay summaries using predefined prompt designs. Ethical approval for this study was obtained from the Institutional Review Board of the University of Regensburg (protocol code 24-3883-104, date of approval: 2 September 2024).

### 2.1. Article Selection

Articles were identified through a systematic search in the PubMed database using the following query: “(Curr Oncol) AND (prostate cancer OR prostate neoplasm OR prostate carcinoma)”. The inclusion criteria were as follows: articles with abstracts and keywords, a primary focus on prostate cancer, and publication within the specified timeframe. Exclusion criteria included articles unrelated to prostate cancer, those lacking abstracts or keywords, and publications outside the defined period. Of 133 initially identified articles, 80 met the inclusion criteria and were included in the analysis.

The use of publicly available abstracts for scientific purposes adheres to the principles of ‘fair use’ as outlined in the U.S. Copyright Act (17 U.S. Code § 107) and the German Copyright Act (UrhG, § 51). All utilized works have been appropriately cited and referenced (see Supplementary Data S1).

### 2.2. Development of Standardized Prompts for Data Input into ChatGPT-4

Two distinct prompts were developed within a Delphi process, with each stage of their refinement following a structured and iterative approach. Initial suggestions proposed by one author (MM) underwent systematic refinement through multiple rounds of discussion with two other authors (MH and ER) to optimize the instructional framework for ChatGPT-4 in generating lay summaries. This refinement process was guided by principles of prompt engineering and adaptive iteration [16], ensuring that the final prompts were empirically tested and optimized for clarity, coherence, and effectiveness. The resulting outputs were systematically evaluated using the outcome metrics defined in Section 2.3 and Section 2.4, ensuring that the final two prompts were developed through a rigorous, evidence-based process.

To assess how variations in prompt design influence the generated summaries, two versions were established: (1) Simple prompt—this prompt instructed ChatGPT-4 to generate a single-paragraph lay summary in plain language. The summary was required to focus on the study’s purpose, methodology, main findings, and practical implications, while avoiding technical jargon and abbreviations. (2) Extended prompt—the extended prompt was derived from the simple prompt by incorporating the short paragraph ‘The Simple Summary should be crafted with a focus on maximizing readability, aiming for the highest possible Flesch Kincaid Reading Ease score’ and the partial sentence ‘at a 6th grade reading level’, while keeping all other elements unchanged (Table 1). This prompt provided more detailed instructions, aligning with Good Lay Summary Practice guidelines [5]. It emphasized the use of accessible language, readability metrics (targeting a Flesch–Kincaid Reading Ease score of ≥30), and a comprehensive yet concise narrative structure. Each article was processed using both prompts, with a new ChatGPT-4 session initiated for each input. The summaries generated were evaluated independently.

### 2.3. Readability Assessment

The readability of the generated summaries was assessed using multiple metrics, including the Flesch–Kincaid Reading Ease (FKRE), Flesch–Kincaid Grade Level (FKGL), Gunning Fog Score (GFS), Smog Index (SI), Coleman–Liau Index (CLI), and Automated Readability Index (ARI). The metrics and readability indices were calculated using the WebFX Readability Test Tool (https://www.webfx.com/tools/read-able/, accessed on 23 December 2024). To obtain the readability scores, the WebFX website was accessed, and the ‘Enter Text’ option was selected. The lay summary under evaluation was inserted into the designated window, and the ‘Calculate Readability’ button was clicked. The resulting metrics were then manually recorded in the data sheet.

### 2.4. Content Assessment of the Lay Summaries and Final Evaluation

The quality of the generated lay summaries was evaluated by an independent rater (MM) with extensive scientific expertise (author of >250 peer-reviewed publications). The evaluation employed a 5-point Likert scale to assess alignment with the abstract and keywords, ranging from 1 (very poor) to 5 (excellent). The evaluation focused explicitly on the consistency of the lay summary with the original data (title, abstract, and keywords), rather than on the scientific quality of the original paper or the generated lay summary itself. Detailed evaluation criteria are presented in Table 2.

A lay summary was considered flawless only if it met three criteria: sufficiently high readability (FKRE ≥ 30), high content quality (assessments scoring ≥ 4), and strict adherence to formal requirements (maximum word count 150 and minimum word count no more than 20% below 150, which corresponds to 120 words). Any summary that was not deemed flawless was classified as insufficient.

### 2.5. Statistical Analysis

Statistical analysis was conducted using SPSS version 29.0 (IBM, Armonk, NY, USA). Testing for normal distribution was conducted using the Shapiro–Wilk test (data available on request). Descriptive statistics were reported as absolute and relative frequencies for categorical variables and as medians with interquartile ranges (IQRs) for continuous variables where appropriate. To investigate differences between the respective simple summaries (ChatGPT-4 simple prompt vs. ChatGPT-4 extended prompt), a paired *t*-test was used for normally distributed continuous variables, and a Wilcoxon signed-rank test was applied for non-normally distributed or ordinal data. *p*-values < 0.05 indicated statistical significance. All analyses were considered two-tailed. Graphs were created using ChatGPT-4 (OpenAI, San Francisco, CA, USA).

## 3. Results

### 3.1. Article Characteristics

**Figure 1:**
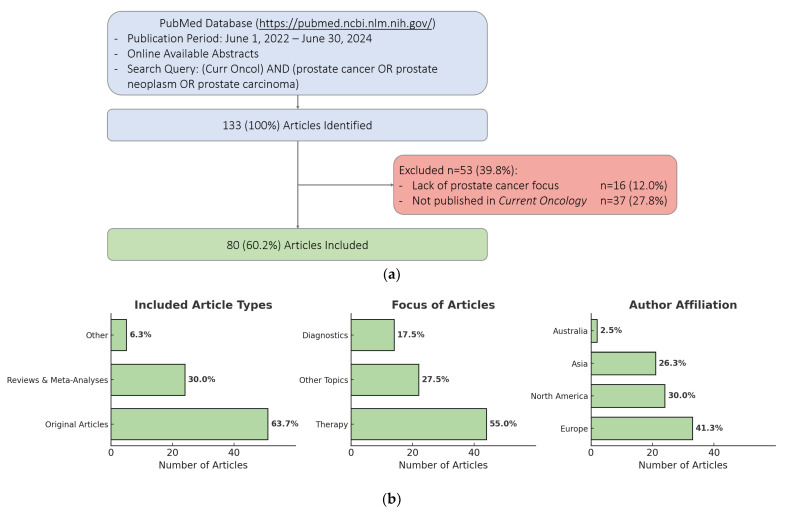
(**a**) Flowchart of the article selection process. (**b**) Article characteristics.

Between 1 June 2022 and 30 June 2024, a total of 133 articles were screened. Sixteen articles (12.0%) were excluded due to a lack of specific focus on prostate cancer. An additional 37 articles (27.8%) were excluded because they were not published in *Current Oncology*. No articles were excluded due to missing abstracts or keywords. Ultimately, 80 articles (60.2%) met the inclusion criteria and were incorporated into this study (Figure 1a). Of those, 19 articles (23.8%) were published in 2022, 47 (58.8%) in 2023, and 14 (17.5%) in 2024. Original articles accounted for 51 (63.7%), reviews and meta-analyses for 24 (30%), and other article types accounted for 5 (6.3%). Fourteen articles (17.5%) focused on the diagnostics of prostate cancer, forty-four (55%) on prostate cancer therapy, and twenty-two (27.5%) on other topics. The affiliation of the corresponding author was in Europe in 33 cases (41.3%), North America in 24 cases (30%), Asia in 21 cases (26.3%), and Australia in 2 cases (2.5%) (Figure 1b).

### 3.2. Evaluation of the Lay Summaries

**Figure 2:**
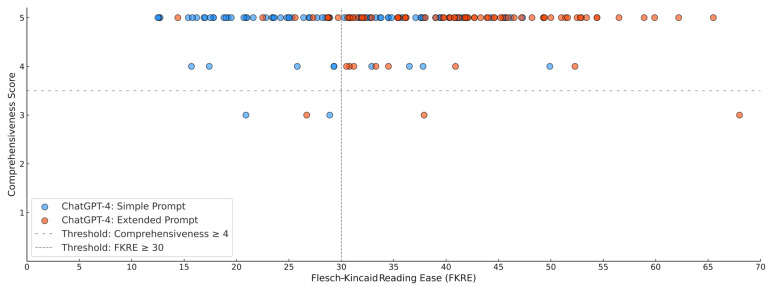
Distribution of Flesch–Kincaid Reading Ease (FKRE) scores for ChatGPT-4-generated lay summaries, comparing simple prompts (blue) and extended prompts (red) across comprehensiveness scores. Transparency highlights overlapping data points. Thresholds are indicated by dashed lines.

The descriptive analyses regarding text length metrics, readability scores, content quality, and overall evaluation are depicted in Table 3. Table 3 also provides a detailed description of the statistical analyses comparing the lay summaries generated by ChatGPT-4 using the simple prompt with those generated using the extended prompt. Figure 2 illustrates the FKRE achieved in relation to the respective comprehensiveness score, categorized by the prompt used.

Compared to the ChatGPT-4 simple prompt, the FKRE scores were significantly higher for the lay summaries generated with the extended ChatGPT-4 prompt (40.9 vs. 29.1; *p* < 0.001). This pattern was consistent across other readability metrics, which also indicated lower reading age or grade level requirements for the extended-prompt ChatGPT-4 generated summaries compared to the simple-prompt ChatGPT-4 generated lay summaries.

In terms of comprehensiveness, both the simple (5, IQR: 5-5) and extended (5, IQR: 5-5) ChatGPT-4 prompts generated lay summaries with excellent comprehensiveness. A total of 78 (97.5%) simple-prompt lay summaries and 77 (96.3%) extended-prompt lay summaries achieved sufficiently high comprehensiveness, defined as a comprehensiveness score ≥ 4. There was no significant difference between the comprehensiveness of the lay summaries generated by the two different prompts (*p* = 0.963).

For the overall evaluation, the proportion of summaries classified as insufficient was assessed. Summaries were deemed insufficient if they did not meet the predefined criteria for a flawless lay summary, which included adequate word count, FKRE score, and comprehensiveness score. The simple ChatGPT-4 prompt generated lay summaries yielded a significantly higher proportion of insufficient lay summaries compared to the lay summaries generated by the extended ChatGPT-4 prompt (52.5% vs. 13.8%; *p* < 0.001).

## 4. Discussion

This study provides compelling evidence for the potential of ChatGPT-4 in generating high-quality lay summaries for prostate cancer research, emphasizing the critical role of tailored prompt engineering. The findings reveal that the extended prompt significantly enhances readability, accessibility, and adherence to predefined quality metrics compared to the simple prompt. Specifically, the extended prompt achieved superior FKRE scores (median: 40.9 vs. 29.1, *p* < 0.001) and was more likely to produce summaries that met all quality thresholds, including readability and content alignment (86.2% vs. 47.5%, *p* < 0.001). These results underscore the transformative potential of large language models (LLMs) in medical communication, particularly when guided by well-structured and domain-specific prompts.

A notable strength of this study is its exclusive focus on prostate cancer articles published in *Current Oncology*, a high-impact journal within the field of oncology. By analyzing a consecutive series of 80 articles published over two years, this study provides a robust and focused evaluation of ChatGPT-4’s capabilities. This approach allowed for the systematic assessment of readability metrics and content alignment within a controlled dataset, demonstrating the scalability of LLMs in generating consistent and reliable outputs for a specific oncological domain. Moreover, the use of objective readability indices, such as the FKRE and related metrics, offers a quantitative framework for evaluating the accessibility of AI-generated summaries, setting a benchmark for future studies in this area.

The findings align with prior research, such as the work of Eppler et al. [13] and Shyr et al. [14], who highlighted the importance of prompt design in leveraging LLMs for medical applications. However, this study advances the field by providing a detailed, quantitative comparison of two distinct prompt designs, demonstrating that even minor adjustments to input prompts can yield substantial improvements in output quality. This insight is particularly relevant given the increasing adoption of LLMs in academic and clinical settings, where the demand for accurate and accessible communication tools continues to grow.

While this study employed a zero-shot prompting approach to generate lay summaries using ChatGPT-4, most teams in the BioLaySumm Shared Task 2024 adopted more sophisticated computational strategies, integrating preprocessing techniques, extractive and abstractive summarization, and fine-tuning of LLMs [15,17,18,19]. These teams also leveraged domain-specific training datasets to optimize their models for enhanced performance [15,17,18,19].

Our findings indicate that, even without fine-tuning or specialized training data, a strategically designed zero-shot prompt can produce lay summaries with high readability and factual accuracy, offering a computationally efficient and accessible alternative. This approach is particularly advantageous for medical scientists, as it allows for the generation of high-quality lay summaries without requiring advanced computational expertise or access to extensive computational resources—barriers that often limit the practical implementation of fine-tuned models in clinical and research settings.

Despite its strengths, this study has several limitations that warrant consideration. First, the exclusive focus on prostate cancer articles limits the generalizability of the findings to other oncological or medical domains. While this targeted approach enhances the internal validity of this study, future research should expand the scope to include other cancer types or broader medical disciplines, as different disease contexts may present unique challenges in generating lay summaries. Second, the analysis was confined to a two-year publication window, which, while practical for feasibility, restricts the longitudinal applicability of the findings. A broader timeframe could provide additional insights into temporal trends in readability and content quality. Third, this study did not explore variability in outputs across multiple iterations of the same prompt. Given that LLMs like ChatGPT-4 can produce slightly different outputs depending on input phrasing and contextual factors, future investigations should evaluate the consistency and reliability of AI-generated summaries across repeated prompt entries. Furthermore, while this study demonstrated the feasibility of employing LLMs for generating lay summaries, it did not include a comparative arm involving manually crafted summaries by human experts. A direct comparison between AI-generated and human-written summaries, particularly regarding patient comprehension and perceived credibility, would provide critical insights into the practical utility of this technology. Ethical considerations also merit attention. While this study exclusively utilized open access abstracts as input data, broader applications of LLMs in healthcare must address concerns regarding data privacy, algorithmic bias, and the financial accessibility of AI tools. We propose the establishment of interdisciplinary governance committees that include ethicists, clinicians, and AI specialists to develop standards for transparency, accountability, and quality assurance in AI-generated medical content. Additionally, the integration of LLMs into medical communication workflows should be accompanied by clear guidelines for human oversight to ensure the accuracy and integrity of the generated content. As highlighted in previous studies [13,14,20], even the most advanced LLMs require human validation to mitigate potential inaccuracies or biases. Developing transparent reporting standards for AI-generated content in medical literature could help establish trust and ensure accountability in its application.

Future research should build on these findings by exploring the impact of iterative prompt refinement and incorporating patient feedback into the evaluation of lay summaries. Comparative analyses with manually crafted summaries, randomized controlled trials, and studies involving broader datasets will be essential for validating the scalability and reliability of LLMs in diverse medical contexts. In particular, studies assessing the real-world usability of AI-generated lay summaries in patient education and clinical decision support could bridge the gap between theoretical research and applied medical practice. Implementing structured frameworks for AI-assisted lay summarization could facilitate the broader adoption of LLMs in scientific publishing and clinical education, ensuring that these technologies are effectively leveraged to enhance knowledge dissemination.

To summarize, this study demonstrates that ChatGPT-4, when guided by tailored prompts, represents a valuable tool for improving the accessibility of prostate cancer research. By addressing the identified limitations and expanding the scope of analysis, future research can unlock the full potential of LLMs in transforming medical communication, ultimately enhancing public engagement and understanding of scientific literature.

## 5. Conclusions

This study demonstrates that ChatGPT-4, when guided by tailored prompts, can generate high-quality lay summaries with enhanced readability and factual accuracy in prostate cancer research. By optimizing prompt design, LLMs can be systematically leveraged to improve the accessibility of complex medical literature for broader audiences. Future research should validate these findings across diverse medical domains, assess inter-prompt variability in summary generation, and directly compare AI-generated lay summaries with those authored by human experts. Furthermore, incorporating patient feedback and establishing standardized guidelines for prompt engineering will be essential to ensure that AI-generated content meets not only linguistic and technical standards but also the informational needs of non-expert audiences.

## Figures and Tables

**Table 1:** ChatGPT-4 input prompts for creating a lay summary.

Simple Prompt	Extended Prompt
Dear ChatGPT-4, I kindly request your assistance in crafting a Simple Summary as part of a scientific study. The Simple Summary must adhere to the following guidelines: It should be written in one paragraph, in layman’s terms, to explain why the research is being suggested, what the authors aim to achieve, and how the findings from this research may impact the research community. Please use as few abbreviations as possible, and do not cite references in the Simple Summary. The Simple Summary should not exceed 150 words.	Dear ChatGPT-4, I kindly request your assistance in crafting a Simple Summary as part of a scientific study. The Simple Summary must adhere to the following guidelines: It should be written in one paragraph, in layman’s terms, to explain why the research is being suggested, what the authors aim to achieve, and how the findings from this research may impact the research community. Please use as few abbreviations as possible, and do not cite references in the Simple Summary. The Simple Summary should not exceed 150 words.
	The Simple Summary should be crafted with a focus on maximizing readability, aiming for the highest possible Flesch Kincaid Reading Ease score.
To provide you with the necessary context for creating this Simple Summary, I will supply you with the study title, a scientifically accurate abstract (not in Layman’s terms), and the relevant keywords. Study title: “…” Scientifically accurate abstract: “…” Keywords: “…”	To provide you with the necessary context for creating this Simple Summary, I will supply you with the study title, a scientifically accurate abstract (not in Layman’s terms), and the relevant keywords. Study title: “…” Scientifically accurate abstract: “…” Keywords: “…”
Please note: Summarize this unstructured abstract (simple summary) with at most 150 words in lay language, highlighting the study purpose, methods, key findings, and practical importance of these findings for the general public. Additionally, be aware that the Simple Summary should not exceed 150 words, but it should make the most of this limit.	Please note: Summarize this unstructured abstract (simple summary) with at most 150 words in lay language at a 6th grade reading level, highlighting the study purpose, methods, key findings, and practical importance of these findings for the general public. Additionally, be aware that the Simple Summary should not exceed 150 words, but it should make the most of this limit.

**Table 2:** Description of the 5-point Likert scale used for the evaluation of the content quality (comprehensiveness score) of the lay summaries.

Score	Explanation
1—very poor	The lay summary contains significant factual errors and/or diverges substantially from the scientific abstract. Essential information is missing, which severely compromises its clarity and accuracy.
2—poor	The lay summary has multiple factual inaccuracies and diverges in certain areas from the scientific abstract. Some key information is missing, diminishing its overall effectiveness.
3—acceptable	The lay summary is mostly accurate but contains minor factual inaccuracies or omissions. It generally aligns with the scientific abstract, though some details could be more precise or comprehensive.
4—good	The lay summary is factually accurate and largely consistent with the scientific abstract. Only minor, non-essential information may be missing or slightly simplified.
5—excellent	The lay summary is completely accurate, fully aligns with the scientific abstract, and includes all essential information. It conveys the content clearly and effectively, without omitting any important details.

**Table 3:** Descriptive data regarding length metrics, readability scores, and content quality for the lay summaries created by ChatGPT-4 (simple prompt) and ChatGPT-4 (extended prompt), respectively.

Parameter		ChatGPT-4 Simple Prompt	ChatGPT-4 Extended Prompt	*p*-Values
Text length metrics				
	Sentences; median (IQR)	6 (5–6)	6 (6–7)	**<0.001**
	Words; median (IQR)	135 (126–140)	130 (121.3–138)	**<0.001**
	Complex words; median (IQR)	29 (25–32)	23 (16.3–26)	**<0.001**
	Percent of complex words; median (IQR)	21.1 (18.1–24.2)	17.4 (13.4–20.5)	**<0.001**
	Average words per sentence; median (IQR)	23.8 (21.2–26.2)	21.1 (19.3–23.2)	**<0.001**
	Average syllables per word; median (IQR)	1.8 (1.7–1.9)	1.7 (1.6–1.8)	**<0.001**
Readability Scores				
	Flesch–Kincaid Reading Ease (FKRE); median (IQR)	29.1 (20.9–37)	40.9 (32.2–49)	**<0.001**
	Flesch–Kincaid Grade Level; median (IQR)	14.8 (13.8–16.5)	12.8 (11.8–14.1)	**<0.001**
	Gunning Fog Score; median (IQR)	17.6 (16.3–19.6)	15.3 (13.7–16.7)	**<0.001**
	Smog Index; median (IQR)	12.7 (11.8–14.1)	11.1 (9.8–12)	**<0.001**
	Coleman–Liau Index; median (IQR)	17.3 (15.9–18.7)	15.6 (14.5–17.1)	**<0.001**
	Automated Readability Index; median (IQR)	16.9 (15.3–18.6)	14 (13.1–16.1)	**<0.001**
	Reading age in years; median (IQR)	21.5 (20.5–22.9)	19.5 (18.5–20.5)	**<0.001**
Content quality and overall evaluation				
	Comprehensiveness score; median (IQR)	5 (5-5)	5 (5-5)	0.963
	Comprehensiveness score < 4; n (%)	2 (2.5)	3 (3.8)	0.564
	FKRE < 30; n (%)	42 (52.5)	9 (11.3)	**<0.001**
	Wrong word count; n (%)	1 (1.3)	1 (1.3)	1.000
	Insufficient quality of the patient summary; n (%)	42 (52.5)	11 (13.8)	**<0.001**

Bold letters indicate statistical significance. Total n = 80.

## Data Availability

The original contributions presented in this study are included in the article/Supplementary Materials. Further inquiries can be directed to the corresponding author.

## Supplementary Materials

The following supporting information can be downloaded at https://www.mdpi.com/article/10.3390/curroncol32020102/s1—Supplementary Data S1: Details of included articles, abstracts, keywords, and ChatGPT-4-generated lay summaries with readability metrics, word counts, and comprehension scores.
