# Supplementary material for: Leveraging Large Language Models for High-Quality Lay Summaries: Efficacy of ChatGPT-4 with Custom Prompts in a Consecutive Series of Prostate Cancer Manuscripts
Source: Curr Oncol. 2025 Feb 11;32(2):102. doi: 10.3390/curroncol32020102 (PMC11854015; doi:10.3390/curroncol32020102)
Supplement: Supplementary file 1 [file curroncol-32-00102-s001.zip › curroncol-3418396-supplementary-proofread.pdf]

Leveraging Large Language Models for High-Quality Lay Summaries: Efficacy of ChatGPT-4 with Custom Prompts in a Consecutive Series of Prostate Cancer Manuscripts

Emily Rinderknecht [1,2], Anna Schmelzer [3,†], Anton Kravchuk [3], Christopher Goßler [1], Johannes Breyer [1], Christian Gilfrich [3], Maximilian Burger [1], Simon Engelmann [1], Veronika Saberi [1], Clemens Kirschner [1], Dominik von Winning [3], Roman Mayr [1], Christian Wülfing [2,4], Hendrik Borgmann [2,5], Stephan Buse [6], Maximilian Haas [1#] and Matthias May [2,3,*,‡]

[1] Department of Urology, St. Josef Medical Center, University of Regensburg, 93053 Regensburg, Germany

[2] Working Group on Artificial Intelligence and Digitalization of the German Society of Urology

[3] Department of Urology, St. Elisabeth Hospital Straubing, 94315 Straubing, Germany

[4] Department of Urology, Asklepios Klinik Altona, 22763 Hamburg, Germany

[5] Department of Urology, Brandenburg Medical School Theodor Fontane (MHB), 14770 Brandenburg an der Havel, Germany

[6] Department of Urology, Alfried Krupp Krankenhaus, 45131 Essen, Germany

* Correspondence: matthias.may@klinikum-straubing.de

† Current address: Department of Urology, Nuremberg General Hospital, Paracelsus Medical University, 90419 Nuremberg, Germany

‡ These authors contributed equally to this work

Supplementary Data S1:

Details of Included Articles, Abstracts, Keywords, and ChatGPT-4-Generated Lay Summaries with Readability Metrics, Word Counts, and Comprehension Scores.

Manuscript 1

Deluce JE, Cardenas L, Lalani AK, Maleki Vareki S, Fernandes R. Emerging Biomarker-Guided Therapies in Prostate Cancer. Curr Oncol. 2022 Jul 18;29(7):5054-5076. doi: 10.3390/curroncol29070400. PMID: 35877260; PMCID: PMC9319825.

Abstract

Prostate cancer remains one of the leading causes of cancer death in men worldwide. In the past decade, several new treatments for advanced prostate cancer have been approved. With a wide variety of available drugs, including cytotoxic agents, androgen receptor axis-targeted therapies, and alpha-emitting radiation therapy, identifying their optimal sequencing remains a challenge. Progress in the understanding of the biology of prostate cancer has provided an opportunity for a more refined and personalized treatment selection process. With the advancement of molecular sequencing techniques, genomic precision through the identification of potential treatment targets and predictive biomarkers has been rapidly evolving. In this review, we discussed biomarker-driven treatments for advanced prostate cancer. First, we presented predictive biomarkers for established, global standard treatments for advanced diseases, such as chemotherapy and androgen receptor axis-targeted agents. We also discussed targeted agents with recent approval for special populations, such as poly ADP ribose polymerase (PARP) inhibitors in patients with metastatic castrate-resistant prostate cancer with homologous recombination repair-deficient tumors, pembrolizumab in patients with high levels of microsatellite instability or high tumor mutational burden, and prostate-specific membrane antigen (PSMA) directed radioligand theragnostic treatment for PSMA expressing tumors. Additionally, we discussed evolving treatments, such as cancer vaccines, chimeric antigen receptor T-cells (CAR-T), Bispecific T-cell engagers (BiTEs), other targeted agents such as AKT inhibitors, and various combination treatments. In summary, advances in molecular genetics have begun to propel personalized medicine forward in the management of advanced prostate cancer, allowing for a more precise, biomarker-driven treatment selection with the goal of improving overall efficacy.

Keywords: PARP inhibitors; immunotherapy; predictive biomarkers; prostate cancer; theranostics

Lay Summary (simple Prompt)

This study explores new, more personalized approaches to treating advanced prostate cancer based on specific biomarkers—molecules that can indicate the presence or progression of cancer. By understanding the genetic makeup of prostate cancer, doctors can better choose from a range of available treatments, such as chemotherapy, targeted hormone therapies, or newer options like PARP inhibitors and immunotherapy. The study reviews various emerging therapies, including cancer vaccines and innovative cellular treatments like CAR-T cells. These advancements aim to match patients with the most effective treatments based on their unique cancer characteristics, improving outcomes and potentially reducing unnecessary side effects. This personalized approach represents a significant step forward in managing advanced prostate cancer, offering hope for better, more targeted treatment strategies in the future.

Evaluation of the Lay Summary

**Assessment Category**	**Score**	**Assessment Category**	**Score**	**Assessment Category**	**Score**
Comprehension Score	5	Gunning Fog Score	20.9	Words	123
Accuracy Score	1	SMOG Index	15.0	Complex words	34
Reading Age (years)	23.0	Coleman Liau Index	21.2	Percent of complex words	27.6
Flesch Kincaid Reading Ease	12.7	Automated Readability Index	20.4	Average words per sentence	24.6
Flesch Kincaid Grade Level	17.6	Sentences	5	Average syllables per word	2.0

Lay Summary (extended Prompt)

Prostate cancer is a major cause of cancer deaths in men, and new treatments have recently become available. This study focuses on the use of biomarkers to guide treatment choices for advanced prostate cancer. Biomarkers are biological indicators that can help doctors choose the most effective treatment for each patient. The review highlights how certain biomarkers can predict how patients will respond to therapies like chemotherapy and drugs targeting androgen receptors. It also explores newer treatments, such as PARP inhibitors for patients with specific genetic mutations, immunotherapies for those with high tumor mutation rates, and PSMA-targeted therapies. The goal of this research is to make prostate cancer treatment more personalized, ensuring each patient receives the best possible care based on their unique cancer profile.

Evaluation of the Lay Summary

**Assessment Category**	**Score**	**Assessment Category**	**Score**	**Assessment Category**	**Score**
Comprehension Score	5	Gunning Fog Score	16.3	Words	125
Accuracy Score	0	SMOG Index	11.8	Complex words	25
Reading Age (years)	20.5	Coleman Liau Index	17.5	Percent of complex words	20.0
Flesch Kincaid Reading Ease	35.4	Automated Readability Index	15.6	Average words per sentence	20.8
Flesch Kincaid Grade Level	13.5	Sentences	6	Average syllables per word	1.8

Manuscript 2

Oehler C, Roehner N, Sumila M, Curschmann J, Storelli F, Zwahlen DR, Schneider U. Intrafraction Prostate Motion Management for Ultra-Hypofractionated Radiotherapy of Prostate Cancer. Curr Oncol. 2022 Aug 31;29(9):6314-6324. doi: 10.3390/curroncol29090496. PMID: 36135065; PMCID: PMC9497512.

Abstract

Purpose: Determine the time-dependent magnitude of intrafraction prostate displacement and a cutoff for the tracking decision. Methods: Nine patients with localized prostate cancer were treated with ultra-hypofractionated radiotherapy (CyberKnife) with fiducial markers. Exact tract kV/kV imaging was used with an average interval of 19−92 s. A Gaussian distribution was calculated for the x-, y-, and z-directions (σx,y,z). The variation of prostate motion (μσ) was obtained by averaging the patients' specifics, and the safety margin was calculated to be MAB = WYm + WBSs. Results: The calculated PTV safety margins were as follows: at 40 s: 0.55 mm (L/r), 0.85 mm (a/p), and 1.05 mm (s/i); at 60 s: 0.9 mm (L/r), 1.35 mm (a/p), and 1.55 mm (s/i); at 100 s: 1.5 mm (L/r), 2.3 mm (a/p), and 2.6 mm (s/i); at 150 s: 1.9 mm (L/r), 3.1 mm (a/p), and 3.6 mm (s/i); at 200 s: 2.2 mm (L/r), 3.8 mm (a/p), and 4.2 mm (s/i); and at 300 s: 2.6 mm (L/r), 5.3 mm (a/p), and 5.6 mm (s/i). A tracking cutoff of 2.5 min seemed reasonable. In order to achieve an accuracy of < 1 mm, tracking with < 50 s intervals was necessary. Conclusions: For ultra-hypofractionated radiotherapy of the prostate with treatment times > 2.5 min, intrafraction motion management is recommended.

Keywords: IGRT; MR-based adaptive radiotherapy; PTV margin; prostate cancer; prostate motion; radiation therapy; radiopaque fiducial marker; stereotactic; tracking; ultra-hypofractionation.

Lay Summary (simple Prompt)

This study investigates how much the prostate moves during ultra-hypofractionated radiotherapy, a precise form of radiation treatment for prostate cancer, and how often this movement should be tracked to ensure accurate targeting. Researchers monitored nine patients using special markers and frequent imaging to measure prostate displacement over time. They found that the prostate moves progressively more the longer the treatment lasts, suggesting that motion tracking should occur at intervals of less than 50 seconds to maintain accuracy within 1 millimeter. For treatments longer than 2.5 minutes, managing this movement is critical to delivering effective radiation while minimizing damage to surrounding healthy tissue. The findings support the need for frequent tracking during radiation therapy to improve treatment outcomes for prostate cancer patients.

Evaluation of the Simple Summary

**Assessment Category**	**Score**	**Assessment Category**	**Score**	**Assessment Category**	**Score**
Comprehension Score	4	Gunning Fog Score	16.7	Words	123
Accuracy Score	1	SMOG Index	12.0	Complex words	26
Reading Age (years)	21.5	Coleman Liau Index	19.0	Percent of complex words	21.1
Flesch Kincaid Reading Ease	25.8	Automated Readability Index	16.7	Average words per sentence	20.5
Flesch Kincaid Grade Level	14.8	Sentences	6	Average syllables per word	1.9

Lay Summary (extended Prompt)

This study focused on how to manage prostate movement during ultra-hypofractionated radiotherapy, a treatment for prostate cancer that uses very high doses of radiation in fewer sessions. Prostate movement during treatment can affect the accuracy of radiation delivery. Researchers tracked prostate movement in nine patients using markers and imaging. They found that prostate displacement increased over time, and calculated safety margins to ensure accurate treatment. For treatments longer than 2.5 minutes, managing this movement is necessary. To keep movement within 1 millimeter, the prostate should be tracked every 50 seconds or less. These findings help improve the precision of prostate cancer treatment, potentially leading to better outcomes.

Evaluation of the Simple Summary

**Assessment Category**	**Score**	**Assessment Category**	**Score**	**Assessment Category**	**Score**
Comprehension Score	4	Gunning Fog Score	13.9	Words	109
Accuracy Score	1	SMOG Index	9.9	Complex words	23
Reading Age (years)	18.5	Coleman Liau Index	18.2	Percent of complex words	21.1
Flesch Kincaid Reading Ease	30.8	Automated Readability Index	12.6	Average words per sentence	13.6
Flesch Kincaid Grade Level	12.3	Sentences	8	Average syllables per word	1.9

Manuscript 3

Dahl AA, Fosså SD. High Neuroticism Is Related to More Overall Functional Problems and Lower Function Scores in Men Who Had Surgery for Non-Relapsing Prostate Cancer. Curr Oncol. 2022 Aug 17;29(8):5823-5832. doi: 10.3390/curroncol29080459. PMID: 36005197; PMCID: PMC9406934.

Abstract

The personality trait of neuroticism is associated with adverse health outcomes after cancer treatment, but few studies concern men treated for prostate cancer. We examined men with high and low neuroticism treated with radical prostatectomy for curable prostate cancer without relapse. We compared overall problems and domain summary scores (DSSs) between these groups, and if high neuroticism at pre-treatment was a significant predictor of overall problems and DSSs at follow-up. A sample of 462 relapse-free Norwegian men self-rated neuroticism, overall problems, and DSSs by the EPIC-26 before surgery and at three years' follow-up. Twenty-one percent of the sample had high neuroticism. Patients with high neuroticism reported significantly more overall problems and DSSs at pre-treatment. At follow-up, only overall bowel problems and urinary irritation/obstruction and bowel DSSs were different. High neuroticism was a significant predictor of overall bowel problems and bowel and irritation/obstruction DSSs at follow-up. High neuroticism at pre-treatment was significantly associated with a higher rate of overall problems both at pre-treatment and follow-up and had some significant predictions concerning bowel problems and urinary obstruction at follow-up. Screening for neuroticism at pre-treatment could identify patients in need of more counseling concerning later adverse health outcomes.

Keywords: generalized estimation equations; neuroticism; overall problems; prospective study; prostate cancer; radical prostatectomy.

Lay Summary (simple Prompt)

This study explored how the personality trait of neuroticism, characterized by anxiety and emotional instability, affects health outcomes in men treated for prostate cancer. Researchers followed 462 men who underwent surgery for non-relapsing prostate cancer, comparing those with high and low levels of neuroticism. They found that men with high neuroticism reported more overall problems both before and three years after surgery, especially related to bowel and urinary issues. The findings suggest that neuroticism can predict long-term health challenges after prostate cancer treatment. Identifying patients with high neuroticism before surgery could help healthcare providers offer targeted counseling and support, potentially improving their quality of life and reducing future health problems.

Evaluation of the Simple Summary

**Assessment Category**	**Score**	**Assessment Category**	**Score**	**Assessment Category**	**Score**
Comprehension Score	4	Gunning Fog Score	19.7	Words	112
Accuracy Score	1	SMOG Index	14.1	Complex words	30
Reading Age (years)	23.5	Coleman Liau Index	19.8	Percent of complex words	26.8
Flesch Kincaid Reading Ease	15.7	Automated Readability Index	18.2	Average words per sentence	22.4
Flesch Kincaid Grade Level	16.6	Sentences	5	Average syllables per word	2.0

Lay Summary (extended Prompt)

This study examined how the personality trait of neuroticism affects health outcomes in men who had surgery for prostate cancer. Neuroticism is linked to negative health effects, and researchers wanted to see if men with high neuroticism faced more problems after surgery. They followed 462 men who had prostate cancer surgery, measuring their neuroticism levels before treatment and tracking their health issues for three years. Twenty-one percent of the men had high neuroticism, and these men reported more overall problems and specific issues like bowel and urinary problems before and after surgery. The study found that high neuroticism at the start was a strong predictor of these problems at follow-up. The findings suggest that identifying men with high neuroticism before surgery could help provide them with additional counseling to better manage potential health challenges after treatment.

Evaluation of the Simple Summary

Assessment Category	Score	Assessment Category	Score	Assessment Category	Score
Comprehension Score	4	Gunning Fog Score	16.7	Words	138
Accuracy Score	0	SMOG Index	12.0	Complex words	26
Reading Age (years)	20.5	Coleman Liau Index	16.3	Percent of complex words	18.8
Flesch Kincaid Reading Ease	33.3	Automated Readability Index	15.8	Average words per sentence	23.0
Flesch Kincaid Grade Level	14.3	Sentences	6	Average syllables per word	1.8

Manuscript 4

Liu J, Pan S, Dong L, Wu G, Wang J, Wang Y, Qian H, Dong B, Pan J, Zhu Y, Xue W. The Diagnostic Value of PI-RADS v2.1 in Patients with a History of Transurethral Resection of the Prostate (TURP). Curr Oncol. 2022 Sep 5;29(9):6373-6382. doi: 10.3390/curroncol29090502. PMID: 36135071; PMCID: PMC9497547.

Abstract

To explore the diagnostic value of the Prostate Imaging–Reporting and Data System version 2.1 (PI-RADS v2.1) for clinically significant prostate cancer (CSPCa) in patients with a history of transurethral resection of the prostate (TURP), we conducted a retrospective study of 102 patients who underwent systematic prostate biopsies with TURP history. ROC analyses and logistic regression analyses were performed to demonstrate the diagnostic value of PI-RADS v2.1 and other clinical characteristics, including PSA and free/total PSA (F/T PSA). Of 102 patients, 43 were diagnosed with CSPCa. In ROC analysis, PSA, F/T PSA, and PI-RADS v2.1 demonstrated significant diagnostic value in detecting CSPCa in our cohort (AUC 0.710 (95%CI 0.608–0.812), AUC 0.768 (95%CI 0.676–0.860), AUC 0.777 (95%CI 0.688–0.867), respectively). Further, PI-RADS v2.1 scores of the peripheral and transitional zones were analyzed separately. In ROC analysis, PI-RADS v2.1 remained valuable in identifying peripheral-zone CSPCa (AUC 0.780 (95%CI 0.665–0.854; $p < 0.001$)) while having limited capability in distinguishing transitional zone lesions (AUC 0.533 (95%CI 0.410–0.557; $p = 0.594$)). PSA and F/T PSA retain significant diagnostic value for CSPCa in patients with TURP history. PI-RADS v2.1 is reliable for detecting peripheral-zone CSPCa but has limited diagnostic value when assessing transitional zone lesions.

Keywords: multiparametric magnetic resonance imaging; prostate cancer; prostate-specific antigen; transurethral resection of the prostate.

Lay Summary (simple Prompt)

This study investigated how well a specific imaging system, called PI-RADS v2.1, can detect significant prostate cancer in patients who have previously undergone a procedure known as transurethral resection of the prostate (TURP). The researchers analyzed data from 102 patients who had systematic prostate biopsies after TURP. They found that the PI-RADS v2.1 imaging system, along with PSA and free/total PSA levels, is useful for diagnosing cancer in the outer areas of the prostate (peripheral zone) but not as effective for the central areas (transitional zone). This suggests that while PI-RADS v2.1 is a reliable tool for identifying certain types of prostate cancer in patients with a history of TURP, its limitations should be considered, particularly for cancers in the transitional zone. These findings may help improve diagnostic strategies and outcomes for these patients.

Evaluation of the Simple Summary

Assessment Category	Score	Assessment Category	Score	Assessment Category	Score
Comprehension Score	5	Gunning Fog Score	15.0	Words	140
Accuracy Score	0	SMOG Index	10.8	Complex words	28
Reading Age (years)	18.5	Coleman Liau Index	14.4	Percent of complex words	20.0
Flesch Kincaid Reading Ease	41.6	Automated Readability Index	11.5	Average words per sentence	17.5
Flesch Kincaid Grade Level	11.8	Sentences	8	Average syllables per word	1.7

Lay Summary (extended Prompt)

This study looked at how well a test called PI-RADS v2.1 can detect significant prostate cancer in men who have had surgery to remove part of their prostate (TURP). We reviewed the medical records of 102 men who had prostate biopsies after TURP. We focused on how well PI-RADS v2.1, along with PSA levels (a protein linked to prostate cancer), could identify cancer. Of the 102 men, 43 were diagnosed with significant prostate cancer. The PI-RADS v2.1 test was helpful in spotting cancer in the outer part of the prostate but not as reliable in detecting it in the inner part. PSA and the free/total PSA ratio also showed good accuracy in diagnosing cancer. Overall, PI-RADS v2.1 is useful for finding prostate cancer in certain areas, but more research is needed to improve detection in other parts of the prostate.

Evaluation of the Simple Summary

**Assessment Category**	**Score**	**Assessment Category**	**Score**	**Assessment Category**	**Score**
Comprehension Score	3	Gunning Fog Score	9.4	Words	148
Accuracy Score	0	SMOG Index	6.9	Complex words	15
Reading Age (years)	13.5	Coleman Liau Index	9.5	Percent of complex words	10.1
Flesch Kincaid Reading Ease	68.0	Automated Readability Index	5.5	Average words per sentence	13.5
Flesch Kincaid Grade Level	7.1	Sentences	11	Average syllables per word	1.5

Manuscript 5

Richter C, Mezger E, Schüffler PJ, Sommer W, Fusco F, Hauner K, Schmid SC, Gschwend JE, Weichert W, Schwamborn K, Pförringer D, Schlitter AM. Pathological Reporting of Radical Prostatectomy Specimens Following ICCR Recommendation: Impact of Electronic Reporting Tool Implementation on Quality and Interdisciplinary Communication in a Large University Hospital. Curr Oncol. 2022 Sep 30;29(10):7245-7256. doi: 10.3390/curroncol29100571. PMID: 36290848; PMCID: PMC9600383.

Abstract

Prostate cancer represents one of the most common malignant tumors in male patients in Germany. The pathological reporting of radical prostatectomy specimens following a structured process constitutes an excellent prototype for the introduction of software-based standardized structured reporting in pathology. This can lead to reports of higher quality and could create a fundamental improvement for future AI applications. A software-based reporting template was used to generate standardized structured pathological reports of radical prostatectomy specimens of patients treated at the University Hospital Klinikum rechts der Isar of Technische Universität München, Germany. Narrative reports (NR) and standardized structured reports (SSR) were analyzed with regard to completeness, and clinicians' satisfaction with each report type was evaluated. SSR show considerably higher completeness than NR. A total of 10 categories out of 32 were significantly more complete in SSR than in NR ($p < 0.05$). Clinicians awarded overall high scores in NR and SSR reports. One rater acknowledged a significantly higher level of clarity and time saving when comparing SSR to NR. Our findings highlight that the standardized structured reporting of radical prostatectomy specimens, qualifying as level 5 reports, significantly increases objectively measured content quality and the level of completeness. The implementation of nationwide SSR in Germany, particularly in oncologic pathology, can serve pathologists, clinicians, and patients.

Keywords: pathological reporting; prostate cancer; quality improvement; structured reporting templates.

Lay Summary (simple Prompt)

This study examined the impact of using a standardized, software-based reporting tool for documenting the findings from prostate cancer surgeries at a large university hospital in Germany. The researchers aimed to see if this structured reporting method would improve the completeness and quality of pathology reports compared to traditional narrative reports. They found that the structured reports were significantly more complete in 10 out of 32 key categories. Clinicians also found these structured reports clearer and more time-efficient. The findings suggest that adopting standardized, structured reporting across Germany could enhance the quality of pathology reports, improve communication between doctors, and potentially benefit patient care by providing more accurate and comprehensive information.

Evaluation of the Simple Summary

**Assessment Category**	**Score**	**Assessment Category**	**Score**	**Assessment Category**	**Score**
Comprehension Score	5	Gunning Fog Score	19.7	Words	113
Accuracy Score	1	SMOG Index	14.6	Complex words	32
Reading Age (years)	23.5	Coleman Liau Index	19.9	Percent of complex words	28.3
Flesch Kincaid Reading Ease	16.9	Automated Readability Index	18.5	Average words per sentence	22.6
Flesch Kincaid Grade Level	16.5	Sentences	5	Average syllables per word	2.0

Lay Summary (extended Prompt)

This study looked at how using a structured, software-based tool for reporting prostate cancer pathology can improve the quality and clarity of reports. Prostate cancer is common in men, and accurate pathology reports after surgery are important for treatment decisions. Researchers compared traditional narrative reports (NR) with standardized structured reports (SSR) for patients who had prostate surgery at a large university hospital in Germany. The findings showed that SSRs were more complete and detailed in 10 out of 32 categories. Clinicians also found SSRs clearer and more time-saving. These results suggest that using standardized reporting tools can improve the quality of pathology reports and make communication between doctors easier, benefiting both clinicians and patients. Implementing such tools nationwide could improve cancer care in Germany.

Evaluation of the Simple Summary

**Assessment Category**	**Score**	**Assessment Category**	**Score**	**Assessment Category**	**Score**
Comprehension Score	5	Gunning Fog Score	16.1	Words	126
Accuracy Score	0	SMOG Index	12.0	Complex words	30
Reading Age (years)	20.5	Coleman Liau Index	17.9	Percent of complex words	23.8
Flesch Kincaid Reading Ease	30.8	Automated Readability Index	14.6	Average words per sentence	18.0
Flesch Kincaid Grade Level	13.4	Sentences	7	Average syllables per word	1.9

Manuscript 6

Paladini A, Cochetti G, Colau A, Mouton M, Ciarletti S, Felici G, Maiolino G, Balzarini F, Sèbe P, Mearini E. The Challenges of Patient Selection for Prostate Cancer Focal Therapy: A Retrospective Observational Multicentre Study. Curr Oncol. 2022 Sep 22;29(10):6826-6833. doi: 10.3390/curroncol29100538. PMID: 36290815; PMCID: PMC9600719.

Abstract

Increased diagnoses of silent prostate cancer (PCa) have led to overtreatment and consequent functional side effects. Focal therapy (FT) applies energy to a prostatic index lesion treating only the clinically significant PCa focus. We analysed the potential predictive factors of FT failure. We collected data from patients who underwent robot-assisted radical prostatectomy (RARP) in two high-volume hospitals from January 2017 to January 2020. The inclusion criteria were: one MRI-detected lesion with a Gleason Score (GS) of ≤7, ≤cT2a, PSA of ≤10 ng/mL, and GS 6 on a random biopsy with ≤2 positive foci out of 12. Potential oncological safety of FT was defined as the respect of clinicopathological inclusion criteria on histology specimens, no extracapsular extension, and no biochemical, local, or metastatic recurrence within 12 months. To predict FT failure, we performed uni- and multivariate logistic regression. Sixty-seven patients were enrolled. The MRI index lesion median size was 11 mm; target lesions were ISUP grade 1 in 27 patients and ISUP grade 2 in 40. Potential FT failure occurred in 32 patients, and only the PSA value resulted as a predictive parameter ($p < 0.05$). The main issue for FT is patient selection, mainly because of multifocal csPCa foci. Nevertheless, FT could represent a therapeutic alternative for highly selected low-risk PCa patients.

Keywords: MRI; PSA; focal therapy; low-risk PCa; prostate cancer.theranostics

Lay Summary (simple Prompt)

This study explores the challenges in selecting patients for focal therapy (FT) for prostate cancer, a treatment that targets only the cancerous part of the prostate to reduce side effects. Researchers reviewed data from patients who underwent prostate surgery to identify factors that might predict FT failure. They found that the main challenge in using FT is selecting the right patients, especially because prostate cancer often has multiple significant areas. The study showed that only the prostate-specific antigen (PSA) level could help predict if FT might not be effective. Despite these challenges, FT could still be a good option for carefully chosen patients with low-risk prostate cancer, potentially offering a less invasive alternative to more extensive treatments.

Evaluation of the Simple Summary

**Assessment Category**	**Score**	**Assessment Category**	**Score**	**Assessment Category**	**Score**
Comprehension Score	5	Gunning Fog Score	16.2	Words	119
Accuracy Score	1	SMOG Index	11.6	Complex words	20
Reading Age (years)	20.5	Coleman Liau Index	16.3	Percent of complex words	16.8
Flesch Kincaid Reading Ease	37.6	Automated Readability Index	16.2	Average words per sentence	23.8
Flesch Kincaid Grade Level	13.9	Sentences	5	Average syllables per word	1.7

Lay Summary (extended Prompt)

This study looked at selecting the right patients for focal therapy (FT) to treat prostate cancer. FT targets only the main tumor in the prostate, aiming to reduce side effects of overtreatment. The researchers analyzed data from patients who had prostate surgery to see which factors predicted treatment failure. They included patients with low-risk prostate cancer based on MRI, PSA levels, and biopsy results. Out of 67 patients studied, 32 had signs that FT might not have worked, with PSA levels being the main factor linked to failure. The study highlights that choosing the right patients for FT is challenging due to the possibility of undetected cancer in other parts of the prostate. However, FT could still be a good option for some low-risk patients.

Evaluation of the Simple Summary

**Assessment Category**	**Score**	**Assessment Category**	**Score**	**Assessment Category**	**Score**
Comprehension Score	5	Gunning Fog Score	11.4	Words	127
Accuracy Score	0	SMOG Index	8.0	Complex words	13
Reading Age (years)	15.5	Coleman Liau Index	12.8	Percent of complex words	10.2
Flesch Kincaid Reading Ease	59.9	Automated Readability Index	10.5	Average words per sentence	18.1
Flesch Kincaid Grade Level	9.4	Sentences	7	Average syllables per word	1.5

Manuscript 7

Banna GL, Basso U, Giunta EF, Fratino L, Rebuzzi SE, Buti S, Maruzzo M, De Giorgi U, Murianni V, Cinausero M, Lipari H, Gamba T, Caffo O, Bimbatti D, Dri A, Mosca A, Ermacora P, Vignani F, Msaki A, Bonifacio B, Lombardo V, Conteduca V, Fornarini G, Rescigno P. The Geriatric G8 Score Is Associated with Survival Outcomes in Older Patients with Advanced Prostate Cancer in the ADHERE Prospective Study of the Meet-URO Network. Curr Oncol. 2022 Oct 14;29(10):7745-7753. doi: 10.3390/curroncol29100612. PMID: 36290889; PMCID: PMC9600362.

Abstract

Introduction: Androgen receptor pathway inhibitors (ARPIs) have been increasingly offered to older patients with prostate cancer (PC). However, prognostic factors relevant to their outcome with ARPIs are still little investigated. **Methods and Materials:** The Meet-URO network ADHERE was a prospective multicentre observational cohort study evaluating and monitoring adherence to ARPIs metastatic castrate-resistant PC (mCRPC) patients aged ≥70. Cox regression univariable and multivariable analyses for radiographic progression-free (rPFS) and overall survival (OS) were performed. Unsupervised median values and literature-based thresholds where available were used as cut-offs for quantitative variables. **Results:** Overall, 234 patients were enrolled with a median age of 78 years (73-82); 86 were treated with abiraterone (ABI) and 148 with enzalutamide (ENZ). With a median follow-up of 15.4 months (mo.), the median rPFS was 26.0 mo. (95% CI, 22.8-29.3) and OS 48.8 mo. (95% CI, 36.8-60.8). At the MVA, independent prognostic factors for both worse rPFS and OS were Geriatric G8 assessment ≤ 14 ($p < 0.001$ and $p = 0.004$) and PSA decline ≥50% ($p < 0.001$ for both); time to castration resistance ≥ 31 mo. and setting of treatment (i.e., post-ABI/ENZ) for rPFS only ($p < 0.001$ and $p = 0.01$, respectively); age ≥78 years for OS only ($p = 0.008$). **Conclusions:** Baseline G8 screening is recommended for mCRPC patients aged ≥70 to optimise ARPIs in vulnerable individuals, including early introduction of palliative care.

Keywords: G8 score; adherence to treatment; geriatric assessment; prostate cancer.

Lay Summary (simple Prompt)

This study examined how the G8 score, a tool used to assess the health of older adults, can predict survival outcomes in patients aged 70 and older with advanced prostate cancer who are being treated with specific hormone therapies (abiraterone or enzalutamide). The research followed 234 patients and found that a lower G8 score (indicating poorer health) and less than a 50% decline in PSA levels were linked to shorter survival times. Additionally, patients who developed resistance to hormone therapy after more than 31 months had better outcomes. The findings suggest that using the G8 score at the start of treatment can help doctors identify more vulnerable patients who might benefit from earlier supportive care, potentially improving their quality of life and survival outcomes.

Evaluation of the Simple Summary

**Assessment Category**	**Score**	**Assessment Category**	**Score**	**Assessment Category**	**Score**
Comprehension Score	5	Gunning Fog Score	19.2	Words	124
Accuracy Score	0	SMOG Index	13.2	Complex words	21
Reading Age (years)	21.5	Coleman Liau Index	14.6	Percent of complex words	16.9
Flesch Kincaid Reading Ease	32.8	Automated Readability Index	18.4	Average words per sentence	31.0
Flesch Kincaid Grade Level	16.4	Sentences	4	Average syllables per word	1.7

Lay Summary (extended Prompt)

This study explored how well older patients with advanced prostate cancer respond to certain medications, specifically androgen receptor pathway inhibitors (ARPIs) like abiraterone and enzalutamide. Researchers focused on patients aged 70 and older to see if the G8 geriatric assessment score could predict survival outcomes. The study followed 234 patients over about 15 months and found that those with lower G8 scores (indicating greater frailty) and a lower PSA decline had worse outcomes. Additionally, age and the length of time patients had been resistant to hormone therapy also influenced survival. The findings suggest that the G8 score is a valuable tool for identifying patients who may benefit from more personalized care, including earlier palliative care, to improve their treatment outcomes.

Evaluation of the Simple Summary

**Assessment Category**	**Score**	**Assessment Category**	**Score**	**Assessment Category**	**Score**
Comprehension Score	5	Gunning Fog Score	17.3	Words	120
Accuracy Score	1	SMOG Index	12.4	Complex words	23
Reading Age (years)	21.5	Coleman Liau Index	16.8	Percent of complex words	19.2
Flesch Kincaid Reading Ease	28.8	Automated Readability Index	16.7	Average words per sentence	24.0
Flesch Kincaid Grade Level	15.2	Sentences	5	Average syllables per word	1.8

Manuscript 8

Cicchetti A, Noale M, Dordoni P, Noris Chiorda B, De Luca L, Bellardita L, Montironi R, Bertoni F, Bassi P, Schiavina R, Gacci M, Serni S, Sessa F, Maruzzo M, Maggi S, Valdagni R; Pros-IT CNR Study Group. Patient-Factors Influencing the 2-Year Trajectory of Mental and Physical Health in Prostate Cancer Patients. Curr Oncol. 2022 Oct 31;29(11):8244-8260. doi: 10.3390/curroncol29110651. PMID: 36354711; PMCID: PMC9689299.

Abstract

This study aimed to examine the physical and mental Quality of Life (QoL) trajectories in prostate cancer (PCa) patients participating in the Pros-IT CNR study. QoL was assessed using the Physical (PCS) and Mental Component Score (MCS) of Short-Form Health Survey upon diagnosis and two years later. Growth mixture models were applied on 1158 patients and 3 trajectories over time were identified for MCS: 75% of patients had constantly high scores, 13% had permanently low scores and 12% starting with low scores had a recovery; the predictors that differentiated the trajectories were age, comorbidities, a family history of PCa, and the bowel, urinary and sexual functional scores at diagnosis. In the physical domain, 2 trajectories were defined: 85% of patients had constantly high scores, while 15% started with low scores and had a further slight decrease. Two years after diagnosis, the psychological and physical status was moderately compromised in more than 10% of PCa patients. For mental health, the trajectory analysis suggested that following the compromised patients at diagnosis until treatment could allow identification of those more vulnerable, for which a level 2 intervention with support from a non-oncology team supervised by a clinical psychologist could be of help.

Keywords: SF-12; growth mixture model; health related quality of life; prostate cancer.

Lay Summary (simple Prompt)

This study investigated how prostate cancer affects the mental and physical health of patients over two years. By analyzing data from 1,158 patients, researchers identified different patterns in quality of life: most patients maintained good mental and physical health, but some experienced persistent or worsening problems. Factors such as age, other health conditions, family history of prostate cancer, and bowel, urinary, and sexual function at diagnosis influenced these patterns. About 15% of patients had declining physical health, while over 10% had significant mental health challenges two years after diagnosis. The study suggests that early identification and targeted support for patients who show signs of compromised mental or physical health could improve their overall well-being, potentially involving non-oncology specialists under the guidance of a clinical psychologist to provide additional support.

Evaluation of the Simple Summary

**Assessment Category**	**Score**	**Assessment Category**	**Score**	**Assessment Category**	**Score**
Comprehension Score	5	Gunning Fog Score	20.3	Words	132
Accuracy Score	1	SMOG Index	14.6	Complex words	32
Reading Age (years)	23.5	Coleman Liau Index	18.3	Percent of complex words	24.2
Flesch Kincaid Reading Ease	19.2	Automated Readability Index	19.0	Average words per sentence	26.4
Flesch Kincaid Grade Level	17.1	Sentences	5	Average syllables per word	1.9

Lay Summary (extended Prompt)

This study examined the mental and physical health of prostate cancer patients over two years to understand how their quality of life changes after diagnosis. Researchers followed 1,158 patients and identified three mental health patterns: 75% had consistently good mental health, 13% had persistently low mental health, and 12% improved over time. Age, other health conditions, family history of prostate cancer, and initial bowel, urinary, and sexual function were linked to these patterns. For physical health, 85% of patients maintained good physical scores, while 15% started with lower physical health, which slightly declined further. The findings suggest that early identification of patients at risk for mental health struggles could lead to more targeted support, potentially involving a clinical psychologist to improve their quality of life.

Evaluation of the Simple Summary

**Assessment Category**	**Score**	**Assessment Category**	**Score**	**Assessment Category**	**Score**
Comprehension Score	5	Gunning Fog Score	18.0	Words	126
Accuracy Score	0	SMOG Index	12.9	Complex words	25
Reading Age (years)	21.5	Coleman Liau Index	16.5	Percent of complex words	19.8
Flesch Kincaid Reading Ease	32.2	Automated Readability Index	17.0	Average words per sentence	25.2
Flesch Kincaid Grade Level	15.0	Sentences	5	Average syllables per word	1.8

Manuscript 9

Hu X, Miao J, Huang J, Qian L, Zhang D, Wei H. Influence of Active Surveillance on Gleason Score Upgrade and Prognosis in Low- and Favorable Intermediate-Risk Prostate Cancer. Curr Oncol. 2022 Oct 21;29(10):7964-7978. doi: 10.3390/curroncol29100630. PMID: 36290907; PMCID: PMC9600547.

Abstract

Few studies have focused on the link between active surveillance (AS) and Gleason score upgrade (GSU) and its impact on the prognosis of patients with prostate cancer (PCa). This study aimed to analyze the effect of AS duration on GSU and prognostic value based on risk stratification. All eligible patients were risk-stratified according to AUA guidelines into low-risk (LR), favorable intermediate-risk (FIR), and unfavorable intermediate-risk (UIR) PCa. Within the Surveillance, Epidemiology, and End Results Program (SEER) database, 28,368 LR, 27,243 FIR, and 12,210 UIR PCa patients were included. The relationship between AS duration and GSU was identified with univariate and multivariate logistic regression. Discrimination according to risk stratification of AS duration and GSU was tested by Kaplan-Meier analysis and competing risk regression models. The proportion of patients who chose AS was the highest among LR PCa (3434, 12.1%), while the proportion in UIR PCa was the lowest (887, 7.3%). The AS duration was only associated with GSU in LR PCa, with a high Gleason score (GS) at diagnosis being a strong predictor of GSU for FIR and UIR PCa. Kaplan-Meier analysis indicated that long-term surveillance only made a significant difference in prognosis in UIR PCa. The competing risk analysis indicated that once GS was upgraded to 8 or above, the prognosis in each group was significantly worse. AS is recommended for LR and FIR PCa until GS is upgraded to 8, but AS may not be suitable for some UIR PCa patients.

Keywords: Gleason score upgrade; active surveillance; favorable intermediate-risk prostate cancer; low-risk prostate cancer; risk stratification.

Lay Summary (simple Prompt)

This study examined how active surveillance (regular monitoring instead of immediate treatment) affects the Gleason score (a key marker of prostate cancer severity) and the prognosis of prostate cancer patients. We looked at different risk groups: low-risk, favourable intermediate-risk, and unfavourable intermediate-risk. Our findings showed that in low-risk patients, longer surveillance did not lead to an increase in Gleason score. However, for patients whose Gleason score rose to 8 or above, the prognosis worsened. Active surveillance is recommended for patients with low-risk and favourable intermediate-risk prostate cancer, as long as their Gleason score does not worsen. For some patients with unfavourable intermediate-risk prostate cancer, surveillance may not be as beneficial.

Evaluation of the Simple Summary

Assessment Category	Score	Assessment Category	Score	Assessment Category	Score
Comprehension Score	5	Gunning Fog Score	15.7	Words	117
Accuracy Score	1	SMOG Index	11.3	Complex words	23
Reading Age (years)	19.5	Coleman Liau Index	17.3	Percent of complex words	19.7
Flesch Kincaid Reading Ease	34.5	Automated Readability Index	14.8	Average words per sentence	19.5
Flesch Kincaid Grade Level	13.3	Sentences	6	Average syllables per word	1.8

<u>Lay Summary (extended Prompt)</u>

This study looked at how regular monitoring, called active surveillance, affects the Gleason score (which measures how aggressive prostate cancer is) and what it means for a patient's outlook. We focused on men with low-risk and intermediate-risk prostate cancer. The patients were divided into three groups: low-risk, favourable intermediate-risk, and unfavourable intermediate-risk. We found that patients with low-risk cancer could safely be monitored for a longer time without their Gleason score getting worse. However, when the Gleason score increased to 8 or higher, the chances of a poor outcome rose in all groups. For men with low-risk or favourable intermediate-risk cancer, active surveillance is a good option as long as their Gleason score stays below 8. But for those with unfavourable intermediate-risk cancer, regular monitoring might not be the best choice.

<u>Evaluation of the Simple Summary</u>

**Assessment Category**	**Score**	**Assessment Category**	**Score**	**Assessment Category**	**Score**
Comprehension Score	5	Gunning Fog Score	13.4	Words	140
Accuracy Score	0	SMOG Index	9.6	Complex words	19
Reading Age (years)	17.5	Coleman Liau Index	14.3	Percent of complex words	13.6
Flesch Kincaid Reading Ease	50.0	Automated Readability Index	12.6	Average words per sentence	20.0
Flesch Kincaid Grade Level	11.3	Sentences	7	Average syllables per word	1.7

Manuscript 10

Hu J, Aprikian AG, Saleh RR, Dragomir A. Utilization Trends of Novel Hormonal Agents in Metastatic Castration-Resistant Prostate Cancer in Quebec. Curr Oncol. 2022 Nov 12;29(11):8626-8637. doi: 10.3390/curroncol29110680. PMID: 36421333; PMCID: PMC9688923.

Abstract

Background: The introduction of novel hormonal agents (NHAs) such as abiraterone acetate (ABI) and enzalutamide (ENZ) for metastatic castration-resistant prostate cancer (mCRPC) was an important milestone given their survival benefits, tolerability, and ease of administration relative to taxane chemotherapies. This descriptive study sought to describe the utilization trends of ABI and ENZ in patients with mCRPC in the early years after their approval in the province of Quebec in Canada.

Methods: A retrospective population-based cohort was extracted from Quebec public healthcare administrative databases. The cohort included first-time users of NHAs (ABI or ENZ) from 2011 to 2016. The primary analyses aimed to describe the overall temporal trends (2011-2016) of NHA initiators by chemotherapy status (chemotherapy-naïve versus post-chemotherapy), and prescribing specialty (medical oncology versus urology versus others).

Results: The cohort comprised 2183 patients, with 1562 (72%) in the chemotherapy-naïve group and 621 (28%) in the post-chemotherapy group. While the majority of patients were post-chemotherapy NHA initiators in 2012, this proportion decreased over time and accounted for only 13% of NHA initiators by the end of 2016. Medical oncologists were the most frequent prescribers of NHAs (upwards of 60%) throughout 2012 but fell to 45% by the end of 2016. Conversely, the proportion of prescriptions by urologists increased from 22% in 2012 to 42% in 2016.

Conclusion: Over time, there was an increasing proportion of (1) patients who initiated NHAs without prior chemotherapy treatment, (2) NHA prescribing by urologists, and (3) ENZ users. Taken together, this implies that the introduction of NHAs has altered the management of mCRPC and urologists quickly adopted NHAs into their practice.

Keywords: abiraterone; drug utilization; enzalutamide; prostate cancer.

Lay Summary (simple Prompt)

This study examined how new hormonal treatments, abiraterone acetate (ABI) and enzalutamide (ENZ), were used in managing metastatic castration-resistant prostate cancer (mCRPC) in Quebec, Canada, from 2011 to 2016. These treatments offer benefits in survival and ease of use compared to traditional chemotherapy. The study found that over time, more patients started using these new hormonal agents without prior chemotherapy, and there was a significant increase in prescriptions by urologists rather than oncologists. The use of ENZ also became more common. These trends suggest that these hormonal agents have become a key part of prostate cancer treatment, and urologists have rapidly integrated them into their practices, changing how mCRPC is managed.

Evaluation of the Simple Summary

**Assessment Category**	**Score**	**Assessment Category**	**Score**	**Assessment Category**	**Score**
Comprehension Score	4	Gunning Fog Score	17.2	Words	112
Accuracy Score	1	SMOG Index	12.7	Complex words	24
Reading Age (years)	20.5	Coleman Liau Index	16.7	Percent of complex words	21.4
Flesch Kincaid Reading Ease	29.3	Automated Readability Index	15.8	Average words per sentence	22.4
Flesch Kincaid Grade Level	14.7	Sentences	5	Average syllables per word	1.8

Lay Summary (extended Prompt)

This study looked at how new hormonal treatments, abiraterone acetate (ABI) and enzalutamide (ENZ), were used in men with metastatic castration-resistant prostate cancer (mCRPC) in Quebec from 2011 to 2016. These treatments offer important benefits, such as improving survival and being easier to tolerate compared to chemotherapy. Researchers analyzed data to see how often these medications were prescribed and by whom. They found that more patients started using these treatments without having chemotherapy first, and urologists became more involved in prescribing them over time. By 2016, 42% of prescriptions were written by urologists, up from 22% in 2012. The study shows how quickly these hormonal therapies became a key part of treating prostate cancer, especially for patients who hadn't received chemotherapy.

Evaluation of the Simple Summary

**Assessment Category**	**Score**	**Assessment Category**	**Score**	**Assessment Category**	**Score**
Comprehension Score	5	Gunning Fog Score	16.7	Words	122
Accuracy Score	0	SMOG Index	12.0	Complex words	26
Reading Age (years)	20.5	Coleman Liau Index	16.0	Percent of complex words	21.3
Flesch Kincaid Reading Ease	32.9	Automated Readability Index	14.1	Average words per sentence	20.3
Flesch Kincaid Grade Level	13.7	Sentences	6	Average syllables per word	1.8

Manuscript 11

Pandit AA, Gressler LE, Halpern MT, Kamel M, Payakachat N, Li C. Racial/Ethnic Disparities in Patient Care Experiences among Prostate Cancer Survivors: A SEER-CAHPS Study. Curr Oncol. 2022 Nov 1;29(11):8357-8373. doi: 10.3390/curroncol29110659. PMID: 36354719; PMCID: PMC9689524.

Abstract

Purpose: To evaluate racial/ethnic disparities in patient care experiences (PCEs) among prostate cancer (PCa) survivors.

Methods: This retrospective study used 2007-2015 National Cancer Institute Surveillance, Epidemiology and End Results registry data linked to Consumer Assessment of Healthcare Providers and Systems surveys. First survey ≥ 6 months post-PCa diagnosis was analyzed. We performed multivariable linear regression, adjusting for demographic and clinical covariates, to evaluate the association of race/ethnicity (non-Hispanic Whites (NHWs), non-Hispanic Black (NHBs), Hispanic, non-Hispanic Asian (NHAs), and other races) with PCE composite measures: getting needed care, doctor communication, getting care quickly, getting needed prescription drugs (Rx), and customer service.

Results: Among 7319 PCa survivors, compared to NHWs, Hispanics, NHBs and NHAs reported lower scores for getting care quickly (ß = -3.69; $p = 0.002$, ß = -2.44; $p = 0.021$, and ß = -6.44; $p < 0.001$, respectively); Hispanics scored worse on getting needed care (ß = -2.16; $p = 0.042$) and getting needed Rx (ß = -2.93; $p = 0.009$), and NHAs scored worse on customer service (ß = -7.60; $p = 0.003$), and getting needed Rx (ß = -3.08; $p = 0.020$). However, NHBs scored better than NHWs on doctor communication (ß = 1.95, $p = 0.006$). No statistically significant differences were found between other races and NHWs.

Conclusions: Comparing to NHWs, Hispanics and NHAs reported worse experiences on several PCE composite measures, while NHBs reported worse scores on one but better scores on another PCE composite measure. Further research is needed to understand the reasons behind these disparities and their influence on healthcare utilization and health outcomes among PCa survivors.

Keywords: CAHPS; SEER; disparity; patient care experiences; prostate cancer.

Lay Summary (simple Prompt)

This study investigates differences in patient care experiences among prostate cancer survivors from different racial and ethnic backgrounds. By analyzing survey data linked to cancer registry information, the researchers found that Hispanic, non-Hispanic Black (NHB), and non-Hispanic Asian (NHA) survivors often reported worse experiences compared to non-Hispanic White (NHW) survivors in areas such as getting care quickly, obtaining necessary treatments, and customer service. Interestingly, NHB survivors reported better experiences with doctor communication than NHWs. These findings highlight ongoing disparities in healthcare experiences among prostate cancer survivors from diverse racial and ethnic groups. Understanding these differences is crucial for improving healthcare delivery and ensuring equitable treatment outcomes for all patients, regardless of their racial or ethnic background. Further research is needed to explore the causes of these disparities and how they affect the overall health and well-being of survivors.

Evaluation of the Simple Summary

**Assessment Category**	**Score**	**Assessment Category**	**Score**	**Assessment Category**	**Score**
Comprehension Score	5	Gunning Fog Score	19.3	Words	142
Accuracy Score	1	SMOG Index	14.9	Complex words	40
Reading Age (years)	23.5	Coleman Liau Index	20.7	Percent of complex words	28.2
Flesch Kincaid Reading Ease	17.8	Automated Readability Index	19.6	Average words per sentence	23.7
Flesch Kincaid Grade Level	16.7	Sentences	6	Average syllables per word	2.0

Lay Summary (extended Prompt)

This study looked at differences in how prostate cancer survivors from various racial and ethnic groups experience healthcare. Using data from a large national cancer registry, the researchers analyzed patient care experiences, including getting needed care, doctor communication, getting care quickly, and other aspects of service. The study found that Hispanic, Black, and Asian prostate cancer survivors often reported worse experiences in getting care quickly compared to White survivors. Hispanics also had more difficulty getting needed care and prescription drugs, while Asians reported worse customer service and access to prescription drugs. However, Black survivors rated doctor communication more positively than White survivors. The study highlights that racial and ethnic disparities exist in healthcare experiences, and more research is needed to understand why these differences occur and how they affect patient outcomes.

Evaluation of the Simple Summary

**Assessment Category**	**Score**	**Assessment Category**	**Score**	**Assessment Category**	**Score**
Comprehension Score	5	Gunning Fog Score	17.9	Words	131
Accuracy Score	1	SMOG Index	13.5	Complex words	33
Reading Age (years)	25.8	Coleman Liau Index	19.8	Percent of complex words	25.2
Flesch Kincaid Reading Ease	22.5	Automated Readability Index	17.9	Average words per sentence	21.8
Flesch Kincaid Grade Level	15.1	Sentences	6	Average syllables per word	1.9

Manuscript 12

Sun G, Liang Z, Jiang Y, Ma S, Chen S, Liu R. Clinical Analysis of Perioperative Outcomes on Neoadjuvant Hormone Therapy before Laparoscopic and Robot-Assisted Surgery for Localized High-Risk Prostate Cancer in a Chinese Cohort. Curr Oncol. 2022 Nov 14;29(11):8668-8676. doi: 10.3390/curroncol29110683. PMID: 36421336; PMCID: PMC9689847.

Abstract

Objective: To analyze the perioperative outcomes of neoadjuvant hormone therapy (NHT) before laparoscopic and robot-assisted surgery for localized high-risk prostate cancer in a Chinese cohort.

Methods: The clinical data of 385 patients with localized high-risk prostate cancer who underwent radical prostatectomy (RP) in our hospital from January 2019 to June 2021 were analyzed retrospectively, including 168 patients with preoperative NHT and 217 patients with simple surgery. Clinical characteristics were compared in the above two groups, the laparoscopic RP (LRP) cohort (n = 234) and the robot-assisted laparoscopic radical prostatectomy (RALP) cohort (n = 151), respectively.

Results: In the overall cohort, compared with the control group, the NHT group had a shorter operative time, less blood loss, a lower positive surgical margin rate, and a higher proportion of Gleason score (GS) downgrading after the operation ($p < 0.05$). However, there was no significant difference in hospitalization time, biochemical recurrence, urine leakage, urinary continence, or prostate-specific antigen (PSA) progression-free survival ($p > 0.05$). In the LRP cohort, it was found that the NHT group also had shorter operative time, less blood loss, lower positive surgical margin rate, a higher proportion of GS downgrading after the operation, and faster recovery of urinary control than the control group ($p < 0.05$). There was no marked difference in hospitalization time, biochemical recurrence, urinary leakage, or PSA progression-free survival. However, in the RALP cohort, the NHT group had a significant difference in the GS downgrading after the operation compared with the control group ($p < 0.05$). In the overall cohort, multiple analyses showed that initial PSA level, GS at biopsy, clinical T stage, lymph node invasion, use of NHT, and surgical methods were significantly associated with positive surgical margin ($p < 0.05$) while NHT did not account for biochemical recurrence ($p > 0.05$).

Conclusions: NHT can lower the difficulty of surgery, reduce positive surgical margin rate, and help recovery in short-term urinary control in patients with high-risk prostate cancer after LRP. However, we do not have evidence on the benefit of NHT in high-risk PCa patients treated with RALP. For these patients, surgery can be performed as early as possible.

Keywords: laparoscopic radical prostatectomy; neoadjuvant hormone therapy; prostate cancer; robot-assisted radical prostatectomy.

Lay Summary (simple Prompt)

This study examines the effects of using hormone therapy before surgery in patients with high-risk prostate cancer. The research compared patients who received hormone therapy before their operation with those who did not, focusing on outcomes after laparoscopic and robot-assisted prostate surgeries. The findings suggest that hormone therapy before laparoscopic surgery can make the procedure easier, reduce blood loss, lower the chances of cancer cells remaining at the edges of removed tissue, and improve short-term urinary control after surgery. However, hormone therapy did not show a clear benefit in reducing the recurrence of cancer or in outcomes for patients undergoing robot-assisted surgery. The results provide important insights into optimizing treatment strategies for high-risk prostate cancer, suggesting that hormone therapy before surgery might be beneficial for some patients but not necessary for all.

Evaluation of the Simple Summary

**Assessment Category**	**Score**	**Assessment Category**	**Score**	**Assessment Category**	**Score**
Comprehension Score	5	Gunning Fog Score	20.6	Words	137
Accuracy Score	1	SMOG Index	14.8	Complex words	33
Reading Age (years)	23.5	Coleman Liau Index	17.6	Percent of complex words	24.1
Flesch Kincaid Reading Ease	20.9	Automated Readability Index	19.0	Average words per sentence	27.4
Flesch Kincaid Grade Level	17.1	Sentences	5	Average syllables per word	1.9

Lay Summary (extended Prompt)

This study looked at the effects of using hormone therapy before surgery (called neoadjuvant hormone therapy, or NHT) on patients with high-risk prostate cancer in China. Researchers compared 385 patients who had either laparoscopic or robot-assisted prostate surgery, with some receiving NHT before surgery. The study found that for those who had laparoscopic surgery, NHT made the operation easier, reduced blood loss, and improved short-term urinary control. It also helped lower the chance of cancer cells being left behind after surgery. However, for patients who had robot-assisted surgery, the only noticeable benefit of NHT was a downgrade in the aggressiveness of the cancer. NHT did not affect longer-term outcomes, like cancer recurrence, in either group. This suggests that NHT can be useful for some patients undergoing laparoscopic surgery, but more research is needed to confirm its benefits for those having robot-assisted surgery.

Evaluation of the Simple Summary

**Assessment Category**	**Score**	**Assessment Category**	**Score**	**Assessment Category**	**Score**
Comprehension Score	5	Gunning Fog Score	16.3	Words	148
Accuracy Score	0	SMOG Index	11.8	Complex words	29
Reading Age (years)	19.5	Coleman Liau Index	15.1	Percent of complex words	19.6
Flesch Kincaid Reading Ease	36.2	Automated Readability Index	13.9	Average words per sentence	21.1
Flesch Kincaid Grade Level	13.5	Sentences	7	Average syllables per word	1.8

Manuscript 13

Lucchini R, Franzese C, Vukcaj S, Purrello G, Panizza D, Faccenda V, Andreoli S, Poli GL, Baldaccini D, Lo Faro L, Tomatis S, Cazzaniga LF, Scorsetti M, Arcangeli S. Acute Toxicity and Quality of Life in a Post-Prostatectomy Ablative Radiation Therapy (POPART) Multicentric Trial. Curr Oncol. 2022 Nov 30;29(12):9349-9356. doi: 10.3390/curroncol29120733. PMID: 36547147; PMCID: PMC9776836.

Abstract

Background: The aim of this study was to investigate the feasibility of ultrahypofractionated radiotherapy to the prostate bed in patients with biochemical and/or clinical relapse following radical prostatectomy who were enrolled in the prospective, observational, multicentric POPART trial (NCT04831970). Methods: Patients with post-radical prostatectomy PSA levels of ≥0.1–2.0 ng/mL and/or local relapse at PSMA PET/CT or multiparametric MRI were treated with Linac-based SBRT on the prostate bed up to a total dose of 32.5 Gy in five fractions every other day (EQD21.5 = 74.2 Gy). Maximum acute toxicity was assessed using the Common Terminology Criteria for Adverse Events version 5 scale. International Consultation on Incontinence Questionnaire—Short Form (ICIQ-SF) and Prostate Cancer Index Composite for Clinical Practice (EPIC-CP) scores were assessed at baseline and during the follow-up. Results: From April 2021 to June 2022, thirty men with a median age of 72 years (range 55–82) were enrolled in three centers. The median PSA level before RT was 0.30 ng/mL (range 0.18–1.89 ng/mL). At 3 months post-treatment, no GI or ≥2 GU side effects were reported; three patients (10%) experienced Grade 1 GU toxicity. No changes in ICIQ-SF or in the urinary domains of EPIC-CP were observed, while a transient worsening was registered in the bowel domain. At the same time point, all but two patients, who progressed distantly, were found to be biochemically controlled with a median post-treatment PSA level of 0.07 ng/mL (range 0–0.48 ng/mL). Conclusions: Our preliminary findings show that SBRT can be safely extended to the postoperative setting, without an increase in short-term toxicity or a significant decline in QoL. Long-term results are needed to confirm this strategy.

Keywords:

prostate cancer; postoperative setting; SBRT

Lay Summary (simple Prompt)

This study explored the safety and effectiveness of a new, focused type of radiation therapy, called stereotactic body radiation therapy (SBRT), for men who showed signs of prostate cancer returning after surgery. The research involved 30 patients who received SBRT to the area where the prostate used to be, using a high dose of radiation given in five sessions. The study monitored side effects and quality of life before and after treatment. Results showed that most patients tolerated the treatment well, with only mild urinary side effects and a temporary increase in bowel symptoms. Importantly, most patients had good control of their cancer without significant negative effects on their quality of life. These findings suggest that SBRT may be a safe and effective option for treating prostate cancer recurrence after surgery, although more research is needed to confirm these results over the long term.

Evaluation of the Lay Summary

**Assessment Category**	**Score**	**Assessment Category**	**Score**	**Assessment Category**	**Score**
Comprehension Score	5	Gunning Fog Score	15.7	Words	144
Accuracy Score	0	SMOG Index	11.1	Complex words	22
Reading Age (years)	19.5	Coleman Liau Index	14.9	Percent of complex words	15.3
Flesch Kincaid Reading Ease	38.0	Automated Readability Index	15.2	Average words per sentence	24.0
Flesch Kincaid Grade Level	13.9	Sentences	6	Average syllables per word	1.7

Lay Summary (extended Prompt)

This study aimed to assess the safety and impact of using ultrahypofractionated radiotherapy, a focused form of radiation, to treat prostate cancer patients who had biochemical or clinical relapse after surgery. Thirty men with rising PSA levels or signs of local cancer recurrence were treated with five sessions of radiation therapy over two weeks. Researchers monitored the patients for side effects and changes in quality of life. After three months, no serious gastrointestinal or urinary side effects were reported, and only three patients experienced mild urinary symptoms. The patients' quality of life remained stable, with only a temporary change in bowel function. Most patients showed a significant drop in PSA levels, indicating good control of the cancer. These early findings suggest that this radiation approach is safe and effective in the short term, though long-term results are still needed.

Evaluation of the Lay Summary

Assessment Category	Score	Assessment Category	Score	Assessment Category	Score
Comprehension Score	5	Gunning Fog Score	15.1	Words	140
Accuracy Score	0	SMOG Index	11.0	Complex words	25
Reading Age (years)	19.5	Coleman Liau Index	16.0	Percent of complex words	17.9
Flesch Kincaid Reading Ease	35.5	Automated Readability Index	14.0	Average words per sentence	20.0
Flesch Kincaid Grade Level	13.3	Sentences	7	Average syllables per word	1.8

Manuscript 14

Fiorica F, Buttigliero C, Grigolato D, Muraro M, Turco F, Munoz F, Tucci M. Addition of New Androgen Receptor Pathway Inhibitors to Docetaxel and Androgen Deprivation Therapy in Metastatic Hormone-Sensitive Prostate Cancer: A Systematic Review and Metanalysis. Curr Oncol. 2022 Dec 4;29(12):9511-9524. doi: 10.3390/curroncol29120747. PMID: 36547161; PMCID: PMC9776703.

Abstract

In recent years, significant changes have occurred in metastatic hormone-sensitive prostate cancer (mHSPC) management, where docetaxel and new androgen receptor pathway inhibitors (ARPI) have been shown to improve overall survival (OS) compared to androgen deprivation therapy (ADT). Recent data could once again radically change mHSPC treatment. PEACE-1 and ARASENS trials demonstrated a survival benefit of the addition of ARPI to docetaxel and ADT combination (triplet therapy), compared to docetaxel and ADT. With multiple options to choose from, it is crucial to identify the patients who would benefit most from triplet therapy. In this meta-analysis, we evaluated the activity of the triplet therapy versus docetaxel plus ADT in mHSPC. A systematic review of PubMed/Medline, Embase, and the proceedings of major international meetings was performed. Five RCTs fulfilled the inclusion criteria. PEACE-1 and ARASENS studies reported disease-free survival (DFS) and OS. Post hoc analysis of three other trials evaluated the combination of ARPI, docetaxel and ADT. Globally, 2538 patients were included (1270 triplet therapy; 1268 docetaxel + ADT). Triplet therapy was associated with improved OS (hazard ratio (HR) 0.74; 95% confidence interval (CI), 0.66-0.83, $p < 0.00001$). A statistically significant benefit was shown in high-volume mHSPC patients (HR 0.76; 95% CI 0.59-0.97, $p = 0.03$) and in patients with de novo metastatic disease (HR 0.73; 95% CI, 0.64-0.82, $p < 0.00001$). The addition of ARPI to standard therapy was associated with DFS improvement (HR 0.41; 95% CI, 0.35-0.49, $p < 0.00001$). This metanalysis shows a significant OS benefit from concomitant administration of ARPI, docetaxel and ADT in high volume and de novo mHSPC.

Keywords: de novo metastatic disease; high volume metastatic disease; hormone-sensitive prostate cancer; metanalysis; systematic review; triplet therapy.

Lay Summary (simple Prompt)

This study reviews recent evidence on the effectiveness of adding new drugs called androgen receptor pathway inhibitors (ARPI) to standard treatments for men with metastatic hormone-sensitive prostate cancer (mHSPC). The standard treatment typically includes androgen deprivation therapy (ADT) and chemotherapy with docetaxel. The review found that using a combination of ARPI, docetaxel, and ADT (known as triplet therapy) significantly improves overall survival and disease-free survival compared to using just docetaxel and ADT. The benefits were particularly notable in patients with a high volume of cancer or those with newly diagnosed metastatic disease. These findings suggest that triplet therapy could be a more effective treatment option for certain groups of patients, helping to improve outcomes and potentially changing the standard approach to managing mHSPC.

Evaluation of the Simple Summary

**Assessment Category**	**Score**	**Assessment Category**	**Score**	**Assessment Category**	**Score**
Comprehension Score	5	Gunning Fog Score	19.6	Words	125
Accuracy Score	1	SMOG Index	14.1	Complex words	30
Reading Age (years)	23.5	Coleman Liau Index	18.8	Percent of complex words	24.0
Flesch Kincaid Reading Ease	17.0	Automated Readability Index	18.7	Average words per sentence	25.0
Flesch Kincaid Grade Level	17.1	Sentences	5	Average syllables per word	1.9

Lay Summary (extended Prompt)

This study reviews the benefits of adding new androgen receptor pathway inhibitors (ARPI) to traditional treatments for metastatic hormone-sensitive prostate cancer (mHSPC). Previous treatments included docetaxel chemotherapy and androgen deprivation therapy (ADT), which improved survival. However, recent trials (PEACE-1 and ARASENS) show that combining ARPI with docetaxel and ADT (triplet therapy) can further improve overall survival, particularly for patients with high-volume cancer or newly diagnosed metastatic disease. The review analyzed five clinical trials involving 2,538 patients. Results show that triplet therapy significantly increased survival rates and reduced disease progression, especially in patients with high-volume or de novo metastatic cancer. These findings suggest that triplet therapy could become a new standard treatment for certain patients with mHSPC.

Evaluation of the Simple Summary

**Assessment Category**	**Score**	**Assessment Category**	**Score**	**Assessment Category**	**Score**
Comprehension Score	5	Gunning Fog Score	19.3	Words	121
Accuracy Score	0	SMOG Index	13.9	Complex words	35
Reading Age (years)	22.5	Coleman Liau Index	20.3	Percent of complex words	28.9
Flesch Kincaid Reading Ease	14.4	Automated Readability Index	17.5	Average words per sentence	20.2
Flesch Kincaid Grade Level	16.3	Sentences	6	Average syllables per word	2.0

Manuscript 15

Mason JB, Hatch L, Dall C, Kowalczyk KJ. Salvage Retzius-Sparing Radical Prostatectomy: A Review of Complications, Functional Outcomes, and Oncologic Outcomes. Curr Oncol. 2022 Dec 8;29(12):9733-9743. doi: 10.3390/curroncol29120764. PMID: 36547178; PMCID: PMC9777071.

Abstract

(Background) Radiation failure for localized prostate cancer is seen in 20-60% of patients who do not undergo extirpative surgery. Though potentially curative, salvage prostatectomy (SS) has not been frequently performed historically due to high rates of complications and postoperative incontinence. With the advent of robotic-assisted radical prostatectomy, these rates appear to be improved. Retzius-sparing approaches have additionally been shown to improve continence outcomes in the index setting, and may further improve continence outcomes in salvage cases while maintaining oncologic integrity. (Methods) We performed a literature review and qualitative analysis of published papers on salvage Retzius-sparing robotic-assisted radical prostatectomy (SRS). Three studies met criteria and were included in analysis. (Results) There were more patients with Gleason Grade Group 1 disease after initial treatment in the SRS group vs. SS (22% vs. 8%). Patients most frequently underwent external beam radiation therapy in both groups (52% vs. 49%). 30-day complication rates were 10% and 26% for SRS and SS, respectively. Continence outcomes were significantly improved in SRS with 59% of continence (based on study criteria) compared to 38% in SS. Time to continence was similarly improved for SRS. Positive surgical margins and biochemical recurrence were not significantly different between SRS and SS in any study. (Conclusions) SRS is a safe and feasible option for salvage treatment of localized prostate cancer and may improve postoperative continence outcomes. Positive surgical margin and biochemical recurrence rates are similar to those reported in SS.

Keywords: margins of excision; prostatic neoplasms; radiotherapy; salvage therapy; urinary incontinence.

Lay Summary (simple Prompt)

This study reviews the effectiveness and safety of salvage Retzius-sparing robotic-assisted radical prostatectomy (SRS) for patients whose prostate cancer has returned after initial radiation therapy. Traditional salvage surgery has often led to high rates of complications and incontinence. However, newer robotic techniques, especially those that avoid disturbing the Retzius space, seem to improve these outcomes. The review analyzed three studies and found that SRS had fewer complications and better urinary continence compared to traditional salvage surgery, while maintaining similar cancer control. The findings suggest that SRS is a viable option for treating recurrent localized prostate cancer, offering a balance between effectively managing the cancer and reducing post-surgical side effects like incontinence. This could lead to improved quality of life for patients undergoing salvage surgery.

Evaluation of the Simple Summary

**Assessment Category**	**Score**	**Assessment Category**	**Score**	**Assessment Category**	**Score**
Comprehension Score	5	Gunning Fog Score	20.4	Words	127
Accuracy Score	1	SMOG Index	14.9	Complex words	40
Reading Age (years)	23.5	Coleman Liau Index	19.9	Percent of complex words	31.5
Flesch Kincaid Reading Ease	16.2	Automated Readability Index	17.7	Average words per sentence	21.2
Flesch Kincaid Grade Level	16.3	Sentences	6	Average syllables per word	2.0

Lay Summary (extended Prompt)

This study reviews the use of salvage Retzius-sparing robotic-assisted radical prostatectomy (SRS) for treating prostate cancer that has returned after radiation therapy. Traditional salvage prostate surgery (SS) has been associated with high complication rates and urinary incontinence, but robotic techniques have improved these outcomes. The review compared SRS with standard SS and found that SRS resulted in fewer complications (10% vs. 26%) and better urinary control (59% vs. 38%). Both methods were equally effective in preventing cancer recurrence and achieving clear surgical margins. These findings suggest that SRS is a safer option that can improve patients' quality of life by reducing the risk of incontinence while still effectively treating recurrent prostate cancer.

Evaluation of the Simple Summary

**Assessment Category**	**Score**	**Assessment Category**	**Score**	**Assessment Category**	**Score**
Comprehension Score	5	Gunning Fog Score	16.3	Words	114
Accuracy Score	1	SMOG Index	11.8	Complex words	29
Reading Age (years)	19.5	Coleman Liau Index	17.4	Percent of complex words	25.4
Flesch Kincaid Reading Ease	32.2	Automated Readability Index	13.2	Average words per sentence	16.3
Flesch Kincaid Grade Level	12.8	Sentences	7	Average syllables per word	1.9

Manuscript 16

Hao Y, Zhang Q, Hang J, Xu L, Zhang S, Guo H. Development of a Prediction Model for Positive Surgical Margin in Robot-Assisted Laparoscopic Radical Prostatectomy. Curr Oncol. 2022 Dec 6;29(12):9560-9571. doi: 10.3390/curroncol29120751. PMID: 36547165; PMCID: PMC9776736.

Abstract

A positive surgical margin (PSM) is reported to have some connection to the occurrence of biochemical recurrence and tumor metastasis in prostate cancer after the operation. There are no clinically usable models and the study is to predict the probability of PSM after robot-assisted laparoscopic radical prostatectomy (RALP) based on preoperative examinations. It is a retrospective cohort from a single center. The Lasso method was applied for variable screening; logistic regression was employed to establish the final model; the strengthened bootstrap method was adopted for model internal verification; the nomogram and web calculator were used to visualize the model. All the statistical analyses were based on the R-4.1.2. The main outcome was a pathologically confirmed PSM. There were 151 PSMs in the 903 patients, for an overall positive rate of 151/903 = 16.7%; 0.727 was the adjusted C statistic, and the Brier value was 0.126. Hence, we have developed and validated a predictive model for PSM after RALP for prostate cancer that can be used in clinical practice. In the meantime, we observed that the International Society of Urological Pathology (ISUP) score, Prostate Imaging Reporting and Data System (PI-RADS) score, and Prostate-Specific Antigen (PSA) were the independent risk factors for PSM.

Keywords: positive surgical margin (PSM); prediction model; robot-assisted laparoscopic radical prostatectomy (RALP).

Lay Summary (simple Prompt)

This study aimed to develop a tool to predict the likelihood of a positive surgical margin (PSM) in patients undergoing robot-assisted laparoscopic radical prostatectomy (RALP) for prostate cancer. A PSM, where cancer cells are found at the edge of the removed tissue, can increase the risk of cancer recurrence and spread. By analyzing data from 903 patients, researchers identified key factors that increase the risk of PSM, such as the International Society of Urological Pathology (ISUP) score, Prostate Imaging Reporting and Data System (PI-RADS) score, and Prostate-Specific Antigen (PSA) levels. They used these factors to create a predictive model that can help doctors assess the risk of PSM before surgery. This model, which has been validated for clinical use, could help guide surgical decisions and improve patient outcomes by reducing the chances of cancer returning

Evaluation of the Simple Summary

**Assessment Category**	**Score**	**Assessment Category**	**Score**	**Assessment Category**	**Score**
Comprehension Score	5	Gunning Fog Score	17.1	Words	138
Accuracy Score	0	SMOG Index	13.9	Complex words	29
Reading Age (years)	21.5	Coleman Liau Index	14.8	Percent of complex words	21.0
Flesch Kincaid Reading Ease	31.1	Automated Readability Index	16.9	Average words per sentence	27.6
Flesch Kincaid Grade Level	15.8	Sentences	5	Average syllables per word	1.8

Lay Summary (extended Prompt)

This study aimed to develop a tool to predict the likelihood of a positive surgical margin (PSM) in patients undergoing robot-assisted laparoscopic radical prostatectomy (RALP) for prostate cancer. A PSM occurs when cancer cells are found at the edge of the removed tissue, which may increase the risk of cancer returning or spreading. The researchers analyzed data from 903 patients and found that 16.7% had a PSM. They identified key risk factors, including the ISUP score, PI-RADS score, and PSA levels. Using this information, they created and validated a prediction model that can help doctors estimate the probability of PSM after surgery. This model can guide treatment decisions and improve patient outcomes by identifying those at higher risk of PSM before surgery.

Evaluation of the Simple Summary

**Assessment Category**	**Score**	**Assessment Category**	**Score**	**Assessment Category**	**Score**
Comprehension Score	5	Gunning Fog Score	14.5	Words	125
Accuracy Score	0	SMOG Index	10.5	Complex words	23
Reading Age (years)	17.5	Coleman Liau Index	13.8	Percent of complex words	18.4
Flesch Kincaid Reading Ease	43.9	Automated Readability Index	11.2	Average words per sentence	17.9
Flesch Kincaid Grade Level	11.6	Sentences	7	Average syllables per word	1.7

Manuscript 17

Salciccia S, Frisenda M, Bevilacqua G, Viscuso P, Casale P, De Berardinis E, Di Pierro GB, Cattarino S, Giorgino G, Rosati D, Del Giudice F, Carbone A, Pastore A, Chung BI, Eisenberg ML, Autorino R, Crivellaro S, Forte F, Sciarra A, Mariotti G, Gentilucci A. Comparative Prospective and Longitudinal Analysis on the Platelet-to-Lymphocyte, Neutrophil-to-Lymphocyte, and Albumin-to-Globulin Ratio in Patients with Non-Metastatic and Metastatic Prostate Cancer. Curr Oncol. 2022 Dec 3;29(12):9474-9500. doi: 10.3390/curroncol29120745. PMID: 36547159; PMCID: PMC9777061.

Abstract

Purpose: To prospectively evaluate the albumin/globulin ratio (AGR), neutrophil/lymphocyte ratio (NLR), and platelet/lymphocyte ratio (PLR) diagnostic and prognostic predictive value in a stratified population of prostate cancer (PC) cases.

Methods: Population was divided based on the clinical and histologic diagnosis in: Group A: benign prostatic hyperplasia (BPH) cases (494 cases); Group B: all PC cases (525 cases); Group B1: clinically significant PC (426 cases); Group B2: non-metastatic PC (416 cases); Group B3: metastatic PC (109 cases). NLR, PLR, and AGR were obtained at the time of the diagnosis, and only in cases with PC considered for radical prostatectomy, determinations were also repeated 90 days after surgery. For each ratio, cut-off values were determined by receiver operating characteristics curve (ROC) analysis and fixed at 2.5, 120.0, and 1.4, respectively, for NLR, PLR, and AGR.

Results: Accuracy in predictive value for an initial diagnosis of clinically significant PC (csPC) was higher using PLR (0.718) when compared to NLR (0.220) and AGR (0.247), but, despite high sensitivity (0.849), very low specificity (0.256) was present. The risk of csPC significantly increased only according to PLR with an OR = 1.646. The percentage of cases with metastatic PC significantly increased according to high NLR and high PLR. Accuracy was 0.916 and 0.813, respectively, for NLR and PLR cut-off, with higher specificity than sensitivity. The risk of a metastatic disease increased 3.2 times for an NLR > 2.5 and 5.2 times for a PLR > 120 and at the multivariate analysis.

Conclusion: PLR and NLR have a significant predictive value towards the development of metastatic disease but not in relation to variations in aggressiveness or T staging inside

the non-metastatic PC. Our results suggest an unlikely introduction of these analyses into clinical practice in support of validated PC risk predictors.

Keywords: albumin-to-globulin ratio; metastatic; neutrophil-to-lymphocyte ratio; platelet-to-lymphocyte ratio; prostatic neoplasm; radical prostatectomy.

Lay Summary (simple Prompt)

This study examined whether certain blood test markers, like the ratios of neutrophils to lymphocytes (NLR), platelets to lymphocytes (PLR), and albumin to globulin (AGR), could help predict the diagnosis and progression of prostate cancer. Researchers looked at these markers in different groups, including patients with benign prostate conditions, non-metastatic prostate cancer, and metastatic prostate cancer. The findings showed that a higher PLR could indicate a greater risk of significant prostate cancer, while both high NLR and PLR were associated with a higher chance of the cancer spreading. However, these markers did not effectively predict the aggressiveness of non-metastatic cancer. The study suggests that while these blood tests may help identify patients at risk for metastatic prostate cancer, they are unlikely to replace current methods used to assess cancer risk and progression in clinical practice.

Evaluation of the Simple Summary

**Assessment Category**	**Score**	**Assessment Category**	**Score**	**Assessment Category**	**Score**
Comprehension Score	5	Gunning Fog Score	19.1	Words	137
Accuracy Score	0	SMOG Index	13.6	Complex words	28
Reading Age (years)	22.5	Coleman Liau Index	17.2	Percent of complex words	20.4
Flesch Kincaid Reading Ease	32.7	Automated Readability Index	18.6	Average words per sentence	27.4
Flesch Kincaid Grade Level	15.5	Sentences	5	Average syllables per word	1.7

Lay Summary (extended Prompt)

This study aimed to evaluate the usefulness of three blood markers—the albumin/globulin ratio (AGR), neutrophil/lymphocyte ratio (NLR), and platelet/lymphocyte ratio (PLR)—in predicting the diagnosis and progression of prostate cancer. Researchers analyzed data from patients with benign prostate conditions, non-metastatic prostate cancer, and metastatic prostate cancer. The study found that the PLR and NLR were effective in predicting the likelihood of prostate cancer spreading to other parts of the body (metastasis). Specifically, higher PLR and NLR were linked to a greater risk of metastatic prostate cancer. However, these markers did not help in predicting cancer aggressiveness or stage within non-metastatic cases. The study suggests that while these blood markers may have value in predicting metastasis, they are unlikely to replace current prostate cancer risk assessment tools in routine clinical practice.

Evaluation of the Simple Summary

**Assessment Category**	**Score**	**Assessment Category**	**Score**	**Assessment Category**	**Score**
Comprehension Score	5	Gunning Fog Score	18.5	Words	132
Accuracy Score	1	SMOG Index	13.3	Complex words	32
Reading Age (years)	21.5	Coleman Liau Index	18.4	Percent of complex words	24.2
Flesch Kincaid Reading Ease	25.6	Automated Readability Index	16.9	Average words per sentence	22.0
Flesch Kincaid Grade Level	15.2	Sentences	6	Average syllables per word	1.9

Manuscript 18

Oderda M, Calleris G, D'Agate D, Falcone M, Faletti R, Gatti M, Marra G, Marquis A, Gontero P. Intraoperative 3D-US-mpMRI Elastic Fusion Imaging-Guided Robotic Radical Prostatectomy: A Pilot Study. Curr Oncol. 2022 Dec 22;30(1):110-117. doi: 10.3390/curroncol30010009. PMID: 36661658; PMCID: PMC9858363.

Abstract

Introduction: When performing a nerve-sparing (NS) robotic radical prostatectomy (RARP), cancer location based on multiparametric MRI (mpMRI) is essential, as well as the location of positive biopsy cores outside mpMRI targets. The aim of this pilot study was to assess the feasibility of intraoperative 3D-TRUS-mpMRI elastic fusion imaging to guide RARP and to evaluate its impact on the surgical strategy. Methods: We prospectively enrolled 11 patients with organ-confined mpMRI-visible prostate cancer (PCa), histologically confirmed at transperineal fusion biopsy using Koelis Trinity. Before surgery, the 3D model of the prostate generated at biopsy was updated, showing both mpMRI lesions and positive biopsy cores, and was displayed on the Da Vinci robotic console using TilePro™ function. Results: Intraoperative 3D modeling was feasible in all patients (median of 6 min). The use of 3D models led to a major change in surgical strategy in six cases (54%), allowing bilateral instead of monolateral NS, or monolateral NS instead of non-NS, to be performed. At pathologic examination, no positive surgical margins (PSMs) were reported. Bilateral PCa presence was detected in one (9%), four (36%), and nine (81%) patients after mpMRI, biopsy, and RARP, respectively. Extracapsular extension was found in two patients (18%) even if it was not suspected at MRI. Conclusions: Intraoperative 3D-TRUS-mpMRI modeling with Koelis Trinity is feasible and reliable, helping the surgeon to maximize functional outcomes without increasing the risk of positive surgical margins. The location of positive biopsy cores must be registered in 3D models, given the rates of bilateral involvement not seen at mpMRI.

Keywords: elastic fusion; intraoperative; robotic radical prostatectomy; 3D; ultrasound

Lay Summary (simple Prompt)

This pilot study explored the use of a new imaging technique combining 3D ultrasound with MRI to guide robotic prostate surgery for cancer. The goal was to see if this approach could help surgeons better identify cancer locations during surgery, allowing for more precise removal while sparing healthy nerves. Eleven patients with prostate cancer confined to the organ participated in the study. The imaging technique was successful in all cases and led to changes in surgical plans for over half of the patients, allowing for more nerve-sparing procedures. No patients had positive surgical margins, meaning all cancerous tissue was effectively removed. This approach could help improve outcomes by preserving more normal tissue and function without increasing the risk of leaving cancer behind, making it a promising tool for prostate cancer surgery.

Evaluation of the Lay Summary

**Assessment Category**	**Score**	**Assessment Category**	**Score**	**Assessment Category**	**Score**
Comprehension Score	5	Gunning Fog Score	17.0	Words	132
Accuracy Score	0	SMOG Index	12.3	Complex words	27
Reading Age (years)	20.5	Coleman Liau Index	15.9	Percent of complex words	20.5
Flesch Kincaid Reading Ease	37.1	Automated Readability Index	14.9	Average words per sentence	22.0
Flesch Kincaid Grade Level	13.6	Sentences	6	Average syllables per word	1.7

Lay Summary (extended Prompt)

This pilot study explored a new method to improve prostate cancer surgery by using 3D ultrasound and MRI fusion imaging to guide robotic prostate removal. The goal was to see if this imaging technology could help surgeons plan better and spare important nerves, which can improve recovery after surgery. Eleven patients with prostate cancer were included. A 3D model of the prostate, showing both MRI-identified cancer areas and biopsy results, was created and displayed during surgery. This approach changed the surgical plan in 54% of cases, allowing for more precise nerve-sparing techniques. No patients had cancer left at the surgical edges, reducing the chance of cancer returning. The study concluded that using 3D imaging during surgery is feasible and can improve outcomes without increasing risks.

Evaluation of the Lay Summary

**Assessment Category**	**Score**	**Assessment Category**	**Score**	**Assessment Category**	**Score**
Comprehension Score	5	Gunning Fog Score	15.4	Words	127
Accuracy Score	0	SMOG Index	11.2	Complex words	26
Reading Age (years)	18.5	Coleman Liau Index	14.9	Percent of complex words	20.5
Flesch Kincaid Reading Ease	41.2	Automated Readability Index	12.7	Average words per sentence	18.1
Flesch Kincaid Grade Level	12.0	Sentences	7	Average syllables per word	1.7

Manuscript 19

Wardale L, Cardenas R, Gnanapragasam VJ, Cooper CS, Clark J, Brewer DS. Combining Molecular Subtypes with Multivariable Clinical Models Has the Potential to Improve Prediction of Treatment Outcomes in Prostate Cancer at Diagnosis. Curr Oncol. 2022 Dec 22;30(1):157-170. doi: 10.3390/curroncol30010013. PMID: 36661662; PMCID: PMC9857957.

Abstract

Clinical management of prostate cancer is challenging because of its highly variable natural history and so there is a need for improved predictors of outcome in non-metastatic men at the time of diagnosis. In this study we calculated the model score from the leading clinical multivariable model, PREDICT prostate, and the poor prognosis DESNT molecular subtype, in a combined expression and clinical dataset that were taken from malignant tissue at prostatectomy (n = 359). Both PREDICT score ($p < 0.0001$, IQR HR = 1.59) and DESNT score ($p < 0.0001$, IQR HR = 2.08) were significant predictors for time to biochemical recurrence. A joint model combining the continuous PREDICT and DESNT score ($p < 0.0001$, IQR HR = 1.53 and 1.79, respectively) produced a significantly improved predictor than either model alone ($p < 0.001$). An increased probability of mortality after diagnosis, as estimated by PREDICT, was characterised by upregulation of cell-cycle related pathways and the downregulation of metabolism and cholesterol biosynthesis. The DESNT molecular subtype has distinct biological characteristics to those associated with the PREDICT model. We conclude that the inclusion of biological information alongside current clinical prognostic tools has the potential to improve the ability to choose the optimal treatment pathway for a patient.

Keywords:

prostate cancer; clinical models; predictive models; molecular subtypes; transcriptome; expression; statistical model

Lay Summary (simple Prompt)

This study aimed to improve predictions of prostate cancer outcomes by combining a clinical tool with new molecular information. Prostate cancer can behave very differently from patient to patient, making it hard to predict how it will progress. Currently, doctors use clinical models, like PREDICT, to estimate a patient's risk, but these models can be limited. This study looked at adding a molecular subtype score, called DESNT, to the PREDICT model using data from 359 prostate cancer patients. Both the PREDICT and DESNT scores were effective on their own in predicting cancer recurrence, but the combination of the two provided even better predictions. By integrating this molecular information with clinical tools, the research suggests it may be possible to more accurately predict patient outcomes and tailor treatment plans, potentially leading to better management of prostate cancer.

Evaluation of the Lay Summary

**Assessment Category**	**Score**	**Assessment Category**	**Score**	**Assessment Category**	**Score**
Comprehension Score	5	Gunning Fog Score	16.4	Words	136
Accuracy Score	0	SMOG Index	11.8	Complex words	25
Reading Age (years)	20.5	Coleman Liau Index	16.2	Percent of complex words	18.4
Flesch Kincaid Reading Ease	43.5	Automated Readability Index	15.5	Average words per sentence	22.7
Flesch Kincaid Grade Level	14.1	Sentences	6	Average syllables per word	1.8

Lay Summary (extended Prompt)

This study explores how combining clinical models with molecular subtypes can improve predictions for prostate cancer outcomes at diagnosis. Prostate cancer varies greatly in its progression, making it hard to predict which treatment will work best. Researchers combined two tools: the PREDICT clinical model, which uses patient data to estimate the risk of recurrence, and the DESNT molecular subtype, which identifies cancer types based on their biology. Both models on their own were strong predictors of recurrence, but when combined, they provided an even more accurate prediction. This approach, which integrates biological information with clinical data, has the potential to help doctors make better decisions about the most effective treatment for each patient.

Evaluation of the Lay Summary

Assessment Category	Score	Assessment Category	Score	Assessment Category	Score
Comprehension Score	5	Gunning Fog Score	17.9	Words	113
Accuracy Score	1	SMOG Index	12.9	Complex words	25
Reading Age (years)	21.5	Coleman Liau Index	17.8	Percent of complex words	22.1
Flesch Kincaid Reading Ease	31.9	Automated Readability Index	16.7	Average words per sentence	22.6
Flesch Kincaid Grade Level	14.4	Sentences	5	Average syllables per word	1.8

Manuscript 20

Ahmad Khalil D, Wulff J, Jazmati D, Geismar D, Bäumer C, Kramer PH, Steinmeier T, Schleithoff SS, Tschirdewahn S, Hadaschik B, Timmermann B. Is an Endorectal Balloon Beneficial for Rectal Sparing after Spacer Implantation in Prostate Cancer Patients Treated with Hypofractionated Intensity-Modulated Proton Beam Therapy? A Dosimetric and Radiobiological Comparison Study. Curr Oncol. 2023 Jan 6;30(1):758-768. doi: 10.3390/curroncol30010058. PMID: 36661707; PMCID: PMC9857887.

Abstract

Background: The aim of this study is to examine the dosimetric influence of endorectal balloons (ERB) on rectal sparing in prostate cancer patients with implanted hydrogel rectum spacers treated with dose-escalated or hypofractionated intensity-modulated proton beam therapy (IMPT). Methods: Ten patients with localized prostate cancer included in the ProRegPros study and treated at our center were investigated. All patients underwent placement of hydrogel rectum spacers before planning. Two planning CTs (with and without 120 cm3 fluid-filled ERB) were applied for each patient. Dose prescription was set according to the h strategy, with 72 Gray (Gy)/2.4 Gy/5× weekly to prostate + 1 cm of the seminal vesicle, and 60 Gy/2 Gy/5× weekly to prostate + 2 cm of the seminal vesicle. Planning with two laterally opposed IMPT beams was performed in both CTs. Rectal dosimetry values including dose-volume statistics and normal tissue complication probability (NTCP) were compared for both plans (non-ERB plans vs. ERB plans). Results: For ERB plans compared with non-ERB, the reductions were 8.51 ± 5.25 Gy (RBE) ($p = 0.000$) and 15.76 ± 11.11 Gy ($p = 0.001$) for the mean and the median rectal doses, respectively. No significant reductions in rectal volumes were found after high dose levels. The use of ERB resulted in significant reduction in rectal volume after receiving 50 Gy (RBE), 40 Gy (RBE), 30 Gy (RBE), 20 Gy (RBE), and 10 Gy (RBE) with p values of 0.034, 0.008, 0.003, 0.001, and 0.001, respectively. No differences between ERB and non-ERB plans for the anterior rectum were observed. ERB reduced posterior rectal volumes in patients who received 30 Gy (RBE), 20 Gy (RBE), or 10 Gy (RBE), with p values of 0.019, 0.003, and 0.001, respectively. According to the NTCP models, no significant reductions were observed in mean or median rectal toxicity (late rectal bleeding ≥ 2, necrosis or stenosis, and late rectal toxicity ≥ 3) when using the ERB. Conclusion: ERB

reduced rectal volumes exposed to intermediate or low dose levels. However, no significant reduction in rectal volume was observed in patients receiving high or intermediate doses. There was no benefit and also no disadvantage associated with the use of ERB for late rectal toxicity, according to available NTCP models.

Keywords:

endorectal balloon; proton therapy; intensity-modulated therapy; prostate cancer; dose-escalated radiation therapy; hypofractionated radiation therapy

Lay Summary (simple Prompt)

This study explored whether using an endorectal balloon (ERB) during proton beam therapy for prostate cancer could better protect the rectum from radiation damage. The research involved ten prostate cancer patients who had hydrogel spacers placed between their prostate and rectum to minimize radiation exposure to the rectum. The results showed that the ERB significantly reduced the amount of rectal tissue exposed to lower and moderate radiation doses, but it did not significantly reduce exposure at higher doses. Additionally, the use of ERB did not show a significant benefit in reducing serious long-term rectal side effects. Therefore, while the ERB helped reduce radiation exposure to the rectum at lower doses, it did not provide additional advantages for preventing severe rectal damage. These findings are important for guiding future decisions on the use of ERBs in prostate cancer radiation therapy.

Evaluation of the Lay Summary

**Assessment Category**	**Score**	**Assessment Category**	**Score**	**Assessment Category**	**Score**
Comprehension Score	5	Gunning Fog Score	16.8	Words	140
Accuracy Score	0	SMOG Index	12.0	Complex words	26
Reading Age (years)	20.5	Coleman Liau Index	16.2	Percent of complex words	18.6
Flesch Kincaid Reading Ease	33.3	Automated Readability Index	15.9	Average words per sentence	23.3
Flesch Kincaid Grade Level	14.4	Sentences	6	Average syllables per word	1.8

Lay Summary (extended Prompt)

This study looked at whether using an endorectal balloon (ERB) can help protect the rectum from radiation during proton beam therapy for prostate cancer patients who have had a hydrogel spacer implanted. Proton therapy targets cancer cells with high precision, but it can still affect nearby tissues, like the rectum. Ten patients with localized prostate cancer were studied, and treatment plans were compared with and without the ERB. The results showed that using the ERB reduced the amount of radiation exposure to the rectum at lower dose levels but didn't make a significant difference at higher doses. Additionally, no improvements were seen in terms of reducing long-term rectal side effects. Overall, the ERB helped reduce radiation to the rectum at lower levels but did not affect more serious rectal damage risks.

Evaluation of the Lay Summary

**Assessment Category**	**Score**	**Assessment Category**	**Score**	**Assessment Category**	**Score**
Comprehension Score	5	Gunning Fog Score	14.3	Words	132
Accuracy Score	0	SMOG Index	10.1	Complex words	18
Reading Age (years)	18.5	Coleman Liau Index	14.4	Percent of complex words	13.6
Flesch Kincaid Reading Ease	45.4	Automated Readability Index	13.8	Average words per sentence	22.0
Flesch Kincaid Grade Level	12.4	Sentences	6	Average syllables per word	1.6

Manuscript 21

Cincidda C, Pizzoli SFM, Ongaro G, Oliveri S, Pravettoni G. Caregiving and Shared Decision Making in Breast and Prostate Cancer Patients: A Systematic Review. Curr Oncol. 2023 Jan 6;30(1):803-823. doi: 10.3390/curroncol30010061. PMID: 36661710; PMCID: PMC9857468.

Abstract

Background: A cancer diagnosis can impact patients' and caregivers' lives, posing different challenging situations. In particular, breast cancer and prostate cancer are two types of cancer involving families and especially spouses in challenges linked with the diagnosis and treatment process. Caregivers are usually involved in the treatment decision-making (TDM) process concerning patients' clinical pathway, cancer treatment, and ongoing therapies. To date, no contributions provide an exhaustive overview of the role of caregivers in cancer care and their involvement in the TDM process related to the therapies. Methods: We performed a systematic review of caregiver and patients experiences and perceptions of caregiver involvement in cancer TDM. Articles were searched on Public/Publisher MEDLINE (PubMed), Excerpta Medica Database (Embase), Medical Literature Analysis and Retrieval System Online (Medline), and American Psychological Association APA PsycINFO. Results: 17 studies were included, 10 on prostate cancer and 7 on breast cancer. According to the reviewed studies, patients and caregivers experienced the cancer diagnosis with a sense of unity. Most patients preferred to have an active or collaborative role with caregivers in TDM, feeling it was important to consult or share the decision made with their caregivers. Caregivers preferred to collaborate with patients or let patients decide by themselves after considering their opinions. Caregiver involvement could have a positive influence on the patient's medical decisions, even if cancer diagnosis and treatments overwhelmed patients and caregivers. Conclusions: These findings highlight the importance of using a perspective that focuses on the relationship between a patient and caregivers when they receive a cancer diagnosis and have to make a treatment decision. Targeting caregiver–patient dyads, rather than individuals, is important since a supported relationship could have a protective effect on psychological distress, quality of life (QOL), and relationship

satisfaction. Moreover, dyads may benefit from interventions that focus on the needs of both the patient and caregiver.

Keywords: caregivers; prostate cancer; breast cancer; decision-making

Lay Summary (simple Prompt)

This study examines the important role that caregivers, especially spouses, play in supporting breast and prostate cancer patients during their diagnosis and treatment. It reviews existing research to understand how caregivers are involved in treatment decision-making (TDM). The findings suggest that most patients and caregivers prefer to work together when making treatment decisions, feeling that collaboration improves decision quality and strengthens their relationship. However, the process can be overwhelming for both parties. The study highlights the importance of focusing on the patient-caregiver relationship, rather than just the patient, to improve emotional well-being, quality of life, and satisfaction with the treatment process. It also suggests that targeted interventions addressing the needs of both patients and caregivers could be beneficial.

Evaluation of the Lay Summary

**Assessment Category**	**Score**	**Assessment Category**	**Score**	**Assessment Category**	**Score**
Comprehension Score	5	Gunning Fog Score	18.3	Words	121
Accuracy Score	1	SMOG Index	13.1	Complex words	31
Reading Age (years)	22.5	Coleman Liau Index	19.7	Percent of complex words	25.6
Flesch Kincaid Reading Ease	23.5	Automated Readability Index	17.1	Average words per sentence	20.2
Flesch Kincaid Grade Level	15.0	Sentences	6	Average syllables per word	1.9

Lay Summary (extended Prompt)

This study reviews the role of caregivers in decision-making for patients with breast and prostate cancer. Both types of cancer often involve not just the patient but also their family, especially spouses, in making important decisions about treatment. The review looked at 17 studies to understand how patients and caregivers experience this process. It found that many patients prefer to involve their caregivers in decisions, seeing it as important to share or consult with them. Caregivers often want to help patients make decisions, or allow patients to decide after discussing options together. Involving caregivers can have a positive effect on the patient's treatment choices and emotional well-being. The study highlights the importance of supporting both the patient and caregiver during this challenging time, as their relationship can help reduce stress and improve their quality of life.

Evaluation of the Lay Summary

Assessment Category	Score	Assessment Category	Score	Assessment Category	Score
Comprehension Score	5	Gunning Fog Score	16.0	Words	138
Accuracy Score	0	SMOG Index	11.6	Complex words	28
Reading Age (years)	19.5	Coleman Liau Index	16.1	Percent of complex words	20.3
Flesch Kincaid Reading Ease	40.3	Automated Readability Index	14.0	Average words per sentence	19.7
Flesch Kincaid Grade Level	12.5	Sentences	7	Average syllables per word	1.7

Manuscript 22

Grubmüller B, Huebner NA, Rasul S, Clauser P, Pötsch N, Grubmüller KH, Hacker M, Hartenbach S, Shariat SF, Hartenbach M, Baltzer P. Dual-Tracer PET-MRI-Derived Imaging Biomarkers for Prediction of Clinically Significant Prostate Cancer. Curr Oncol. 2023 Jan 30;30(2):1683-1691. doi: 10.3390/curroncol30020129. PMID: 36826090; PMCID: PMC9954891.

Abstract

Purpose: To investigate if imaging biomarkers derived from 3-Tesla dual-tracer [(18)F]fluoromethylcholine (FMC) and [68Ga]Ga-PSMAHBED-CC conjugate 11 (PSMA)-positron emission tomography can adequately predict clinically significant prostate cancer (csPC). Methods: We assessed 77 biopsy-proven PC patients who underwent 3T dual-tracer PET/mpMRI followed by radical prostatectomy (RP) between 2014 and 2017. We performed a retrospective lesion-based analysis of all cancer foci and compared it to whole-mount histopathology of the RP specimen. The primary aim was to investigate the pretherapeutic role of the imaging biomarkers FMC- and PSMA-maximum standardized uptake values (SUVmax) for the prediction of csPC and to compare it to the mpMRI-methods and PI-RADS score. Results: Overall, we identified 104 cancer foci, 69 were clinically significant (66.3%) and 35 were clinically insignificant (33.7%). We found that the combined FMC+PSMA SUVmax were the only significant parameters ($p < 0.001$ and $p = 0.049$) for the prediction of csPC. ROC analysis showed an AUC for the prediction of csPC of 0.695 for PI-RADS scoring (95% CI 0.591 to 0.786), 0.792 for FMC SUVmax (95% CI 0.696 to 0.869), 0.852 for FMC+PSMA SUVmax (95% CI 0.764 to 0.917), and 0.852 for the multivariable CHAID model (95% CI 0.763 to 0.916). Comparing the AUCs, we found that FMC+PSMA SUVmax and the multivariable model were significantly more accurate for the prediction of csPC compared to PI-RADS scoring ($p = 0.0123$, $p = 0.0253$, respectively). Conclusions: Combined FMC+PSMA SUVmax seems to be a reliable parameter for the prediction of csPC and might overcome the limitations of PI-RADS scoring. Further prospective studies are necessary to confirm these promising preliminary results.

Keywords: prostate cancer; PET/MRI; imaging biomarkers; dual tracer

Lay Summary (simple Prompt)

This study explores the use of advanced imaging techniques to better predict aggressive forms of prostate cancer. Researchers used two different tracers, FMC and PSMA, in a PET/MRI scan to identify prostate cancer in patients before they underwent surgery. They found that combining data from both tracers was more effective at predicting significant prostate cancer than the current standard method, called PI-RADS scoring. The combined imaging approach showed higher accuracy in detecting aggressive cancer types, which could help doctors make better treatment decisions. This research suggests that using dual-tracer PET/MRI could improve the way prostate cancer is diagnosed and managed, potentially leading to more personalized and effective treatment plans for patients. Further studies are needed to confirm these initial findings.

Evaluation of the Lay Summary

**Assessment Category**	**Score**	**Assessment Category**	**Score**	**Assessment Category**	**Score**
Comprehension Score	4	Gunning Fog Score	15.3	Words	122
Accuracy Score	0	SMOG Index	11.1	Complex words	22
Reading Age (years)	20.5	Coleman Liau Index	18.2	Percent of complex words	18.0
Flesch Kincaid Reading Ease	37.8	Automated Readability Index	15.9	Average words per sentence	20.3
Flesch Kincaid Grade Level	13.0	Sentences	6	Average syllables per word	1.8

Lay Summary (extended Prompt)

This study aimed to find better ways to predict clinically significant prostate cancer using advanced imaging techniques. Researchers used two imaging tracers, FMC and PSMA, along with PET/MRI scans to evaluate 77 prostate cancer patients who later underwent surgery. By analyzing 104 cancer areas, they discovered that combining the results of FMC and PSMA imaging was more accurate at predicting significant cancer compared to the commonly used PI-RADS scoring system. The combined imaging method had a high accuracy rate and outperformed traditional methods in identifying the more dangerous forms of prostate cancer. These findings suggest that dual-tracer PET/MRI could help doctors better detect and treat aggressive prostate cancer, but more research is needed to confirm these results.

Evaluation of the Lay Summary

**Assessment Category**	**Score**	**Assessment Category**	**Score**	**Assessment Category**	**Score**
Comprehension Score	4	Gunning Fog Score	17.6	Words	119
Accuracy Score	1	SMOG Index	12.7	Complex words	24
Reading Age (years)	21.5	Coleman Liau Index	16.8	Percent of complex words	20.2
Flesch Kincaid Reading Ease	30.5	Automated Readability Index	16.6	Average words per sentence	23.8
Flesch Kincaid Grade Level	14.9	Sentences	5	Average syllables per word	1.8

Manuscript 23

Liu W, Yu M, Cheng S, Zhou X, Li J, Lu Y, Liu P, Ding S. tRNA-Derived RNA Fragments Are Novel Biomarkers for Diagnosis, Prognosis, and Tumor Subtypes in Prostate Cancer. Curr Oncol. 2023 Jan 10;30(1):981-999. doi: 10.3390/curroncol30010075. PMID: 36661724; PMCID: PMC9857875.

Abstract:

Background: tRNA-derived RNA fragments (tRFs) are a novel class of small ncRNA that are derived from precursor or mature tRNAs. Recently, the general relevance of their roles and clinical values in tumorigenesis, metastasis, and recurrence have been increasingly highlighted. However, there has been no specific systematic study to elucidate any potential clinical significance for these tRFs in prostate adenocarcinoma (PRAD), one of the most common and malignant cancers that threatens male health worldwide. Here, we investigate the clinical value of 5'-tRFs in PRAD. Methods: Small RNA sequencing data were analyzed to discover new 5'-tRFs biomarkers for PRAD. Machine learning algorithms were used to identify 5'-tRF classifiers to distinguish PRAD tumors from normal tissues. LASSO and Cox regression analyses were used to construct 5'-tRF prognostic predictive models. NMF and consensus clustering analyses were performed on 5'-tRF profiles to identify molecular subtypes of PRAD. Results: The overall levels of 5'-tRFs were significantly upregulated in the PRAD tumor samples compared to their adjacent normal samples. tRF classifiers composed of 13 5'-tRFs achieved AUC values as high as 0.963, showing high sensitivity and specificity in distinguishing PRAD tumors from normal samples. Multiple 5'-tRFs were identified as being associated with the PRAD prognosis. The tRF score, defined by a set of eight 5'-tRFs, was highly predictive of survival in PRAD patients. The combination of tRF and Gleason scores showed a significantly better performance than the Gleason score alone, suggesting that 5'-tRFs can offer PRAD patients additional and improved prognostic information. Four molecular subtypes of the PRAD tumor were identified based on their 5'-tRF expression profiles. Genetically, these 5'-tRFs PRAD tumor subtypes exhibited distinct genomic landscapes in tumor cells. Clinically, they showed marked differences in survival and clinicopathological features. Conclusions: 5'-tRFs are potential clinical biomarkers for the diagnosis, prognosis, and classification of tumor subtypes on a molecular level. These can help clinicians

formulate personalized treatment plans for PRAD patients and may have similar potential applications for other disease types.

Keywords:
prostate adenocarcinoma; tRNA-derived RNA fragments; tumor subtypes; biomarker; diagnosis; prognosis

Lay Summary (simple Prompt)

This study explores the potential of a new type of molecule, called tRNA-derived RNA fragments (tRFs), as biomarkers for prostate cancer, one of the most common cancers in men. Researchers analyzed small RNA sequencing data to identify specific tRFs that could help distinguish prostate cancer tissues from normal tissues and predict patient outcomes. The findings showed that certain tRFs are significantly more present in prostate cancer tissues, and these can be used to classify tumors into subtypes with different genetic features and survival rates. By using these tRFs alongside traditional diagnostic tools, like the Gleason score, doctors can improve the accuracy of prostate cancer diagnosis, prognosis, and treatment planning. This research suggests that tRFs could be valuable for personalizing cancer treatment and might be applicable to other diseases as well.

Evaluation of the Lay Summary

**Assessment Category**	**Score**	**Assessment Category**	**Score**	**Assessment Category**	**Score**
Comprehension Score	5	Gunning Fog Score	17.5	Words	131
Accuracy Score	0	SMOG Index	12.4	Complex words	23
Reading Age (years)	21.5	Coleman Liau Index	17.0	Percent of complex words	17.6
Flesch Kincaid Reading Ease	31.7	Automated Readability Index	17.9	Average words per sentence	26.2
Flesch Kincaid Grade Level	15.3	Sentences	5	Average syllables per word	1.8

Lay Summary (extended Prompt)

This study explores a new class of small RNA molecules, called tRNA-derived RNA fragments (tRFs), as potential biomarkers for prostate cancer (PRAD). Biomarkers help doctors detect and predict the course of a disease. The researchers analyzed small RNA sequencing data to identify specific tRFs that can distinguish prostate cancer tissues from normal ones. They discovered that certain tRFs are highly elevated in prostate cancer and developed a model that can predict cancer with high accuracy. Additionally, they identified several tRFs linked to patient survival and created a tRF-based score that improves prognosis prediction when combined with the Gleason score (a common tool for assessing prostate cancer severity). The study also uncovered four distinct molecular subtypes of prostate cancer based on tRFs, each with unique genetic characteristics and survival outcomes. These findings suggest that tRFs could improve prostate cancer diagnosis, prognosis, and treatment personalization.

Evaluation of the Lay Summary

**Assessment Category**	**Score**	**Assessment Category**	**Score**	**Assessment Category**	**Score**
Comprehension Score	5	Gunning Fog Score	16.8	Words	145
Accuracy Score	0	SMOG Index	12.2	Complex words	31
Reading Age (years)	20.5	Coleman Liau Index	18.0	Percent of complex words	21.4
Flesch Kincaid Reading Ease	30.6	Automated Readability Index	16.0	Average words per sentence	20.7
Flesch Kincaid Grade Level	14.1	Sentences	7	Average syllables per word	1.8

Manuscript 24

Asimakopoulos AD, Annino F, Colalillo G, Gaston R, Piechaud T, Mauriello A, Anceschi U, Borri F. "Urethral-Sparing" Robotic Radical Prostatectomy: Critical Appraisal of the Safety of the Technique Based on the Histologic Characteristics of the Prostatic Urethra. Curr Oncol. 2023 Jan 12;30(1):1065-1076. doi: 10.3390/curroncol30010082. PMID: 36661731; PMCID: PMC9857678.

Abstract

Background: The prostatic urethra (PU) is conventionally resected during robot-assisted radical prostatectomy (RALP). Recent studies demonstrated the feasibility of the extended PU preservation (EPUP). Aims: To describe the histologic features of the PU. Methods: The PU was evaluated using cystoprostatectomy and RALP specimens. Cases of PU infiltration by prostate cancer or distortion by benign hyperplastic nodules were excluded. The thickness of the chorion and distance between the urothelium and prostate glands were measured. Prostate-specific antigen expression in the PU epithelium was evaluated with immunohistochemistry. Descriptive statistics were used. Results: Six specimens of PU were examined. Histologically, the following layers of the PU were observed: (1) urothelium with basal membrane, (2) chorion, and (3) prostatic peri-urethral fibromuscular tissue. The chorion measures between 0.2 and 0.4 mm. There is not a distinct urethral muscle layer, but rather muscular fibers that originate near the prostatic stroma and are distributed around the PU. This muscular tissue appears to be mainly represented in the basal and apical urethra, but not in the middle urethra. The mean distance between the chorion and prostatic glands is 1.74 mm, with significant differences between base of the prostate, middle urethral portion, and apex (2.5 vs. 1.49 vs. 1.23 mm, respectively). PSA-expressing cells are abundant in the PU epithelium, coexisting with urothelial cells. Conclusions: The exiguity of thickness of the PU chorion, short distance from glandular tissue, and coexistence of PSA-expressing cells in the epithelium raise important concerns about the oncologic safety of EPUP.

Keywords:

prostate cancer; incontinence; prostatectomy; urethra; urothelium

Lay Summary (simple Prompt)

This study investigates the safety of preserving the prostatic urethra (PU) during robotic prostate surgery, a procedure typically done to treat prostate cancer. Traditionally, the PU is removed, but recent techniques suggest it can be preserved to help improve outcomes like urinary control. The study examined the tissue structure of the PU to understand the potential risks of leaving it intact. Findings showed that the PU is very close to prostate tissue that can contain cancer cells, and the PU itself has cells that express prostate-specific antigen (PSA), which is linked to prostate cancer. These results raise concerns about the risk of cancer recurrence if the PU is not removed. The study suggests that more caution is needed when considering this preservation technique during prostate surgery to ensure patient safety.

Evaluation of the Lay Summary

Assessment Category	Score	Assessment Category	Score	Assessment Category	Score
Comprehension Score	5	Gunning Fog Score	15.8	Words	131
Accuracy Score	0	SMOG Index	11.3	Complex words	23
Reading Age (years)	19.5	Coleman Liau Index	15.1	Percent of complex words	17.6
Flesch Kincaid Reading Ease	41.3	Automated Readability Index	14.2	Average words per sentence	21.8
Flesch Kincaid Grade Level	12.9	Sentences	6	Average syllables per word	1.7

Lay Summary (extended Prompt)

This study explores the safety of preserving more of the urethra during robotic prostate removal surgery (robot-assisted radical prostatectomy), a technique known as "urethral-sparing." Typically, the urethra is removed along with the prostate during surgery, but some recent studies have shown that keeping more of it may be possible. The researchers examined six prostate specimens to understand the structure of the urethra and how close it is to cancerous tissue. They found that the urethra’s tissue is very thin, and in many cases, cancer-related cells are close to the urethra. The study raises concerns that preserving more of the urethra could increase the risk of leaving behind cancerous tissue. These findings suggest that while urethral-sparing techniques might help with urinary function after surgery, there could be risks in terms of fully removing the cancer.

Evaluation of the Lay Summary

**Assessment Category**	**Score**	**Assessment Category**	**Score**	**Assessment Category**	**Score**
Comprehension Score	3	Gunning Fog Score	17.9	Words	139
Accuracy Score	0	SMOG Index	13.3	Complex words	32
Reading Age (years)	20.5	Coleman Liau Index	14.8	Percent of complex words	23.0
Flesch Kincaid Reading Ease	37.9	Automated Readability Index	14.6	Average words per sentence	23.2
Flesch Kincaid Grade Level	13.7	Sentences	6	Average syllables per word	1.7

Manuscript 25

Yang CH, Chen LH, Lin YS, Hsu CY, Tung MC, Huang SW, Wu CH, Ou YC. Incorporating VR-RENDER Fusion Software in Robot-Assisted Partial Prostatectomy: The First Case Report. Curr Oncol. 2023 Jan 31;30(2):1699-1707. doi: 10.3390/curroncol30020131. PMID: 36826092; PMCID: PMC9955092.

Abstract

Currently, the active surveillance of men with favorable intermediate-risk localized prostate cancer (PCa) is a longstanding controversy, in terms of their oncological outcomes, and radical prostatectomy would constitute a similar concern of overtreatment, regarding its functional outcomes. Thus, focal therapy could be considered in men belonging to favorable intermediate-risk group. Among all focal therapies, high-intensity focused ultrasound (HIFU) was the most studied methodology in clinical trials. Although HIFU provided better functional outcomes than radical prostatecomy, the oncological outcomes were inferior in men with intermediate-risk localized PCa. Two articles have been published discussing the feasibility and clinical outcomes of robot-assisted partial prostatectomy (RAPP), and both the functional and oncological outcomes were superior than those with HIFU. However, the rate of positive surgical margins (PSMs) was reported as high in the literature. Here, we present a case of favorable intermediate-risk localized PCa with an isolated tumor at the anterior apex. After reconstructing a personal three-dimensional (3D) image, we utilized it in a 3D image-guided precise excise, followed by intraoperative frozen specimen review. We found that this method may present a resolution to the high PSM rate documented in the current literature regarding RAPP. This method merits further study with a well-designed prospective study.

Keywords:

robotic surgical procedures/adverse effects; prostatic neoplasms/pathology; prostatic neoplasms/diagnostic imaging; treatment outcome; feasibility studies; case reports

Lay Summary (simple Prompt)

This study explores the use of a new technology, VR-RENDER Fusion software, in performing a robot-assisted partial prostatectomy for a patient with localized prostate cancer at intermediate risk. Traditional treatment methods, like radical prostatectomy, can lead to significant side effects, while newer methods like high-intensity focused ultrasound (HIFU) have shown better functional outcomes but less effective cancer control. Robot-assisted partial prostatectomy (RAPP) has been shown to provide better outcomes than HIFU, but with a higher risk of incomplete cancer removal. In this case, a 3D model of the patient's prostate was created using VR-RENDER Fusion software to guide a precise surgical removal of the tumor. This approach helped achieve better surgical accuracy and may reduce the risk of leaving cancerous tissue behind. The study suggests that this innovative technique could improve outcomes for patients and should be further investigated in larger studies.

Evaluation of the Lay Summary

Assessment Category	Score	Assessment Category	Score	Assessment Category	Score
Comprehension Score	5	Gunning Fog Score	16.9	Words	147
Accuracy Score	1	SMOG Index	12.0	Complex words	26
Reading Age (years)	21.5	Coleman Liau Index	16.8	Percent of complex words	17.7
Flesch Kincaid Reading Ease	27.7	Automated Readability Index	16.9	Average words per sentence	24.5
Flesch Kincaid Grade Level	15.5	Sentences	6	Average syllables per word	1.8

Lay Summary (extended Prompt)

This study presents the first case of using VR-RENDER Fusion software during robot-assisted partial prostatectomy (RAPP) to improve surgical precision. Men with intermediate-risk localized prostate cancer often face a dilemma between active surveillance and radical prostatectomy, which may cause unnecessary side effects. Focal therapies, like high-intensity focused ultrasound (HIFU), have been explored but offer less favorable cancer control. RAPP has shown better cancer and functional outcomes but has a high rate of positive surgical margins (PSMs), meaning cancerous tissue may be left behind. In this case, 3D imaging was used to guide the surgery, helping to remove the tumor more precisely and potentially lowering the PSM rate. This approach could improve outcomes for men with intermediate-risk prostate cancer, and further studies are needed to confirm its benefits.

Evaluation of the Lay Summary

**Assessment Category**	**Score**	**Assessment Category**	**Score**	**Assessment Category**	**Score**
Comprehension Score	5	Gunning Fog Score	16.4	Words	132
Accuracy Score	0	SMOG Index	11.8	Complex words	25
Reading Age (years)	20.5	Coleman Liau Index	16.7	Percent of complex words	18.9
Flesch Kincaid Reading Ease	32.0	Automated Readability Index	15.6	Average words per sentence	22.0
Flesch Kincaid Grade Level	14.3	Sentences	6	Average syllables per word	1.8

Manuscript 26

Rincones O, Smith A', Chong P, Mancuso P, Wu VS, Sidhom M, Wong K, Ngo D, Gassner P, Girgis A. Encouraging Patients to Ask Questions: Development and Pilot Testing of a Question Prompt List for Patients Undergoing a Biopsy for Suspected Prostate Cancer. Curr Oncol. 2023 Feb 8;30(2):2088-2104. doi: 10.3390/curroncol30020162. PMID: 36826123; PMCID: PMC9954987.

Abstract

This study assessed the acceptability and feasibility of a question prompt list (QPL) to facilitate informed treatment decision-making in men with suspected localised prostate cancer, which involves values-based choices between options with similar efficacy but different side effects. The QPL was developed through iterative consultation with consumers, clinicians and researchers. Acceptability was assessed using study-specific questions regarding QPL satisfaction and usefulness and qualitative interviews. Feasibility was determined via the proportion of men given the QPL according to medical records and the completion of standardised measures of decisional outcomes. Quantitative data were analysed using descriptive and univariate statistics. Qualitative data were thematically analysed. Fifty-two men consented; 34 provided data for analysis. The QPL recipients reported moderate–high content satisfaction (70.6%) and perceived usefulness in guiding appointments when receiving biopsy results (64.7%). Two main qualitative themes also indicated the QPL acceptability: (1) the freedom to ask—acceptable timing, flexible usage and usefulness of the QPL, and (2) satisfaction with the QPL content. However, only 18.4% of eligible men received the QPL, indicating limited feasibility. The QPL is safe and acceptable, but further research is needed regarding how to facilitate the uptake of the question prompt list in clinical practice.

Keywords:

prostate biopsy; prostate cancer; question prompt list; treatment decision-making

Lay Summary (simple Prompt)

This study explored the development and testing of a Question Prompt List (QPL) to help men with suspected localized prostate cancer make informed decisions about their treatment. The QPL was designed to encourage patients to ask important questions about their diagnosis and treatment options, which often involve choices between options with similar effectiveness but different side effects. The study found that most men who received the QPL were satisfied with its content and found it useful during their medical appointments. However, only a small number of eligible men were actually given the QPL, suggesting challenges in implementing it widely in clinical practice. The study concludes that while the QPL is a helpful tool for patients, more research is needed to improve its use in healthcare settings.

Evaluation of the Lay Summary

**Assessment Category**	**Score**	**Assessment Category**	**Score**	**Assessment Category**	**Score**
Comprehension Score	5	Gunning Fog Score	16.1	Words	126
Accuracy Score	0	SMOG Index	11.3	Complex words	19
Reading Age (years)	20.5	Coleman Liau Index	15.8	Percent of complex words	15.1
Flesch Kincaid Reading Ease	40.3	Automated Readability Index	16.5	Average words per sentence	25.2
Flesch Kincaid Grade Level	13.9	Sentences	5	Average syllables per word	1.7

Lay Summary (extended Prompt)

This study developed a Question Prompt List (QPL) to help men with suspected localized prostate cancer ask important questions and make informed decisions about their treatment. The QPL was created with input from patients, doctors, and researchers, and it was tested to see if patients found it useful and easy to use. Out of 52 men who participated, most felt satisfied with the QPL's content and found it helpful in discussing biopsy results with their doctor. However, only a small number of eligible patients received the QPL, suggesting that more work is needed to make it widely available in clinical settings. Overall, the QPL was shown to be a helpful tool, but more research is needed to improve its use in practice.

Evaluation of the Lay Summary

Assessment Category	Score	Assessment Category	Score	Assessment Category	Score
Comprehension Score	5	Gunning Fog Score	15.0	Words	122
Accuracy Score	0	SMOG Index	10.4	Complex words	16
Reading Age (years)	18.5	Coleman Liau Index	12.5	Percent of complex words	13.1
Flesch Kincaid Reading Ease	51.0	Automated Readability Index	13.4	Average words per sentence	24.4
Flesch Kincaid Grade Level	12.2	Sentences	5	Average syllables per word	1.6

Manuscript 27

Yanagisawa T, Rajwa P, Kawada T, Bekku K, Laukhtina E, Deimling MV, Majdoub M, Chlosta M, Karakiewicz PI, Heidenreich A, Kimura T, Shariat SF. An Updated Systematic and Comprehensive Review of Cytoreductive Prostatectomy for Metastatic Prostate Cancer. Curr Oncol. 2023 Feb 10;30(2):2194-2216. doi: 10.3390/curroncol30020170. PMID: 36826131; PMCID: PMC9955685.

Abstract

(1) Background: Local therapy is highly promising in a multimodal approach strategy for patients with low-volume metastatic prostate cancer (mPCa). We aimed to systematically assess and summarize the safety, oncologic, and functional outcomes of cytoreductive prostatectomy (cRP) in mPCa. (2) Methods: Three databases were queried in September 2022 for publications that analyzed mPCa patients treated with cytoreductive prostatectomy without restrictions. The outcomes of interest were progression-free survival (PFS), cancer-specific survival (CSS), overall survival (OS), perioperative complication rates, and functional outcomes following cRP. (3) Results: Overall, 26 studies were included in this systematic review. Among eight population-based studies, cRP was associated with a reduced risk of CSS and OS compared with no local therapy (NLT) after adjusting for the effects of possible confounders. Furthermore, one population-based study showed that cRP reduced the risk of CSS even when compared with radiotherapy (RT) of the prostate after adjusting for the effects of possible confounders. In addition, one randomized controlled trial (RCT) demonstrated that local therapy (comprising 85% of cRP) significantly improved the prostate-specific antigen (PSA)-PFS and OS. Overall, cRP had acceptable perioperative complication rates and functional outcomes. (4) Conclusions: Mounting evidence suggests that cRP offers promising oncological and functional outcomes and technical feasibility and that it is associated with limited complications. Well-designed RCTs that limit selection bias in patients treated with cRP are warranted.

Keywords:

metastatic prostate cancer; prostatectomy; cytoreductive prostatectomy; local therapy

Lay Summary (simple Prompt)

This study reviewed current research on the effectiveness of cytoreductive prostatectomy (cRP), a surgical procedure to remove the prostate, in patients with metastatic prostate cancer that has spread but remains in low volume. The review included 26 studies and found that cRP could improve survival outcomes, such as progression-free survival and overall survival, compared to not having local therapy or even radiotherapy. Additionally, cRP showed acceptable levels of complications and good functional outcomes, making it a promising treatment option. The study suggests that cRP could be a valuable part of a multi-faceted approach to treating metastatic prostate cancer, but more rigorous clinical trials are needed to confirm these findings and better understand which patients will benefit the most.

Evaluation of the Lay Summary

**Assessment Category**	**Score**	**Assessment Category**	**Score**	**Assessment Category**	**Score**
Comprehension Score	5	Gunning Fog Score	20.7	Words	120
Accuracy Score	1	SMOG Index	14.7	Complex words	26
Reading Age (years)	23.5	Coleman Liau Index	17.2	Percent of complex words	21.7
Flesch Kincaid Reading Ease	17.8	Automated Readability Index	20.0	Average words per sentence	30.0
Flesch Kincaid Grade Level	18.2	Sentences	4	Average syllables per word	1.9

Lay Summary (extended Prompt)

This study reviews the use of cytoreductive prostatectomy (cRP) as a treatment for patients with metastatic prostate cancer. cRP involves surgically removing the prostate to reduce the overall tumor burden. The review looked at 26 studies to assess the safety, cancer outcomes, and complication rates of this approach. The results showed that patients who underwent cRP had better cancer-specific and overall survival compared to those who did not receive local therapy or were treated with radiotherapy. The procedure was also associated with manageable complication rates and good functional outcomes. These findings suggest that cRP could be a promising option for improving survival in patients with metastatic prostate cancer. However, more randomized controlled trials are needed to confirm these benefits and ensure patient selection is appropriate.

Evaluation of the Lay Summary

**Assessment Category**	**Score**	**Assessment Category**	**Score**	**Assessment Category**	**Score**
Comprehension Score	5	Gunning Fog Score	16.4	Words	126
Accuracy Score	0	SMOG Index	11.8	Complex words	29
Reading Age (years)	20.5	Coleman Liau Index	17.7	Percent of complex words	23.0
Flesch Kincaid Reading Ease	30.8	Automated Readability Index	14.4	Average words per sentence	18.0
Flesch Kincaid Grade Level	13.4	Sentences	7	Average syllables per word	1.9

Manuscript 28

Prata F, Anceschi U, Cordelli E, Faiella E, Civitella A, Tuzzolo P, Iannuzzi A, Ragusa A, Esperto F, Prata SM, Sicilia R, Muto G, Grasso RF, Scarpa RM, Soda P, Simone G, Papalia R. Radiomic Machine-Learning Analysis of Multiparametric Magnetic Resonance Imaging in the Diagnosis of Clinically Significant Prostate Cancer: New Combination of Textural and Clinical Features. Curr Oncol. 2023 Feb 7;30(2):2021-2031. doi: 10.3390/curroncol30020157. PMID: 36826118; PMCID: PMC9955797.

Abstract

Background: The aim of our study was to develop a radiomic tool for the prediction of clinically significant prostate cancer. Methods: From September 2020 to December 2021, 91 patients who underwent magnetic resonance imaging prostate fusion biopsy at our institution were selected. Prostate cancer aggressiveness was assessed by combining the three orthogonal planes-Llocal binary pattern the 3Dgray level co-occurrence matrix, and other first order statistical features with clinical (semantic) features. The 487 features were used to predict whether the Gleason score was clinically significant (≥7) in the final pathology. A feature selection algorithm was used to determine the most predictive features, and at the end of the process, nine features were chosen through a 10-fold cross validation. Results: The feature analysis revealed a detection accuracy of 83.5%, with a clinically significant precision of 84.4% and a clinically significant sensitivity of 91.5%. The resulting area under the curve was 80.4%. Conclusions: Radiomic analysis allowed us to develop a tool that was able to predict a Gleason score of ≥7. This new tool may improve the detection rate of clinically significant prostate cancer and overcome the limitations of the subjective interpretation of magnetic resonance imaging, reducing the number of useless biopsies.

Keywords:

clinically significant; machine-learning; prostate biopsy; prostate cancer; radiomic

Lay Summary (simple Prompt)

This study aimed to develop a new tool using advanced machine-learning techniques to better detect clinically significant prostate cancer. Researchers analyzed MRI scans and combined detailed image features with clinical information to predict the aggressiveness of prostate cancer, specifically focusing on cases with a Gleason score of 7 or higher, which indicates more serious disease. By using a feature selection process and validating the tool, the study achieved high accuracy (83.5%) and sensitivity (91.5%) in predicting significant prostate cancer. This approach could improve the accuracy of prostate cancer diagnoses, reduce the reliance on subjective MRI interpretations, and decrease the number of unnecessary biopsies, ultimately leading to more efficient and effective patient care.

Evaluation of the Lay Summary

**Assessment Category**	**Score**	**Assessment Category**	**Score**	**Assessment Category**	**Score**
Comprehension Score	5	Gunning Fog Score	17.8	Words	115
Accuracy Score	1	SMOG Index	12.7	Complex words	29
Reading Age (years)	21.5	Coleman Liau Index	18.0	Percent of complex words	25.2
Flesch Kincaid Reading Ease	24.8	Automated Readability Index	15.2	Average words per sentence	19.2
Flesch Kincaid Grade Level	14.6	Sentences	6	Average syllables per word	1.9

Lay Summary (extended Prompt)

This study aimed to create a tool using machine learning and radiomic analysis to better predict clinically significant prostate cancer, which is more aggressive and requires treatment. Researchers analyzed MRI scans of 91 patients and extracted hundreds of features from the images, focusing on specific patterns and textures. They combined this data with clinical information to predict if the cancer had a Gleason score of 7 or higher, indicating a more serious form of the disease. The tool demonstrated an accuracy of 83.5%, with a high sensitivity for detecting aggressive cancer. This new method may help doctors more accurately identify dangerous prostate cancer and reduce the need for unnecessary biopsies, improving patient care.

Evaluation of the Lay Summary

**Assessment Category**	**Score**	**Assessment Category**	**Score**	**Assessment Category**	**Score**
Comprehension Score	5	Gunning Fog Score	16.4	Words	114
Accuracy Score	1	SMOG Index	11.8	Complex words	25
Reading Age (years)	19.5	Coleman Liau Index	15.7	Percent of complex words	21.9
Flesch Kincaid Reading Ease	35.4	Automated Readability Index	13.3	Average words per sentence	19.0
Flesch Kincaid Grade Level	13.0	Sentences	6	Average syllables per word	1.8

Manuscript 29

Berenguer CV, Pereira F, Câmara JS, Pereira JAM. Underlying Features of Prostate Cancer-Statistics, Risk Factors, and Emerging Methods for Its Diagnosis. Curr Oncol. 2023 Feb 15;30(2):2300-2321. doi: 10.3390/curroncol30020178. PMID: 36826139; PMCID: PMC9955741.

Abstract

Prostate cancer (PCa) is the most frequently occurring type of malignant tumor and a leading cause of oncological death in men. PCa is very heterogeneous in terms of grade, phenotypes, and genetics, displaying complex features. This tumor often has indolent growth, not compromising the patient’s quality of life, while its more aggressive forms can manifest rapid growth with progression to adjacent organs and spread to lymph nodes and bones. Nevertheless, the overtreatment of PCa patients leads to important physical, mental, and economic burdens, which can be avoided with careful monitoring. Early detection, even in the cases of locally advanced and metastatic tumors, provides a higher chance of cure, and patients can thus go through less aggressive treatments with fewer side effects. Furthermore, it is important to offer knowledge about how modifiable risk factors can be an effective method for reducing cancer risk. Innovations in PCa diagnostics and therapy are still required to overcome some of the limitations of the current screening techniques, in terms of specificity and sensitivity. In this context, this review provides a brief overview of PCa statistics, reporting its incidence and mortality rates worldwide, risk factors, and emerging screening strategies.

Keywords:

prostate cancer; incidence; mortality; risk factors; biomarkers

Lay Summary (simple Prompt)

Prostate cancer is the most common cancer in men and a leading cause of cancer-related deaths. It can range from slow-growing types that do not affect quality of life to aggressive forms that spread quickly to other organs. This study reviews important information about prostate cancer, including its rates of occurrence and death worldwide, risk factors, and new methods for early detection and diagnosis. Early detection of prostate cancer is crucial as it allows for less aggressive treatments with fewer side effects, improving the chances of a cure. The study also discusses how understanding and addressing risk factors, such as lifestyle changes, can help reduce the risk of developing prostate cancer. There is an ongoing need for improved diagnostic tools to better distinguish between different types of prostate cancer and to ensure appropriate treatment, avoiding unnecessary physical, mental, and economic burdens on patients.

Evaluation of the Lay Summary

Assessment Category	Score	Assessment Category	Score	Assessment Category	Score
Comprehension Score	5	Gunning Fog Score	16.8	Words	145
Accuracy Score	0	SMOG Index	12.0	Complex words	26
Reading Age (years)	20.5	Coleman Liau Index	15.9	Percent of complex words	17.9
Flesch Kincaid Reading Ease	37.6	Automated Readability Index	16.0	Average words per sentence	24.2
Flesch Kincaid Grade Level	14.0	Sentences	6	Average syllables per word	1.7

Lay Summary (extended Prompt)

Prostate cancer (PCa) is the most common type of cancer in men and a major cause of cancer-related deaths. It can vary greatly in how it behaves, with some tumors growing slowly and others spreading quickly to nearby organs and bones. Detecting prostate cancer early improves the chances of successful treatment and helps reduce the need for aggressive therapies, which can have significant physical, emotional, and financial impacts. This study looks at the latest statistics on prostate cancer, the factors that increase the risk of developing it, and new screening methods that could help catch the disease earlier. Improving these diagnostic tools can help doctors better identify aggressive forms of prostate cancer and reduce unnecessary treatments for those with less severe cases. This research highlights the importance of ongoing innovations in prostate cancer detection and treatment to improve patient outcomes.

Evaluation of the Lay Summary

**Assessment Category**	**Score**	**Assessment Category**	**Score**	**Assessment Category**	**Score**
Comprehension Score	5	Gunning Fog Score	15.4	Words	141
Accuracy Score	0	SMOG Index	10.8	Complex words	21
Reading Age (years)	19.5	Coleman Liau Index	16.3	Percent of complex words	14.9
Flesch Kincaid Reading Ease	40.8	Automated Readability Index	16.0	Average words per sentence	23.5
Flesch Kincaid Grade Level	13.4	Sentences	6	Average syllables per word	1.7

Manuscript 30

Takeuchi S, Iinuma K, Nakane K, Nakano M, Kawase M, Kawase K, Takai M, Kato D, Mori T, Takano H, Kumano T, Matsuo M, Koie T. Direct Comparison of Two Different Definitions with Biochemical Recurrence after Low-Dose-Rate Brachytherapy for Prostate Cancer. Curr Oncol. 2023 Feb 26;30(3):2792-2800. doi: 10.3390/curroncol30030212. PMID: 36975425; PMCID: PMC10047870.

Abstract

We aimed to determine whether biochemical recurrence-free survival (BRFS) of patients with prostate cancer (PCa) who received low-dose-rate brachytherapy (LDR-BT) differed according to the definition of biochemical recurrence (BCR) after radical prostatectomy (RP) and the definition given by the Japanese Prostate Cancer Outcome Study of Permanent Iodine-125 Seed Implantation (J-POPS). We reviewed the clinical records of 476 consecutive patients with PCa who received LDR-BT at the Gifu University Hospital. The primary endpoint of this study was the difference in BRFS between the two aforementioned definitions. When the follow-up period ended, 74 (15.5%) and 20 (4.2%) patients had BCR according to the RP and J-POPS definitions, respectively. The 5-year BRFS rates were 85.0% and 96.9% for the RP and J-POPS definitions, respectively ($p < 0.005$). According to the RP definition, the 5-year BRFS rates were 80.6% in the group aged <63 years and 86.6% in those aged ≥63 years ($p = 0.050$). According to the J-POPS definition, the 5-year BRFS rates were 94.1% and 97.8% in the groups aged <63 years and ≥63 years, respectively ($p = 0.005$). The definition of recurrence in LDR-BT may need to be reconsidered.

Keywords:

prostate cancer; low-dose-rate brachytherapy; biochemical recurrence; definition of biochemical recurrence

Lay Summary (simple Prompt)

This study investigates whether different definitions of biochemical recurrence (BCR) affect the measurement of recurrence-free survival in prostate cancer patients treated with low-dose-rate brachytherapy (LDR-BT). Researchers compared two definitions: one used after radical prostatectomy and another from the Japanese Prostate Cancer Outcome Study of Permanent Iodine-125 Seed Implantation (J-POPS). Analyzing data from 476 patients, they found that the 5-year recurrence-free survival rate was significantly higher using the J-POPS definition (96.9%) compared to the radical prostatectomy definition (85.0%). Additionally, younger patients (<63 years) showed lower recurrence-free rates than older patients (≥63 years) under both definitions, but the difference was more pronounced using the J-POPS criteria. The findings suggest that the way recurrence is defined can significantly impact the reported success rates of LDR-BT, indicating a need to standardize definitions to better evaluate patient outcomes.

Evaluation of the Lay Summary

**Assessment Category**	**Score**	**Assessment Category**	**Score**	**Assessment Category**	**Score**
Comprehension Score	5	Gunning Fog Score	15.7	Words	91
Accuracy Score	1	SMOG Index	12.3	Complex words	27
Reading Age (years)	20.5	Coleman Liau Index	18.0	Percent of complex words	29.7
Flesch Kincaid Reading Ease	19.5	Automated Readability Index	13.2	Average words per sentence	15.2
Flesch Kincaid Grade Level	14.3	Sentences	6	Average syllables per word	2.0

Lay Summary (extended Prompt)

This study looked at how two different ways of defining biochemical recurrence (BCR) after low-dose-rate brachytherapy (LDR-BT) for prostate cancer affect the measurement of patients' recurrence-free survival rates. The researchers compared the definitions used after radical prostatectomy (RP) and by the Japanese Prostate Cancer Outcome Study (J-POPS). They reviewed the records of 476 prostate cancer patients treated with LDR-BT. They found that the percentage of patients without cancer recurrence after five years was higher when using the J-POPS definition (96.9%) compared to the RP definition (85.0%). The study suggests that the way recurrence is defined can significantly impact the reported outcomes and that the current definitions might need to be reconsidered to ensure consistency and accuracy in assessing patient progress after treatment.

Evaluation of the Lay Summary

**Assessment Category**	**Score**	**Assessment Category**	**Score**	**Assessment Category**	**Score**
Comprehension Score	5	Gunning Fog Score	14.5	Words	131
Accuracy Score	0	SMOG Index	10.7	Complex words	24
Reading Age (years)	18.5	Coleman Liau Index	15.5	Percent of complex words	18.3
Flesch Kincaid Reading Ease	38.0	Automated Readability Index	13.0	Average words per sentence	18.7
Flesch Kincaid Grade Level	12.6	Sentences	7	Average syllables per word	1.8

Manuscript 31

Lima T, Macedo-Silva C, Felizardo D, Fraga J, Carneiro I, Jerónimo C, Henrique R, Fardilha M, Vitorino R. Gal-3 Protein Expression and Localization in Prostate Tumours. Curr Oncol. 2023 Feb 23;30(3):2729-2742. doi: 10.3390/curroncol30030206. PMID: 36975419; PMCID: PMC10047320.

Abstract:

Gal-3 plays an important role in cell survival, mRNA splicing, and cell–cell and cell–matrix interactions. Depending on its cellular localization and cancer type, Gal-3 may have tumour-suppressive or tumour-promoting activities. Given the promising diagnostic role of Gal-3 in the urine of PCa patients found in our previous study, its concordant gene and protein expression levels, and its involvement in PCa-related biological processes (e.g., morphogenesis of the prostate gland epithelium), we aimed to investigate this protein immunohistochemically in tumour and normal prostate tissues. Gal-3 protein expression was evaluated in 48 tumour prostate tissues, eight normal prostate tissues and 14 adjacent-normal prostate tissues. Decreased Gal-3 staining was detected in tumour tissues compared with normal tissues. Although Gal-3 staining was decreased in tumour tissues with GS 5-8 and pT2 and pT3 stages compared with normal prostate tissue, no correlation was found between Gal-3 expression and PCa progression. In the present study, the pattern of cellular localization differed between groups, as Gal-3 was predominantly excluded from the nucleus in tumour tissues. Furthermore, Gal-3 had no significant effect on survival and relapse in these PCa patients. This work confirms Gal-3 as a promising marker for PCa diagnosis.

Keywords:

Galectin-3; prostate tissue; marker; prostate cancer; localization; patient survival

Lay Summary (simple Prompt)

This study explores the role of Galectin-3 (Gal-3), a protein involved in various cellular processes, in prostate cancer. The researchers examined Gal-3 levels in tumor and normal prostate tissues to determine if this protein could help diagnose prostate cancer. They found that Gal-3 expression was lower in prostate cancer tissues compared to normal tissues, but there was no clear link between Gal-3 levels and cancer progression or patient survival. Interestingly, the location of Gal-3 within the cells differed between cancerous and normal tissues, with the protein mostly absent from the nucleus in cancer cells. Although Gal-3 does not seem to impact cancer progression or survival, it may still serve as a useful marker for diagnosing prostate cancer. This study supports the potential of Gal-3 in improving the early detection of prostate cancer, which could lead to better patient outcomes.

Evaluation of the Lay Summary

**Assessment Category**	**Score**	**Assessment Category**	**Score**	**Assessment Category**	**Score**
Comprehension Score	5	Gunning Fog Score	15.0	Words	147
Accuracy Score	0	SMOG Index	10.6	Complex words	20
Reading Age (years)	18.5	Coleman Liau Index	13.5	Percent of complex words	13.6
Flesch Kincaid Reading Ease	45.6	Automated Readability Index	14.3	Average words per sentence	24.5
Flesch Kincaid Grade Level	13.0	Sentences	6	Average syllables per word	1.6

Lay Summary (extended Prompt)

This study looked at the role of a protein called Gal-3 in prostate cancer. Gal-3 is involved in many cell processes, like cell survival and how cells interact with each other. The researchers wanted to understand how Gal-3 is expressed and located in cancerous and normal prostate tissues. They found that Gal-3 levels were lower in cancer tissues compared to normal tissues. Additionally, in cancer tissues, Gal-3 was mostly found outside the cell nucleus, which was different from normal tissues. The study did not find a clear link between Gal-3 levels and the progression of prostate cancer or patient survival. However, the results suggest that Gal-3 could still be a useful marker for diagnosing prostate cancer. This research adds to our understanding of how Gal-3 may be involved in prostate cancer and its potential role in diagnosis.

Evaluation of the Lay Summary

**Assessment Category**	**Score**	**Assessment Category**	**Score**	**Assessment Category**	**Score**
Comprehension Score	5	Gunning Fog Score	11.7	Words	145
Accuracy Score	0	SMOG Index	8.3	Complex words	16
Reading Age (years)	15.5	Coleman Liau Index	11.8	Percent of complex words	11.0
Flesch Kincaid Reading Ease	58.9	Automated Readability Index	9.7	Average words per sentence	18.1
Flesch Kincaid Grade Level	9.5	Sentences	8	Average syllables per word	1.5

Manuscript 32

Lambert E, Allaeys C, Berquin C, De Visschere P, Verbeke S, Vanneste B, Fonteyne V, Van Praet C, Lumen N. Is It Safe to Switch from a Standard Anterior to Retzius-Sparing Approach in Robot-Assisted Radical Prostatectomy? Curr Oncol. 2023 Mar 17;30(3):3447-3460. doi: 10.3390/curroncol30030261. PMID: 36975474; PMCID: PMC10047447.

Abstract:

Background: Retzius-sparing robot-assisted radical prostatectomy (RS-RARP) has been shown to lead to better outcomes regarding early continence compared to standard anterior RARP (SA-RARP). The goal of this study was to assess the feasibility and safety of implementing RS-RARP in a tertiary center with experience in SA-RARP. Methods: From February 2020, all newly diagnosed non-metastatic prostate cancer patients for whom RARP was indicated were evaluated for RS-RARP. Data from the first 100 RS-RARP patients were prospectively collected and compared with data from the last 100 SA-RARP patients. Patients were evaluated for Clavien Dindo grade ≥3a complications, urinary continence after 2 and 6 weeks, 3, 6 and 12 months, erectile function, positive surgical margins (PSMs) and biochemical recurrence (BCR). Results: There was no significant difference in postoperative complications at Clavien-Dindo grade ≥3a (SA-RARP: 6, RS-RARP: 4; p = 0.292). At all time points, significantly higher proportions of RS-RARP patients were continent (p < 0.001). No significant differences in postoperative potency were observed (52% vs. 59%, respectively, p = 0.608). PSMs were more frequent in the RS-RARP group (43% vs. 29%, p = 0.034), especially in locally advanced tumors (pT3: 64.6% vs. 43.8%, p = 0.041—pT2: 23.5% vs. 15.4%, p = 0.329). The one-year BCR-free survival was 82.6% vs. 81.6% in the SA-RARP and RS-RARP groups, respectively (p = 0.567). The median follow-up was 22 [18–27] vs. 24.5 [17–35] months in the RS-RARP and SA-RARP groups, respectively (p = 0.008). Conclusions: The transition from SA-RARP to RS-RARP can be safely performed by surgeons proficient in SA-RARP. Continence results after RS-RARP were significantly better at any time point. A higher proportion of PSMs was observed, although it did not result in a worse BCR-free survival.

Keywords:

radical prostatectomy; Retzius-sparing; urinary continence; erectile dysfunction; biochemical recurrence; robotic surgery

Lay Summary (simple Prompt)

This study evaluates whether it is safe for surgeons to switch from the standard approach to a newer technique, called Retzius-sparing, in robot-assisted surgery to remove the prostate in men with non-metastatic prostate cancer. The Retzius-sparing approach has been shown to improve early urinary continence after surgery. The study compared outcomes for the first 100 patients who underwent this newer technique with the last 100 patients who had the standard surgery. Results showed that the newer technique led to better urinary continence without increasing serious complications. However, there was a higher rate of positive surgical margins, meaning some cancerous tissue was left behind, especially in more advanced cases. Despite this, both groups had similar survival rates without cancer recurrence. These findings suggest that experienced surgeons can safely adopt the new technique, offering benefits in urinary control after surgery.

Evaluation of the Lay Summary

**Assessment Category**	**Score**	**Assessment Category**	**Score**	**Assessment Category**	**Score**
Comprehension Score	5	Gunning Fog Score	16.6	Words	142
Accuracy Score	0	SMOG Index	12.4	Complex words	32
Reading Age (years)	20.5	Coleman Liau Index	16.8	Percent of complex words	22.5
Flesch Kincaid Reading Ease	33.7	Automated Readability Index	14.8	Average words per sentence	20.3
Flesch Kincaid Grade Level	13.6	Sentences	7	Average syllables per word	1.8

Lay Summary (extended Prompt)

This study looked at a different way to perform robotic surgery for prostate cancer, called Retzius-sparing robot-assisted radical prostatectomy (RS-RARP), to see if it is as safe and effective as the standard method (SA-RARP). The researchers compared 100 patients who had the new RS-RARP method with 100 patients who had the standard surgery. They found that RS-RARP patients regained urinary control faster and more often than those who had the standard surgery. However, there was a slightly higher chance of leaving some cancer cells behind in the RS-RARP group, but this did not affect their overall cancer-free survival rates after one year. The study suggests that experienced surgeons can safely switch to this new technique, which may improve recovery without increasing serious complications.

Evaluation of the Lay Summary

**Assessment Category**	**Score**	**Assessment Category**	**Score**	**Assessment Category**	**Score**
Comprehension Score	5	Gunning Fog Score	16.3	Words	131
Accuracy Score	0	SMOG Index	11.6	Complex words	20
Reading Age (years)	19.5	Coleman Liau Index	13.5	Percent of complex words	15.3
Flesch Kincaid Reading Ease	44.6	Automated Readability Index	15.1	Average words per sentence	26.2
Flesch Kincaid Grade Level	13.5	Sentences	5	Average syllables per word	1.6

Manuscript 33

Zhang W, Guh DP, Mohammadi T, Pataky RE, Tam ACT, Lynd LD, Conklin AI. Health Care Costs Attributable to Prostate Cancer in British Columbia, Canada: A Population-Based Cohort Study. Curr Oncol. 2023 Mar 8;30(3):3176-3188. doi: 10.3390/curroncol30030240. PMID: 36975453; PMCID: PMC10047657.

Abstract

We aimed to estimate the total health care costs attributable to prostate cancer (PCa) during care phases by age, cancer stage, tumor grade, and primary treatment in the first year in British Columbia (BC), Canada. Using linked administrative health data, we followed a cohort of men aged ≥ 50 years at diagnosis with PCa between 2010 and 2017 (Cohort 1) from the diagnosis date until the date of death, the last date of observation, or 31 December 2019. Patients who died from PCa after 1 January 2010, were selected for Cohort 2. PCa attributable costs were estimated by comparing costs in patients to matched controls. Cohort 1 (n = 22,672) had a mean age of 69.9 years (SD = 8.9) and a median follow-up time of 5.2 years. Cohort 2 included 6942 patients. Mean PCa attributable costs were the highest during the first year after diagnosis ($14,307.9 [95% CI: $13,970.0, $14,645.8]) and the year before death ($9959.7 [$8738.8, $11,181.0]). Primary treatment with radiation therapy had significantly higher costs each year after diagnosis than a radical prostatectomy or other surgeries in advanced-stage PCa. Androgen deprivation therapy (and/or chemotherapy) had the highest cost for high-grade and early-stage cancer during the three years after diagnosis. No treatment group had the lowest cost. Updated cost estimates could inform economic evaluations and decision-making.

Keywords:

prostate cancer; healthcare costs; cost evaluation; administrative data

Lay Summary (simple Prompt)

This study examines the healthcare costs associated with prostate cancer in British Columbia, Canada, focusing on different stages of care, patient age, cancer stage, tumor grade, and primary treatment methods. By analyzing data from over 22,000 men diagnosed with prostate cancer between 2010 and 2017, the study found that healthcare costs were highest in the first year following diagnosis and in the year before death. Treatments like radiation therapy and androgen deprivation therapy (with or without chemotherapy) were associated with higher costs, especially for patients with advanced or high-grade cancer. Understanding these cost patterns can help inform healthcare planning and economic decision-making, ensuring that resources are allocated effectively to manage prostate cancer care and support patients throughout their treatment journey.

Evaluation of the Lay Summary

**Assessment Category**	**Score**	**Assessment Category**	**Score**	**Assessment Category**	**Score**
Comprehension Score	3	Gunning Fog Score	19.8	Words	123
Accuracy Score	1	SMOG Index	14.4	Complex words	25
Reading Age (years)	23.5	Coleman Liau Index	17.9	Percent of complex words	20.3
Flesch Kincaid Reading Ease	20.9	Automated Readability Index	20.9	Average words per sentence	30.8
Flesch Kincaid Grade Level	18.0	Sentences	4	Average syllables per word	1.8

Lay Summary (extended Prompt)

This study examined the healthcare costs associated with prostate cancer in British Columbia, Canada, focusing on different stages of care, patient age, cancer stage, and treatment types. By analyzing data from men diagnosed with prostate cancer between 2010 and 2017, the researchers estimated the costs related to prostate cancer by comparing patients to similar men without cancer. They found that the highest costs occurred in the first year after diagnosis and in the year before death. Treatments such as radiation therapy and androgen deprivation therapy were more expensive, especially for advanced or high-grade cancers. Understanding these costs can help healthcare providers and policymakers make better decisions about prostate cancer care and resource allocation, potentially leading to more cost-effective treatment strategies.

Evaluation of the Lay Summary

**Assessment Category**	**Score**	**Assessment Category**	**Score**	**Assessment Category**	**Score**
Comprehension Score	3	Gunning Fog Score	18.6	Words	122
Accuracy Score	1	SMOG Index	13.9	Complex words	29
Reading Age (years)	22.5	Coleman Liau Index	17.9	Percent of complex words	23.8
Flesch Kincaid Reading Ease	26.7	Automated Readability Index	17.8	Average words per sentence	24.4
Flesch Kincaid Grade Level	15.6	Sentences	5	Average syllables per word	1.8

Manuscript 34

Moshref L, Abidullah M, Czaykowski P, Chowdhury A, Wightman R, Hebbard P. Prostate Cancer Metastasis to Stomach: A Case Report and Review of Literature. Curr Oncol. 2023 Mar 30;30(4):3901-3914. doi: 10.3390/curroncol30040295. PMID: 37185408; PMCID: PMC10137262.

Abstract

Metastatic prostate cancer is a common diagnosis with a protracted but terminal course. Gastrointestinal (GI) tract involvement is extremely rare, and reportedly portends a poor prognosis. It can present years after the initial prostate cancer diagnosis. Only fifteen cases of prostate cancer metastasis to the stomach have been reported in the literature. We report a case of a 72-year-old man with metastatic castration-resistant prostate cancer and extensive bony involvement. He presented a decade after the diagnosis of prostate cancer with signs of heartburn; a gastric biopsy was initially believed to represent primary gastric carcinoma, but subsequently a diagnosis of prostate cancer metastatic to the stomach was confirmed. This case highlights the importance of the provision of a pertinent clinical history and clinical differential diagnosis at the time of submission of surgical pathology specimens, as well as highlighting the need to have a low index of suspicion to pursue additional pathologic markers whenever a presumed second adenocarcinoma is noted in the context of a patient having a history of current or prior advanced-stage adenocarcinoma of another site. The correct diagnosis can shield the patient from the morbidity of inappropriate surgical or medical management.

Keywords:

prostate-specific antigen (PSA); gastrointestinal tract (GI tract); immunohistochemistry (IHC)

Lay Summary (simple Prompt)

This study presents a rare case of prostate cancer spreading to the stomach, which is an uncommon and serious condition. The report discusses a 72-year-old man who, ten years after his initial diagnosis of advanced prostate cancer, developed symptoms that suggested stomach cancer. Further investigation revealed that the cancer in his stomach was actually a metastasis from his prostate cancer. This case emphasizes the importance of sharing a patient’s full medical history with pathologists and considering all possibilities when a new cancer is suspected in a patient with a history of another type of cancer. Correctly identifying the origin of metastatic cancer is crucial to avoid unnecessary treatments and to manage the patient's condition more effectively. The findings from this study could help healthcare professionals better diagnose and treat rare cancer metastases, improving patient outcomes.

Evaluation of the Lay Summary

**Assessment Category**	**Score**	**Assessment Category**	**Score**	**Assessment Category**	**Score**
Comprehension Score	4	Gunning Fog Score	18.5	Words	137
Accuracy Score	1	SMOG Index	13.3	Complex words	32
Reading Age (years)	21.5	Coleman Liau Index	16.8	Percent of complex words	23.4
Flesch Kincaid Reading Ease	29.3	Automated Readability Index	16.1	Average words per sentence	23.8
Flesch Kincaid Grade Level	14.8	Sentences	6	Average syllables per word	1.8

Lay Summary (extended Prompt)

This study reports a rare case of prostate cancer spreading to the stomach, which is unusual and typically signals a poor outcome. A 72-year-old man with advanced prostate cancer, originally diagnosed a decade earlier, developed stomach problems that were initially thought to be a new stomach cancer. However, further tests confirmed that the cancer in his stomach was actually from his prostate. This case shows the importance of doctors sharing a patient's full medical history and considering all possibilities when examining new tumors in patients with a history of cancer. Recognizing this rare spread of prostate cancer can help avoid unnecessary and potentially harmful treatments. This study emphasizes the need for careful diagnosis and highlights that prostate cancer can rarely spread to unusual places like the stomach, even many years after the initial diagnosis.

Evaluation of the Lay Summary

**Assessment Category**	**Score**	**Assessment Category**	**Score**	**Assessment Category**	**Score**
Comprehension Score	4	Gunning Fog Score	16.1	Words	136
Accuracy Score	0	SMOG Index	11.6	Complex words	24
Reading Age (years)	20.5	Coleman Liau Index	15.7	Percent of complex words	17.7
Flesch Kincaid Reading Ease	34.5	Automated Readability Index	15.1	Average words per sentence	22.7
Flesch Kincaid Grade Level	14.1	Sentences	6	Average syllables per word	1.8

Manuscript 35

Téllez C, Szczesniewski J, Virseda-Chamorro M, Arance I, Angulo JC. Update on Adjustable Trans-Obturator Male System (ATOMS) for Male Incontinence after Prostate Cancer Surgery. Curr Oncol. 2023 Apr 12;30(4):4153-4165. doi: 10.3390/curroncol30040316. PMID: 37185429; PMCID: PMC10136884.

Abstract

(1) Background: The adjustable trans-obturator male system (ATOMS) is a surgical device developed to treat post-prostatectomy incontinence (PPI) after prostate cancer treatment. We review the current literature on this anti-incontinence device with the intention of assessing the effectiveness, safety and duration of the silicone-covered scrotal port (SSP) ATOMS, the only generation of the device that is currently available. (2) Material and Methods: Non-systematic literature review is performed. Forty-eight full-text articles are assessed for eligibility. Case reports, expert opinions or commentaries without specific data reported (n = 6), studies with patients who underwent intervention before 2014 (IP or SP ATOMS; n = 10), and studies with incontinence after transurethral resection of the prostate (TUR-P; n = 2) are excluded for analysis. Thirty studies with SSP ATOMS are included in a qualitative synthesis that incorporates systematic reviews (n = 3), articles partially overlapping with other previously published studies (e.g., follow-up or series updates; n = 9), and studies focusing on specific populations (n = 8). Only articles revealing outcomes of SSP ATOMS were included in the quantitative synthesis of results (n = 10). (3) Results: the pooled data of 1515 patients from the 10 studies with SSP ATOMS confirmed very satisfactory results with this device after adjustment: dry rate: 63–82%, improved rate: 85–100%, complication rate: 7–33%, device infection rate: 2.7–6.2% and explant rate: 0–19%. The durability of the device is reassuring, with 89% of devices in place 5 years after implantation. (4) Conclusion: Despite the absence of randomized controlled studies, the literature findings confirm results of SSP ATOMS appear equivalent to those of artificial urinary sphincters (AUSs) in terms of continence, satisfaction and complications, but with a lower rate of revision in the long-term. A prospective study identified that patients with daily pad test results <900 mL and a Male Stress Incontinence Grading Scale (MSIGS) of not 4 (i.e., early and persistent stream or urine loss) are the best candidates. Future studies centered on the elder population at

higher risk of impaired cognitive ability and in patients including radiation as prostate cancer treatment are needed.

Keywords:
adjustable trans-obturator male system; effectiveness; safety; post-prostatectomy incontinence; artificial urinary sphincter; prostate cancer

Lay Summary (simple Prompt)

This study reviews the effectiveness and safety of the adjustable trans-obturator male system (ATOMS), a device designed to treat urinary incontinence in men following prostate cancer surgery. The analysis included data from 1,515 patients across multiple studies, showing that ATOMS provides good results: 63-82% of patients became completely dry, and 85-100% showed improvement after adjustment. The device had a low complication rate (7-33%), with only a small percentage requiring removal due to infection or other issues. The device's durability is promising, with 89% of devices still in use five years after implantation. Although there are no randomized controlled trials, current evidence suggests that ATOMS is as effective as the more commonly used artificial urinary sphincter but may require fewer adjustments over time. Further research is needed, particularly for older patients and those who have had radiation therapy as part of their prostate cancer treatment.

Evaluation of the Lay Summary

**Assessment Category**	**Score**	**Assessment Category**	**Score**	**Assessment Category**	**Score**
Comprehension Score	5	Gunning Fog Score	18.3	Words	149
Accuracy Score	1	SMOG Index	13.1	Complex words	31
Reading Age (years)	21.5	Coleman Liau Index	15.4	Percent of complex words	20.8
Flesch Kincaid Reading Ease	28.9	Automated Readability Index	15.9	Average words per sentence	24.8
Flesch Kincaid Grade Level	15.4	Sentences	6	Average syllables per word	1.8

Lay Summary (extended Prompt)

This study reviews the effectiveness and safety of the Adjustable Trans-Obturator Male System (ATOMS), a device used to treat urinary incontinence in men after prostate cancer surgery. The researchers looked at data from 1,515 patients across several studies. They found that the ATOMS device has high success rates, with 63–82% of patients becoming dry and 85–100% experiencing improvement. Complication rates were relatively low, and the device remained in place for five years in 89% of cases. The results suggest that the ATOMS device works as well as the more commonly used artificial urinary sphincters but requires fewer follow-up surgeries. The study highlights that certain patients, especially those with less severe incontinence, are better candidates for this device. Future research is needed to understand its effectiveness in older patients and those who have had radiation treatment for prostate cancer.

Evaluation of the Lay Summary

Assessment Category	Score	Assessment Category	Score	Assessment Category	Score
Comprehension Score	5	Gunning Fog Score	14.3	Words	141
Accuracy Score	0	SMOG Index	10.7	Complex words	24
Reading Age (years)	18.5	Coleman Liau Index	15.3	Percent of complex words	17.0
Flesch Kincaid Reading Ease	41.8	Automated Readability Index	13.5	Average words per sentence	20.1
Flesch Kincaid Grade Level	12.4	Sentences	7	Average syllables per word	1.7

Manuscript 36

Yamaguchi T, Matsuo M, Mori T, Noda Y, Makita C, Hyodo F, Iinuma K, Nakano M, Koie T, Tanaka H. Seed Density as a New Predictive Index of Seed Migration in Brachytherapy for Prostate Cancer Using Iodine-125 Loose Seed. Curr Oncol. 2023 Apr 4;30(4):4060-4066. doi: 10.3390/curroncol30040308. PMID: 37185421; PMCID: PMC10136498.

Abstract

Aim: This study aimed to examine the usefulness of seed density as a predictor of seed migration in patients with prostate cancer who received brachytherapy using Iodine-125 loose seed. Methods: From May 2006 to April 2016, 320 patients with localized prostate cancer underwent transperineal brachytherapy using iodine-125 loose seeds. Among them, 202 (63.1%) patients received brachytherapy monotherapy and 118 (36.9%) received combined brachytherapy and external beam radiotherapy. Seed density was calculated using the following formula: seed density = implanted seed number/prostate volume. All patients underwent radiography of the chest, abdomen and pelvis, and computed tomography at 1 day, 1 month, and 1 year after brachytherapy to evaluate the presence of seed migration. Results: In total, the number of implanted seeds was 21,876. Seed migration was detected in 92 (28.8%) patients. Of a total of 21,876 seeds, 144 (0.66%) showed migration. The number of needles, number of seeds, and seed density were significantly higher in the group with migration than in the group without migration ($p = 0.05$). The ROC cutoff values for prostate volume, number of needles, number of seeds, and seed density were 20.9 cc, 21, 65, and 3.0, respectively. In the univariate analysis, prostate volume, number of needles, number of seeds, seed density, and treatment modality were all significant factors in predicting migration ($p = 0.05$). In the multivariate analysis, seed density and treatment modality were significant factors in predicting migration ($p = 0.05$). Conclusion: Seed density is useful for predicting seed migration. In cases with seed density > 3.0, it is necessary to take measures such as considering the use of stranded seeds.

Keywords:

prostate cancer; brachytherapy; seed migration; low dose rate; Iodine-125

Lay Summary (simple Prompt)

This study investigates a new method for predicting the movement of radioactive seeds used in brachytherapy, a common treatment for prostate cancer. The researchers analyzed data from 320 patients who received Iodine-125 seeds to target cancer cells. They found that seed density, which is the number of seeds implanted relative to the size of the prostate, is an important factor in predicting whether the seeds might migrate to other parts of the body. The study showed that higher seed density increases the likelihood of seed migration. Understanding this relationship can help doctors decide when to use additional measures, such as using different types of seeds, to prevent migration. These findings could lead to more precise and effective brachytherapy treatments for prostate cancer, reducing risks and improving patient outcomes.

Evaluation of the Lay Summary

**Assessment Category**	**Score**	**Assessment Category**	**Score**	**Assessment Category**	**Score**
Comprehension Score	5	Gunning Fog Score	16.0	Words	129
Accuracy Score	0	SMOG Index	11.8	Complex words	25
Reading Age (years)	19.5	Coleman Liau Index	15.9	Percent of complex words	19.4
Flesch Kincaid Reading Ease	39.4	Automated Readability Index	14.7	Average words per sentence	21.5
Flesch Kincaid Grade Level	13.1	Sentences	6	Average syllables per word	1.7

Lay Summary (extended Prompt)

This study looked at how to predict seed migration in prostate cancer patients treated with brachytherapy, a type of radiation therapy using Iodine-125 seeds. Seed migration occurs when the radioactive seeds move away from the target area in the body. The researchers examined the number of seeds and their density in the prostate to see if these factors could help predict migration. They found that a higher seed density (more seeds per volume of prostate) was linked to a greater chance of seeds moving. The study suggests that if the seed density is high, it may be helpful to use a different type of seed placement to prevent migration. This finding can help improve the safety and effectiveness of brachytherapy for prostate cancer patients by ensuring the seeds stay where they are most needed.

Evaluation of the Lay Summary

**Assessment Category**	**Score**	**Assessment Category**	**Score**	**Assessment Category**	**Score**
Comprehension Score	5	Gunning Fog Score	14.6	Words	135
Accuracy Score	0	SMOG Index	10.6	Complex words	20
Reading Age (years)	18.5	Coleman Liau Index	12.7	Percent of complex words	14.8
Flesch Kincaid Reading Ease	49.3	Automated Readability Index	12.6	Average words per sentence	22.5
Flesch Kincaid Grade Level	12.0	Sentences	6	Average syllables per word	1.6

Manuscript 37

Franco A, Pellegrino AA, De Nunzio C, Salkowski M, Jackson JC, Zukowski LB, Checcucci E, Vourganti S, Chow AK, Porpiglia F, Kaouk J, Crivellaro S, Autorino R. Single-Port Robot-Assisted Radical Prostatectomy: Where Do We Stand? Curr Oncol. 2023 Apr 20;30(4):4301-4310. doi: 10.3390/curroncol30040328. PMID: 37185441; PMCID: PMC10136812.

Abstract

In 2018, the da Vinci Single Port (SP) robotic system was approved by the US Food and Drug Administration for urologic procedures. Available studies for the application of SP to prostate cancer surgery are limited. The aim of our study is to summarize the current evidence on the techniques and outcomes of SP robot-assisted radical prostatectomy (SP-RARLP) procedures. A narrative review of the literature was performed in January 2023. Preliminary results suggest that SP-RALP is safe and feasible, and it can offer comparable outcomes to the standard multiport RALP. Extraperitoneal and transvesical SP-RALP appear to be the two most promising approaches, as they offer decreased invasiveness, potentially shorter length of stay, and better pain control. Long-term, high-quality data are missing and further validation with prospective studies across different sites is required.

Keywords:

single port; radical prostatectomy; robotic surgery; extraperitoneal

Lay Summary (simple Prompt)

This study reviews the current use of single-port robotic systems for performing radical prostatectomy, a surgery to remove the prostate gland in patients with prostate cancer. The da Vinci Single Port (SP) robotic system, approved in 2018, allows surgeons to perform the surgery through a single small incision. Early findings suggest that single-port robotic prostatectomy is safe and effective, offering similar outcomes to the traditional multiport approach. Two techniques, extraperitoneal and transvesical, seem particularly promising because they are less invasive and may lead to shorter hospital stays and better pain control. However, more high-quality, long-term studies are needed to confirm these benefits. This research could help guide future surgical practices, potentially leading to less invasive options and faster recovery times for prostate cancer patients.

Evaluation of the Lay Summary

**Assessment Category**	**Score**	**Assessment Category**	**Score**	**Assessment Category**	**Score**
Comprehension Score	5	Gunning Fog Score	17.6	Words	128
Accuracy Score	1	SMOG Index	12.7	Complex words	29
Reading Age (years)	21.5	Coleman Liau Index	17.8	Percent of complex words	22.7
Flesch Kincaid Reading Ease	28.5	Automated Readability Index	16.1	Average words per sentence	21.3
Flesch Kincaid Grade Level	14.6	Sentences	6	Average syllables per word	1.9

Lay Summary (extended Prompt)

This study explores the use of a new type of robotic surgery for prostate cancer called Single-Port Robot-Assisted Radical Prostatectomy (SP-RARLP). Unlike traditional methods that use multiple small cuts, this technique uses just one small incision, which could mean less pain and a quicker recovery for patients. The study reviews existing research to see how well this new method works. Early findings suggest that SP-RARLP is safe and effective, with results similar to the standard multiport approach. Two techniques, extraperitoneal and transvesical, seem particularly promising. However, more research is needed to confirm these findings and to see how well this surgery works in different hospitals and patient groups over the long term. This study could help improve treatment options for prostate cancer, making surgery less invasive and more comfortable for patients.

Evaluation of the Lay Summary

**Assessment Category**	**Score**	**Assessment Category**	**Score**	**Assessment Category**	**Score**
Comprehension Score	5	Gunning Fog Score	14.5	Words	135
Accuracy Score	0	SMOG Index	11.2	Complex words	26
Reading Age (years)	18.5	Coleman Liau Index	15.9	Percent of complex words	19.3
Flesch Kincaid Reading Ease	41.9	Automated Readability Index	13.6	Average words per sentence	19.3
Flesch Kincaid Grade Level	12.2	Sentences	7	Average syllables per word	1.7

Manuscript 38

Bianchi LMG, Irmici G, Cè M, D'Ascoli E, Della Pepa G, Di Vita F, Casati O, Soresina M, Menozzi A, Khenkina N, Cellina M. Diagnosis and Treatment of Post-Prostatectomy Lymphedema: What's New? Curr Oncol. 2023 Apr 25;30(5):4512-4526. doi: 10.3390/curroncol30050341. PMID: 37232799; PMCID: PMC10217297.

Abstract

Lymphedema is a chronic progressive disorder that significantly compromises patients' quality of life. In Western countries, it often results from cancer treatment, as in the case of post-radical prostatectomy lymphedema, where it can affect up to 20% of patients, with a significant disease burden. Traditionally, diagnosis, assessment of severity, and management of disease have relied on clinical assessment. In this landscape, physical and conservative treatments, including bandages and lymphatic drainage have shown limited results. Recent advances in imaging technology are revolutionizing the approach to this disorder: magnetic resonance imaging has shown satisfactory results in differential diagnosis, quantitative classification of severity, and most appropriate treatment planning. Further innovations in microsurgical techniques, based on the use of indocyanine green to map lymphatic vessels during surgery, have improved the efficacy of secondary LE treatment and led to the development of new surgical approaches. Physiologic surgical interventions, including lymphovenous anastomosis (LVA) and vascularized lymph node transplant (VLNT), are going to face widespread diffusion. A combined approach to microsurgical treatment provides the best results: LVA is effective in promoting lymphatic drainage, bridging VLNT delayed lymphangiogenic and immunological effects in the lymphatic impairment site. Simultaneous VLNT and LVA are safe and effective for patients with both early and advanced stages of post-prostatectomy LE. A new perspective is now represented by the combination of microsurgical treatments with the positioning of nano fibrillar collagen scaffolds (BioBridgeTM) to favor restoring the lymphatic function, allowing for improved and sustained volume reduction. In this narrative review, we proposed an overview of new strategies for diagnosing and treating post-prostatectomy lymphedema to get the most appropriate and successful patient treatment with an overview of the main artificial intelligence applications in the prevention, diagnosis, and management of lymphedema.

Keywords:

lymphedema; prostatectomy; lymphedema new treatments; robotic surgery; deep-learning segmentation systems

Lay Summary (simple Prompt)

This study reviews new advancements in diagnosing and treating lymphedema, a chronic swelling condition that can occur after prostate surgery. Traditional methods, like physical therapy and drainage, have had limited success. However, new imaging technologies, such as magnetic resonance imaging (MRI), are improving the ability to diagnose and plan treatments. Innovations in surgical techniques, including procedures that connect lymphatic vessels to veins and transplant healthy lymph nodes, have shown promising results in reducing swelling and improving patient outcomes. Combining these surgical approaches with new materials, like nano fibrillar collagen scaffolds, may further enhance the restoration of lymphatic function. The study also discusses the potential of artificial intelligence to improve the prevention, diagnosis, and management of lymphedema. These advances could lead to more effective and personalized treatments, improving the quality of life for patients who develop lymphedema after prostate surgery.

Evaluation of the Lay Summary

**Assessment Category**	**Score**	**Assessment Category**	**Score**	**Assessment Category**	**Score**
Comprehension Score	5	Gunning Fog Score	22.0	Words	139
Accuracy Score	1	SMOG Index	15.2	Complex words	49
Reading Age (years)	23.0	Coleman Liau Index	21.0	Percent of complex words	35.3
Flesch Kincaid Reading Ease	12.6	Automated Readability Index	17.9	Average words per sentence	19.9
Flesch Kincaid Grade Level	16.4	Sentences	7	Average syllables per word	2.1

Lay Summary (extended Prompt)

This study looks at new ways to diagnose and treat lymphedema, a common and serious side effect after prostate surgery. Lymphedema, which causes swelling due to a buildup of lymph fluid, can greatly affect a patient's quality of life. Traditional treatments like bandages and massages have limited success. However, recent advances in imaging, such as MRI, help better diagnose and plan treatment. New surgical techniques, like connecting lymph vessels to veins or transplanting lymph nodes, show promise in treating both early and advanced cases. Combining these surgeries with new materials, like collagen scaffolds, can improve lymphatic function and reduce swelling. This study also discusses the role of artificial intelligence in improving diagnosis and management of lymphedema, offering new hope for more effective treatments.

Evaluation of the Lay Summary

**Assessment Category**	**Score**	**Assessment Category**	**Score**	**Assessment Category**	**Score**
Comprehension Score	5	Gunning Fog Score	16.5	Words	123
Accuracy Score	0	SMOG Index	11.8	Complex words	29
Reading Age (years)	19.5	Coleman Liau Index	17.4	Percent of complex words	23.6
Flesch Kincaid Reading Ease	39.7	Automated Readability Index	13.9	Average words per sentence	17.6
Flesch Kincaid Grade Level	12.1	Sentences	7	Average syllables per word	1.8

Manuscript 39

Lanka SM, Zorko NA, Antonarakis ES, Barata PC. Metastatic Castration-Resistant Prostate Cancer, Immune Checkpoint Inhibitors, and Beyond. Curr Oncol. 2023 Apr 19;30(4):4246-4256. doi: 10.3390/curroncol30040323. PMID: 37185436; PMCID: PMC10137248.

Abstract

The therapeutic landscape of several genitourinary malignancies has been revolutionized by the development of immune checkpoint inhibitors (ICIs); however, the utility of immunotherapies in prostate cancer has been limited, partly due to the immunologically "cold" tumor terrain of prostate cancer. As of today, pembrolizumab is the only immune checkpoint inhibitor approved for the treatment of metastatic castration resistant prostate cancer (mCRPC) in a select group of patients with high microsatellite instability (MSI-H), deficient mismatch repair (dMMR), or high tumor mutational burden (TMB). Looking ahead, several combinatorial approaches with ICIs involving radioligands, radiotherapy, PARP inhibitors, interleukin inhibitors, and cancer vaccines are exploring a potential synergistic effect. Furthermore, B7-H3 is an alternative checkpoint that may hold promise in adding to the treatment landscape of mCRPC. This review aims to summarize previous monotherapy and combination therapy trials of ICIs as well as novel immunotherapy combination therapeutic strategies and treatment targets in mCRPC.

Keywords:

immune checkpoint inhibitors; metastatic castration-resistant prostate cancer; immunotherapy combinations

Lay Summary (simple Prompt)

This study reviews the current use and future potential of immune checkpoint inhibitors (ICIs) in treating metastatic castration-resistant prostate cancer (mCRPC), a form of advanced prostate cancer. ICIs have been successful in treating other cancers, but their effectiveness in prostate cancer has been limited because prostate tumors are often "cold," meaning they do not provoke a strong immune response. Currently, pembrolizumab is the only approved ICI for a small group of mCRPC patients with specific genetic characteristics. The study explores new strategies to enhance the effectiveness of ICIs by combining them with other treatments, such as radiotherapy, PARP inhibitors, and cancer vaccines. It also discusses promising new targets, like B7-H3, for future therapies. These findings highlight the potential of new combination treatments to improve outcomes for patients with advanced prostate cancer, potentially expanding the use of immunotherapy in this challenging condition.

Evaluation of the Lay Summary

Assessment Category	Score	Assessment Category	Score	Assessment Category	Score
Comprehension Score	5	Gunning Fog Score	17.7	Words	144
Accuracy Score	1	SMOG Index	12.7	Complex words	29
Reading Age (years)	21.5	Coleman Liau Index	17.9	Percent of complex words	20.1
Flesch Kincaid Reading Ease	25.0	Automated Readability Index	17.6	Average words per sentence	24.0
Flesch Kincaid Grade Level	15.7	Sentences	6	Average syllables per word	1.9

Lay Summary (extended Prompt)

This study explores new treatment options for men with advanced prostate cancer that has stopped responding to standard therapies. While immune checkpoint inhibitors (a type of immunotherapy) have been successful in treating other cancers, their use in prostate cancer has been limited. Currently, pembrolizumab is the only approved immunotherapy for certain prostate cancer patients with specific genetic features. Researchers are now looking into combining these immune therapies with other treatments, like radiotherapy, PARP inhibitors, and cancer vaccines, to improve their effectiveness. The study also highlights a new target, B7-H3, which may offer another treatment option in the future. Understanding these emerging strategies could help improve outcomes for men with this challenging type of prostate cancer.

Evaluation of the Lay Summary

**Assessment Category**	**Score**	**Assessment Category**	**Score**	**Assessment Category**	**Score**
Comprehension Score	5	Gunning Fog Score	15.3	Words	116
Accuracy Score	1	SMOG Index	11.1	Complex words	22
Reading Age (years)	20.5	Coleman Liau Index	19.0	Percent of complex words	19.0
Flesch Kincaid Reading Ease	29.7	Automated Readability Index	16.1	Average words per sentence	19.3
Flesch Kincaid Grade Level	13.9	Sentences	6	Average syllables per word	1.9

Manuscript 40

Mittal A, Sridhar SS, Ong M, Jiang DM. Triplet Therapy in Metastatic Castrate Sensitive Prostate Cancer (mCSPC)-A Potential New Standard of Care. Curr Oncol. 2023 Apr 20;30(4):4365-4378. doi: 10.3390/curroncol30040332. PMID: 37185445; PMCID: PMC10136811.

Abstract

The treatment paradigm for metastatic castrate-sensitive prostate cancer (mCSPC) has evolved rapidly in the past decade with the approval of several life-prolonging therapies including docetaxel chemotherapy and multiple androgen receptor pathway inhibitors (ARPI) in combination with androgen deprivation therapy (ADT). Recently reported phase-three trials have demonstrated a survival benefit of upfront triplet therapy with ADT, docetaxel plus either abiraterone acetate or darolutamide when compared to ADT plus docetaxel alone. However, multiple questions including the incremental benefit of docetaxel to a combination of ADT and ARPI, the timing of ARPI, optimal patient selection for triplet therapy and clinical and genomic biomarkers still remain to be answered. Moreover, real-world data suggest suboptimal treatment intensification with many patients treated with ADT alone highlighting challenges in implementation. In this article, we review the phase-three data associated with triplet therapy in mCSPC. We also discuss the knowledge gaps that exist despite the completion of these studies and how ongoing studies are likely to change the paradigm in the near future. Finally, we provide a simple algorithm based on current data that clinicians can use in daily practice to select patients for appropriate treatment strategies.

Keywords:

metastatic castrate sensitive prostate cancer; triplet therapy; androgen receptor pathway inhibitors; docetaxel; abiraterone; darolutamide

Lay Summary (simple Prompt)

This study reviews the use of a new "triplet therapy" approach for treating metastatic castrate-sensitive prostate cancer (mCSPC), which involves combining androgen deprivation therapy (ADT) with the chemotherapy drug docetaxel and either abiraterone acetate or darolutamide. Recent clinical trials have shown that this combination improves survival more than the standard treatment of ADT with docetaxel alone. However, questions remain about the best timing for adding these drugs, the specific benefits of using docetaxel in combination with other therapies, and which patients are most likely to benefit from this intensive treatment. The study highlights the need for further research to address these gaps and suggests a practical guide for doctors to select appropriate treatment strategies. The findings could help establish a new standard of care, improving outcomes for patients with advanced prostate cancer.

Evaluation of the Lay Summary

**Assessment Category**	**Score**	**Assessment Category**	**Score**	**Assessment Category**	**Score**
Comprehension Score	5	Gunning Fog Score	19.7	Words	133
Accuracy Score	1	SMOG Index	14.1	Complex words	30
Reading Age (years)	23.5	Coleman Liau Index	18.3	Percent of complex words	22.6
Flesch Kincaid Reading Ease	23.4	Automated Readability Index	19.2	Average words per sentence	26.6
Flesch Kincaid Grade Level	16.6	Sentences	5	Average syllables per word	1.9

Lay Summary (extended Prompt)

This study explores a new potential standard treatment for men with metastatic castrate-sensitive prostate cancer (mCSPC). Traditionally, these patients have been treated with androgen deprivation therapy (ADT) alone or in combination with other drugs like chemotherapy (docetaxel) or androgen receptor pathway inhibitors (ARPI). Recent clinical trials have shown that using a combination of three treatments—ADT, docetaxel, and either abiraterone acetate or darolutamide—can improve survival rates more than just two treatments. However, there are still questions about the best way to use these drugs together and which patients would benefit the most. The study also highlights the gap between clinical trial results and real-world practice, where many patients are still treated with ADT alone. This research provides a clearer understanding for doctors on how to choose the best treatment plan for their patients.

Evaluation of the Lay Summary

**Assessment Category**	**Score**	**Assessment Category**	**Score**	**Assessment Category**	**Score**
Comprehension Score	5	Gunning Fog Score	16.7	Words	134
Accuracy Score	0	SMOG Index	12.0	Complex words	26
Reading Age (years)	21.5	Coleman Liau Index	17.8	Percent of complex words	19.4
Flesch Kincaid Reading Ease	31.4	Automated Readability Index	16.6	Average words per sentence	22.3
Flesch Kincaid Grade Level	14.4	Sentences	6	Average syllables per word	1.8

Manuscript 41

Riccio G, Berenguer CV, Perestrelo R, Pereira F, Berenguer P, Ornelas CP, Sousa AC, Vital JA, Pinto MDC, Pereira JAM, Greco V, Câmara JS. Differences in the Volatilomic Urinary Biosignature of Prostate Cancer Patients as a Feasibility Study for the Detection of Potential Biomarkers. Curr Oncol. 2023 May 10;30(5):4904-4921. doi: 10.3390/curroncol30050370. PMID: 37232828; PMCID: PMC10217291.

Abstract

Prostate cancer (PCa) continues to be the second most common malignant tumour and the main cause of oncological death in men. Investigating endogenous volatile organic metabolites (VOMs) produced by various metabolic pathways is emerging as a novel, effective, and non-invasive source of information to establish the volatilomic biosignature of PCa. In this study, headspace solid-phase microextraction combined with gas chromatography–mass spectrometry (HS-SPME/GC-MS) was used to establish the urine volatilomic profile of PCa and identify VOMs that can discriminate between the two investigated groups. This non-invasive approach was applied to oncological patients (PCa group, n = 26) and cancer-free individuals (control group, n = 30), retrieving a total of 147 VOMs from various chemical families. This included terpenes, norisoprenoid, sesquiterpenes, phenolic, sulphur and furanic compounds, ketones, alcohols, esters, aldehydes, carboxylic acid, benzene and naphthalene derivatives, hydrocarbons, and heterocyclic hydrocarbons. The data matrix was subjected to multivariate analysis, namely partial least-squares discriminant analysis (PLS-DA). Accordingly, this analysis showed that the group under study presented different volatomic profiles and suggested potential PCa biomarkers. Nevertheless, a larger cohort of samples is required to boost the predictability and accuracy of the statistical models developed.

Keywords:

prostate cancer; volatilomics; urine; biomarkers

Lay Summary (simple Prompt)

This study explores a new, non-invasive method for detecting prostate cancer by analyzing the volatile organic compounds (VOMs) found in urine. Researchers used advanced techniques to identify 147 different chemical compounds in the urine of prostate cancer patients and compared them with those of cancer-free individuals. The results showed distinct patterns, or "biosignatures," in the VOMs of prostate cancer patients, suggesting these compounds could serve as potential biomarkers for the disease. Identifying such biomarkers could lead to easier, non-invasive testing methods for early detection of prostate cancer. However, more research with larger sample sizes is needed to confirm these findings and improve the accuracy of this approach. This study could pave the way for new diagnostic tools that enhance early detection and treatment strategies for prostate cancer.

Evaluation of the Lay Summary

**Assessment Category**	**Score**	**Assessment Category**	**Score**	**Assessment Category**	**Score**
Comprehension Score	5	Gunning Fog Score	16.1	Words	131
Accuracy Score	0	SMOG Index	11.8	Complex words	25
Reading Age (years)	20.5	Coleman Liau Index	17.0	Percent of complex words	19.1
Flesch Kincaid Reading Ease	34.8	Automated Readability Index	15.7	Average words per sentence	21.8
Flesch Kincaid Grade Level	13.8	Sentences	6	Average syllables per word	1.8

Lay Summary (extended Prompt)

This study explores a new, non-invasive method to detect prostate cancer by analyzing the chemicals found in urine. Researchers used advanced techniques to study the urine of men with and without prostate cancer. They identified 147 different types of chemicals that may help distinguish between the two groups. These chemicals could serve as potential biomarkers, which are signs in the body that can indicate the presence of cancer. The results show that men with prostate cancer have different chemical profiles in their urine compared to men without the disease. While these findings are promising, more research with a larger group of participants is needed to confirm these results and improve the accuracy of this method. This approach could one day help doctors diagnose prostate cancer more easily and without invasive procedures.

Evaluation of the Lay Summary

**Assessment Category**	**Score**	**Assessment Category**	**Score**	**Assessment Category**	**Score**
Comprehension Score	5	Gunning Fog Score	14.2	Words	132
Accuracy Score	0	SMOG Index	10.3	Complex words	22
Reading Age (years)	18.5	Coleman Liau Index	15.2	Percent of complex words	16.7
Flesch Kincaid Reading Ease	49.3	Automated Readability Index	12.8	Average words per sentence	18.9
Flesch Kincaid Grade Level	11.1	Sentences	7	Average syllables per word	1.6

Manuscript 42

Oderda M, Dematteis A, Calleris G, Conti A, D'Agate D, Falcone M, Marquis A, Montefusco G, Marra G, Gontero P. Predictors of Prostate Cancer at Fusion Biopsy: The Role of Positive Family History, Hypertension, Diabetes, and Body Mass Index. Curr Oncol. 2023 May 12;30(5):4957-4965. doi: 10.3390/curroncol30050374. PMID: 37232832; PMCID: PMC10217714.

Abstract

Background: PSA density and an elevated PI-RADS score are among the strongest predictors of prostate cancer (PCa) in a fusion biopsy. Positive family history, hypertension, diabetes, and obesity have also been associated with the risk of developing PCa. We aim to identify predictors of the prostate cancer detection rate (CDR) in a series of patients undergoing a fusion biopsy. Methods: We retrospectively evaluated 736 consecutive patients who underwent an elastic fusion biopsy from 2020 to 2022. Targeted biopsies (2–4 cores per MRI target) were followed by systematic mapping (10–12 cores). Clinically significant PCa (csPCa) was defined as ISUP score ≥ 2. Uni- and multi-variable logistic regression analyses were performed to identify predictors of CDR among age, body mass index (BMI), hypertension, diabetes, positive family history, PSA, a positive digital rectal examination (DRE), PSA density ≥ 0.15, previous negative biopsy status, PI-RADS score, and size of MRI lesion. Results: The median patients' age was 71 years, and median PSA was 6.6 ng/mL. A total of 20% of patients had a positive digital rectal examination. Suspicious lesions in mpMRI were scored as 3, 4, and 5 in 14.9%, 55.0%, and 17.5% of cases, respectively. The CDR was 63.2% for all cancers and 58.7% for csPCa. Only age (OR 1.04, $p < 0.001$), a positive DRE (OR 1.75, $p = 0.04$), PSA density (OR 2.68, $p < 0.001$), and elevated PI-RADS score (OR 4.02, $p = 0.003$) were significant predictors of the CDR in the multivariable analysis for overall PCa. The same associations were found for csPCa. The size of an MRI lesion was associated with the CDR only in uni-variable analysis (OR 1.07, $p < 0.001$). BMI, hypertension, diabetes, and a positive family history were not predictors of PCa. Conclusions: In a series of patients selected for a fusion biopsy, positive family history, hypertension, diabetes, or BMI are not predictors of PCa detection. PSA-density and PI-RADS score are confirmed to be strong predictors of the CDR.

Keywords:

fusion biopsy; prostate cancer; risk factors; family history; hypertension; diabetes; body mass index

Lay Summary (simple Prompt)

This study aimed to identify factors that predict the likelihood of detecting prostate cancer during a fusion biopsy, a procedure that combines MRI imaging with targeted tissue sampling. The researchers analyzed data from 736 patients and found that certain clinical factors, such as age, positive digital rectal examination (DRE), PSA density, and high PI-RADS score, were strong predictors of prostate cancer detection. In contrast, factors like a positive family history, hypertension, diabetes, and body mass index (BMI) were not associated with an increased risk of detecting prostate cancer. These findings suggest that while some common health conditions and family history do not influence cancer detection rates, specific clinical and imaging factors remain crucial for identifying prostate cancer. This knowledge can help doctors better assess which patients are most likely to benefit from a biopsy, leading to more targeted and effective screening strategies.

Evaluation of the Lay Summary

**Assessment Category**	**Score**	**Assessment Category**	**Score**	**Assessment Category**	**Score**
Comprehension Score	5	Gunning Fog Score	21.5	Words	143
Accuracy Score	1	SMOG Index	15.4	Complex words	36
Reading Age (years)	23.5	Coleman Liau Index	17.3	Percent of complex words	25.2
Flesch Kincaid Reading Ease	21.6	Automated Readability Index	19.4	Average words per sentence	28.6
Flesch Kincaid Grade Level	17.3	Sentences	5	Average syllables per word	1.9

Lay Summary (extended Prompt)

This study looked at factors that predict the chance of finding prostate cancer in men undergoing a special type of biopsy called a fusion biopsy. The researchers analyzed data from 736 patients who had this procedure. They wanted to see if factors like family history of cancer, high blood pressure, diabetes, and body weight affected cancer detection rates. The study found that a higher PSA density, an elevated PI-RADS score from MRI scans, older age, and an abnormal digital rectal exam were the strongest predictors of finding prostate cancer. However, having a family history of cancer, high blood pressure, diabetes, or a higher body mass index did not predict the presence of prostate cancer. These findings help doctors better understand which factors are most useful in predicting prostate cancer, which can improve how they select patients for biopsy.

Evaluation of the Lay Summary

**Assessment Category**	**Score**	**Assessment Category**	**Score**	**Assessment Category**	**Score**
Comprehension Score	5	Gunning Fog Score	15.9	Words	139
Accuracy Score	0	SMOG Index	11.3	Complex words	23
Reading Age (years)	19.5	Coleman Liau Index	14.1	Percent of complex words	16.6
Flesch Kincaid Reading Ease	43.3	Automated Readability Index	14.0	Average words per sentence	23.2
Flesch Kincaid Grade Level	13.0	Sentences	6	Average syllables per word	1.7

Manuscript 43

Fujii K, Nakano M, Kawakami S, Tanaka Y, Kainuma T, Tsumura H, Tabata KI, Satoh T, Iwamura M, Ishiyama H. Dosimetric Predictors of Toxicity after Prostate Stereotactic Body Radiotherapy: A Single-Institutional Experience of 145 Patients. Curr Oncol. 2023 May 16;30(5):5062-5071. doi: 10.3390/curroncol30050383. PMID: 37232841; PMCID: PMC10217554.

Abstract

The indications for stereotactic body radiotherapy (SBRT) for prostate cancer have increased. However, the relationships between adverse events and risk factors remain unclear. This study aimed to clarify associations between adverse events and dose index for prostate SBRT. Participants comprised 145 patients irradiated with 32–36 Gy in 4 fractions. Radiotherapy-related risk factors such as dose-volume histogram parameters and patient-related risk factors such as T stage and Gleason score were evaluated in a competing risk analysis. Median follow-up duration was 42.9 months. A total of 9.7% had acute Grade ≥ 2 GU toxicities and 4.8% had acute Grade ≥ 2 GI toxicities. A total of 11.1% had late Grade ≥ 2 GU toxicities and 7.6% had late Grade ≥ 2 GI toxicities. Two (1.4%) patients suffered from late Grade 3 GU toxicities. Similarly, two (1.4%) patients suffered from late Grade 3 GI toxicities. Acute GU and GI events correlated with prostate volume and dose to the hottest 10 cc volume (D10cc)/volumes receiving a minimum of 30 Gy (V30 Gy) of rectum, respectively. Late GI toxicity, frequency, and rectal hemorrhage correlated with rectal D0.1 cc/D1 cc, maximum dose to the bladder, and rectal D0.1 cc, respectively. Toxicities after prostate SBRT using 32–36 Gy/4 fractions were acceptable. Our analysis showed that acute toxicities correlated with volume receiving a medium dose level, and late toxicities correlated with highest point dose of organs at risk.

Keywords:

prostate cancer; stereotactic body radiotherapy; genitourinary toxicity; gastrointestinal toxicity

Lay Summary (simple Prompt)

This study examines the factors that predict side effects in prostate cancer patients treated with stereotactic body radiotherapy (SBRT), a highly focused form of radiation therapy. The research involved 145 patients and aimed to understand how different radiation doses and patient characteristics are related to adverse effects, such as urinary and gastrointestinal problems. The findings showed that the likelihood of acute side effects (occurring shortly after treatment) was linked to the amount of radiation given to specific areas of the prostate and surrounding tissues. Similarly, late side effects (developing months or years later) were associated with the highest doses of radiation to nearby organs like the bladder and rectum. Understanding these relationships can help doctors better plan SBRT treatments to minimize side effects while effectively treating prostate cancer, improving the overall safety and quality of life for patients.

Evaluation of the Lay Summary

**Assessment Category**	**Score**	**Assessment Category**	**Score**	**Assessment Category**	**Score**
Comprehension Score	5	Gunning Fog Score	19.4	Words	138
Accuracy Score	1	SMOG Index	13.9	Complex words	29
Reading Age (years)	23.5	Coleman Liau Index	18.2	Percent of complex words	21.0
Flesch Kincaid Reading Ease	18.8	Automated Readability Index	19.5	Average words per sentence	27.6
Flesch Kincaid Grade Level	17.5	Sentences	5	Average syllables per word	1.9

Lay Summary (extended Prompt)

This study looked at the side effects experienced by prostate cancer patients who received a type of targeted radiation treatment called stereotactic body radiotherapy (SBRT). The aim was to find out what factors, like the amount of radiation and specific patient characteristics, might predict higher chances of developing side effects such as urinary and bowel problems. Out of 145 patients, some experienced short-term (acute) side effects, while others had long-term (late) side effects. The study found that the size of the prostate and the amount of radiation to certain parts of the body were linked to the side effects patients experienced. Understanding these relationships helps doctors better predict and manage side effects, improving the overall safety and effectiveness of this type of prostate cancer treatment.

Evaluation of the Lay Summary

**Assessment Category**	**Score**	**Assessment Category**	**Score**	**Assessment Category**	**Score**
Comprehension Score	5	Gunning Fog Score	15.2	Words	127
Accuracy Score	0	SMOG Index	10.4	Complex words	16
Reading Age (years)	19.5	Coleman Liau Index	15.8	Percent of complex words	12.6
Flesch Kincaid Reading Ease	39.8	Automated Readability Index	16.5	Average words per sentence	25.4
Flesch Kincaid Grade Level	14.0	Sentences	5	Average syllables per word	1.7

Manuscript 44

Maselli FM, Giuliani F, Laface C, Perrone M, Melaccio A, De Santis P, Santoro AN, Guarini C, Iaia ML, Fedele P. Immunotherapy in Prostate Cancer: State of Art and New Therapeutic Perspectives. Curr Oncol. 2023 Jun 13;30(6):5769-5794. doi: 10.3390/curroncol30060432. PMID: 37366915; PMCID: PMC10297468.

Abstract

Prostate cancer (PC) is the most common type of tumor in men. In the early stage of the disease, it is sensitive to androgen deprivation therapy. In patients with metastatic castration-sensitive prostate cancer (mHSPC), chemotherapy and second-generation androgen receptor therapy have led to increased survival. However, despite advances in the management of mHSPC, castration resistance is unavoidable and many patients develop metastatic castration-resistant disease (mCRPC). In the past few decades, immunotherapy has dramatically changed the oncology landscape and has increased the survival rate of many types of cancer. However, immunotherapy in prostate cancer has not yet given the revolutionary results it has in other types of tumors. Research into new treatments is very important for patients with mCRPC because of its poor prognosis. In this review, we focus on the reasons for the apparent intrinsic resistance of prostate cancer to immunotherapy, the possibilities for overcoming this resistance, and the clinical evidence and new therapeutic perspectives regarding immunotherapy in prostate cancer with a look toward the future.

Keywords:

prostate cancer; immunotherapy

Lay Summary (simple Prompt)

This study reviews the current state and future potential of immunotherapy for treating prostate cancer, the most common cancer in men. While early-stage prostate cancer responds well to hormone therapy, advanced stages often develop resistance, leading to a challenging condition known as metastatic castration-resistant prostate cancer (mCRPC). Unlike in other cancers, immunotherapy has not yet shown dramatic results in treating prostate cancer. This review examines why prostate cancer seems resistant to immunotherapy and explores new strategies to overcome this resistance. The study highlights the importance of ongoing research to find more effective treatments for mCRPC, as current options have limited success. The findings could help guide future efforts to develop better immunotherapy approaches, potentially improving outcomes for patients with advanced prostate cancer.

Evaluation of the Lay Summary

Assessment Category	Score	Assessment Category	Score	Assessment Category	Score
Comprehension Score	5	Gunning Fog Score	16.7	Words	124
Accuracy Score	1	SMOG Index	12.0	Complex words	26
Reading Age (years)	21.5	Coleman Liau Index	19.8	Percent of complex words	21.0
Flesch Kincaid Reading Ease	26.9	Automated Readability Index	17.4	Average words per sentence	20.7
Flesch Kincaid Grade Level	14.6	Sentences	6	Average syllables per word	1.9

Lay Summary (extended Prompt)

Prostate cancer is the most common cancer in men and is often treatable in its early stages with hormone therapy. However, when the cancer spreads and becomes resistant to treatment, it is much harder to manage. While immunotherapy has been successful in treating other types of cancer, it has not yet shown the same success for prostate cancer. This study looks at why prostate cancer is resistant to immunotherapy and explores new approaches that might overcome this challenge. Finding effective immunotherapy options for prostate cancer could significantly improve the outcomes for patients with advanced stages of the disease. This research is important because it aims to provide new treatment possibilities for those who have limited options currently.

Evaluation of the Lay Summary

**Assessment Category**	**Score**	**Assessment Category**	**Score**	**Assessment Category**	**Score**
Comprehension Score	5	Gunning Fog Score	13.3	Words	117
Accuracy Score	1	SMOG Index	9.5	Complex words	16
Reading Age (years)	18.5	Coleman Liau Index	16.1	Percent of complex words	13.7
Flesch Kincaid Reading Ease	44.6	Automated Readability Index	13.8	Average words per sentence	19.5
Flesch Kincaid Grade Level	11.9	Sentences	6	Average syllables per word	1.7

Manuscript 45

Venugopal B, Shahhat S, Beck J, Hanumanthappa N, Ong AD, Dubey A, Koul R, Bashir B, Chowdhury A, Sivananthan G, Kim JO. Factors Associated with Long-Term Prostate Cancer Survival after Palliative Radiotherapy to a Bone Metastasis and Contemporary Palliative Systemic Therapy: A Retrospective, Population-Based Study. Curr Oncol. 2023 Jun 9;30(6):5560-5573. doi: 10.3390/curroncol30060420. PMID: 37366903; PMCID: PMC10297007.

Abstract

Background: Radiation therapy (RT) is an established palliative treatment for bone metastases; however, little is known about post-radiation survival and factors which impact it. The aim of this study was to assess a population-based sample of metastatic prostate cancer patients receiving palliative radiation therapy to bone metastases and contemporary palliative systemic therapy and identify factors that impact long-term survival. Materials/methods: This retrospective, population-based, cohort study assessed all prostate cancer patients receiving palliative RT for bone metastases at a Canadian provincial Cancer program during a contemporary time period. Baseline patient, disease, and treatment characteristics were extracted from the provincial medical physics databases and the electronic medical record. Post-RT Survival intervals were defined as the time interval from the first fraction of palliative RT to death from any cause or date of the last known follow-up. The median survival of the cohort was used to dichotomize the cohort into short- and long-term survivors following RT. Univariable and multivariable hazard regression analyses were performed to identify variables associated with post-RT survival. Results: From 1 January 2018 until 31 December 2019, 545 palliative RT courses for bone metastases were delivered to n = 274 metastatic prostate cancer patients with a median age of 76 yrs (Interquartile range (IQR) 39–83) and a median follow-up of 10.6 months (range 0.2 to 47.9). The median survival of the cohort was 10.6 months (IQR 3.5–25 months). The ECOG performance status of the whole cohort was ≤2 in n = 200 (73%) and 3–4 in n = 67 (24.5%). The most commonly treated sites of bone metastasis were the pelvis and lower extremities n = 130 (47.4%), skull and spine n = 114 (41.6%), and chest and upper extremities n = 30 (10.9%). Most patients had CHAARTED high volume disease n = 239 (87.2%). On multivariable hazard regression analysis, an

ECOG performance status of 3–4 ($p = 0.02$), CHAARTED high volume disease burden ($p = 0.023$), and non-receipt of systemic therapy ($p = 0.006$) were significantly associated with worse post-RT survival. Conclusion: Amongst metastatic prostate cancer patients treated with palliative radiotherapy to bone metastases and modern palliative systemic therapies, ECOG performance status, CHAARTED metastatic disease burden, and type of first-line palliative systemic therapy were significantly associated with post-RT survival durations.

Keywords:
metastatic prostate cancer; bone metastases; palliative radiotherapy; survival intervals; prognostic factors

Lay Summary (simple Prompt)

This study investigates the factors that influence long-term survival in prostate cancer patients with bone metastases who receive palliative radiation therapy. By analyzing data from 274 patients in a Canadian cancer program, the researchers aimed to understand which characteristics affect survival after radiation treatment. They found that patients with a poorer overall health status, a high volume of metastatic disease, and those who did not receive additional systemic therapy had shorter survival times following radiation. The study suggests that assessing these factors can help doctors better predict outcomes and tailor treatment plans for prostate cancer patients with bone metastases. Understanding which patients are likely to benefit most from certain treatments could improve care and support for those living with advanced prostate cancer.

Evaluation of the Lay Summary

Assessment Category	Score	Assessment Category	Score	Assessment Category	Score
Comprehension Score	5	Gunning Fog Score	17.6	Words	123
Accuracy Score	1	SMOG Index	12.9	Complex words	25
Reading Age (years)	22.5	Coleman Liau Index	18.9	Percent of complex words	20.3
Flesch Kincaid Reading Ease	26.4	Automated Readability Index	18.6	Average words per sentence	24.6
Flesch Kincaid Grade Level	15.7	Sentences	5	Average syllables per word	1.9

Lay Summary (extended Prompt)

This study looked at factors that affect how long men with advanced prostate cancer survive after receiving radiation therapy to ease bone pain caused by cancer spread. Researchers reviewed the records of 274 patients who received this treatment in Canada between 2018 and 2019. They found that most men lived for about 11 months after their treatment. However, those who were in better overall health, had less extensive cancer spread, and received additional treatments lived longer. The study shows that the overall health of the patient, the amount of cancer spread, and whether they received more therapies are important in predicting survival after radiation. These findings can help doctors better understand who might benefit most from certain treatments and improve the care of patients with advanced prostate cancer.

Evaluation of the Lay Summary

**Assessment Category**	**Score**	**Assessment Category**	**Score**	**Assessment Category**	**Score**
Comprehension Score	4	Gunning Fog Score	13.5	Words	128
Accuracy Score	0	SMOG Index	9.8	Complex words	17
Reading Age (years)	18.5	Coleman Liau Index	15.2	Percent of complex words	13.3
Flesch Kincaid Reading Ease	52.3	Automated Readability Index	14.1	Average words per sentence	21.3
Flesch Kincaid Grade Level	11.3	Sentences	6	Average syllables per word	1.6

Manuscript 46

Ito M, Makita C, Mori T, Takano H, Kumano T, Matsuo M, Iinuma K, Kawase M, Nakane K, Nakano M, Koie T. Associations of Clinical and Dosimetric Parameters with Urinary Toxicities after Prostate Brachytherapy: A Long-Term Single-Institution Experience. Curr Oncol. 2023 Jun 9;30(6):5680-5689. doi: 10.3390/curroncol30060426. PMID: 37366909; PMCID: PMC10297351.

Abstract

To examine the association of clinical, treatment, and dose parameters with late urinary toxicity after low-dose-rate brachytherapy (LDR-BT) for prostate cancer, we retrospectively studied patients with prostate cancer who underwent LDR-BT from January 2007 through December 2016. Urinary toxicity was assessed using the International Prostate Symptom Score (IPSS) and Overactive Bladder (OAB) Symptom Score (OABSS). Severe and moderate lower urinary tract symptoms (LUTS) were defined as IPSS ≥ 20 and ≥ 8, respectively; OAB was defined as a nocturnal frequency of ≥ 2 and a total OABSS of ≥ 3. In total, 203 patients (median age: 66 years) were included, with a mean follow-up of 8.4 years after treatment. The IPSS and OABSS worsened after 3 months of treatment; these scores improved to pretreatment levels after 18–36 months in most patients. Patients with a higher baseline IPSS and OABSS had a higher frequency of moderate and severe LUTS and OAB at 24 and 60 months, respectively. LUTS and OAB at 24 and 60 months were not correlated with the dosimetric factors of LDR-BT. Although the rate of long-term urinary toxicities assessed using IPSS and OABSS was low, the baseline scores were related to long-term function. Refining patient selection may further reduce long-term urinary toxicity.

Keywords:

prostate cancer; brachytherapy; lower urinary tract symptoms; international prostate symptom score (IPSS); overactive bladder symptom score (OABSS)

Lay Summary (simple Prompt)

This study investigates the long-term effects of low-dose-rate brachytherapy, a type of radiation treatment, on urinary symptoms in prostate cancer patients. Researchers analyzed data from 203 patients over an average follow-up of 8.4 years to see how treatment impacted urinary health. They found that while urinary symptoms, such as frequent urination and overactive bladder, worsened shortly after treatment, most patients' symptoms returned to pre-treatment levels within 18-36 months. However, patients with higher initial urinary symptom scores were more likely to experience ongoing symptoms. The study suggests that assessing patients' baseline urinary health before treatment could help predict who may have long-term urinary issues, potentially improving patient selection and reducing side effects in the future. These findings can guide healthcare providers in managing and minimizing urinary problems for prostate cancer patients receiving brachytherapy.

Evaluation of the Lay Summary

**Assessment Category**	**Score**	**Assessment Category**	**Score**	**Assessment Category**	**Score**
Comprehension Score	5	Gunning Fog Score	16.6	Words	140
Accuracy Score	1	SMOG Index	12.0	Complex words	30
Reading Age (years)	20.5	Coleman Liau Index	18.0	Percent of complex words	21.4
Flesch Kincaid Reading Ease	27.0	Automated Readability Index	15.6	Average words per sentence	20.0
Flesch Kincaid Grade Level	14.5	Sentences	7	Average syllables per word	1.9

Lay Summary (extended Prompt)

This study looked at how prostate cancer treatment with low-dose-rate brachytherapy (a type of internal radiation) affects long-term urinary health. Researchers wanted to see if certain factors could predict who might have more severe urinary problems after treatment. They studied 203 patients and tracked their urinary symptoms for an average of 8.4 years. They found that urinary symptoms often got worse three months after treatment but mostly returned to normal within 18 to 36 months. Patients who already had urinary problems before treatment were more likely to have ongoing symptoms. The study did not find a link between the amount of radiation used and long-term urinary issues. The findings suggest that choosing patients more carefully before treatment might help reduce long-term urinary problems.

Evaluation of the Lay Summary

**Assessment Category**	**Score**	**Assessment Category**	**Score**	**Assessment Category**	**Score**
Comprehension Score	5	Gunning Fog Score	11.1	Words	129
Accuracy Score	0	SMOG Index	8.0	Complex words	15
Reading Age (years)	16.5	Coleman Liau Index	14.5	Percent of complex words	11.6
Flesch Kincaid Reading Ease	51.4	Automated Readability Index	10.9	Average words per sentence	16.1
Flesch Kincaid Grade Level	10.1	Sentences	8	Average syllables per word	1.6

Manuscript 47

García-Zoghby L, Lucas-Lucas C, Amo-Salas M, Soriano-Castrejón ÁM, García-Vicente AM. Head-to-Head Comparison of [^{18}F]F-choline and Imaging of Prostate-Specific Membrane Antigen, Using [^{18}F]DCFPyL PET/CT, in Patients with Biochemical Recurrence of Prostate Cancer. Curr Oncol. 2023 Jun 30;30(7):6271-6288. doi: 10.3390/curroncol30070464. PMID: 37504324; PMCID: PMC10378109.

Abstract

Purpose: To analyse diagnostic and therapeutic impact of molecular imaging TNM (miTNM) stage obtained with [18F]DCFPyL versus [18F]F-choline in head-to-head comparison in biochemical recurrence (BCR) of prostate cancer (PCa). Material and methods: Patients with BCR of PCa after radical treatment with previous [18F]F-choline-PET/CT (negative or oligometastatic disease) were recruited to [18F]DCFPyL-PET/CT. Patients were classified according to: grade group, European Association of Urology classification, PSA, PSA doubling time (PSAdt) and PSA velocity (PSAvel). The overall detection rate (DR) and miTNM stage according to PROMISE criteria were assessed for both radiotracers and also correlated (Kappa). The influence of PSA and kinetics on both PET/CT (DR and miTNM) and predictive value of unfavourable kinetics on miTNM were determined. Cut-off PSA, PSAdt and PSAvel values able to predict PET/CT results were determined. Change in miTNM and treatment derived from [18F]DCFPyL information compared with [18F]F-choline were also evaluated. Results: We studied 138 patients. [18F]DCFPyL showed a higher DR than [18F]F-choline (64.5% versus 33.3%) with a fair agreement. [18F]DCFPyL and [18F]F-choline detected T in 33.3% versus 19.6%, N in 27.5% versus 13.8%, and M in 30.4% versus 8.7%. Both tracers' DR showed significant associations with PSA and PSAvel. Significant association was only found between miTNM and PSA on [18F]F-choline-PET/CT ($p = 0.033$). For [18F]F-choline and [18F]DCFPyL-PET/CT, a PSAdt cut-off of 4.09 and 5.59 months, respectively, were able to predict M stage. [18F]DCFPyL changed therapeutic management in 40/138 patients. Conclusions: [18F]DCFPyL provides a higher DR and superior miTNM staging than [18F]F-choline in restaging BCR, especially with high PSA and unfavourable PSA kinetics, showing a fair agreement to [18F]F-choline.

Keywords:

[18F]DCFPyL; [18F]F-choline; miTNM; PSA level; PSA kinetics; therapeutic impact

<u>Lay Summary (simple Prompt)</u>

This study compares two imaging techniques, [18F]DCFPyL PET/CT and [18F]F-choline PET/CT, to see which is better at detecting prostate cancer recurrence after initial treatment. The researchers looked at how well each method identified cancer in different areas of the body and how these findings impacted treatment decisions. They found that [18F]DCFPyL PET/CT had a higher detection rate than [18F]F-choline PET/CT and provided more accurate staging of the cancer, especially in patients with higher levels of PSA, a marker of prostate cancer. Using [18F]DCFPyL also led to changes in treatment for many patients. The results suggest that [18F]DCFPyL PET/CT could be a more effective tool for managing prostate cancer recurrence, helping doctors make better-informed decisions about patient care.

<u>Evaluation of the Lay Summary</u>

**Assessment Category**	**Score**	**Assessment Category**	**Score**	**Assessment Category**	**Score**
Comprehension Score	5	Gunning Fog Score	15.6	Words	120
Accuracy Score	0	SMOG Index	11.0	Complex words	18
Reading Age (years)	19.5	Coleman Liau Index	15.6	Percent of complex words	15.0
Flesch Kincaid Reading Ease	44.3	Automated Readability Index	15.7	Average words per sentence	24.0
Flesch Kincaid Grade Level	13.0	Sentences	5	Average syllables per word	1.6

Lay Summary (extended Prompt)

This study compares two imaging methods, [18F]DCFPyL PET/CT and [18F]F-choline PET/CT, used to detect the return of prostate cancer after initial treatment. The researchers wanted to see which method was better at finding cancer that had come back. They looked at 138 patients with biochemical recurrence, which means their prostate cancer might have returned. The study found that [18F]DCFPyL was more effective at detecting cancer in the prostate, lymph nodes, and distant sites compared to [18F]F-choline. It also helped change treatment plans for many patients based on more accurate cancer staging. This finding suggests that [18F]DCFPyL could be a better tool for doctors to use when deciding how to treat patients whose prostate cancer has returned, particularly for those with higher PSA levels and faster PSA increases.

Evaluation of the Lay Summary

**Assessment Category**	**Score**	**Assessment Category**	**Score**	**Assessment Category**	**Score**
Comprehension Score	5	Gunning Fog Score	11.7	Words	129
Accuracy Score	0	SMOG Index	7.6	Complex words	10
Reading Age (years)	17.5	Coleman Liau Index	14.5	Percent of complex words	7.8
Flesch Kincaid Reading Ease	56.5	Automated Readability Index	13.5	Average words per sentence	21.5
Flesch Kincaid Grade Level	10.7	Sentences	6	Average syllables per word	1.5

Manuscript 48

Mardani A, Farahani MA, Khachian A, Vaismoradi M. Fear of Cancer Recurrence and Coping Strategies among Prostate Cancer Survivors: A Qualitative Study. Curr Oncol. 2023 Jul 16;30(7):6720-6733. doi: 10.3390/curroncol30070493. PMID: 37504353; PMCID: PMC10378434.

Abstract

Background: Fear of cancer recurrence (FCR), as a commonly reported problem among prostate cancer survivors, has not been fully understood. This study aimed to explore the experience of FCR and relevant coping strategies among Iranian prostate cancer survivors. Methods: Qualitative research was conducted on 13 men who completed treatments for prostate cancer in the last 24 months. The participants were selected through purposeful sampling, and in-depth semi-structured interviews were used for data collection. Conventional content analysis was used for data analysis. Results: Data analysis led to the emergence of three themes. “Living with insecurity” describes the participants’ experiences regarding what triggers FCR with two categories, including “fear of incomplete cure” and “fear of cancer return.” In addition, “struggling to cope” with two categories, including “psychological strategies” and “spiritual coping,” presents coping strategies used by the participants for reducing FCR. Furthermore, “trying to prevent cancer recurrence” with two categories, “seeking health” and “lifestyle modification,” indicates coping strategies used by the participants to prevent cancer recurrence. Conclusions: Healthcare providers need to consider the cultural characteristics of prostate cancer survivors when assessing their FCR, encourage them to disclose their concerns and fears, and provide tailored interventions in order to reduce FCR among them.

Keywords:

coping; continuous care; fear of cancer recurrence; prostate cancer survivors; qualitative research

Lay Summary (simple Prompt)

This study explores the fear of cancer recurrence (FCR) and coping strategies among prostate cancer survivors in Iran. Through interviews with 13 men who had completed treatment in the past two years, researchers aimed to understand their experiences and how they manage their fears. The findings revealed that survivors often feel insecure about their cancer returning, fearing an incomplete cure or relapse. To cope with these fears, participants used psychological and spiritual strategies and made lifestyle changes to improve their health and prevent recurrence. The study suggests that healthcare providers should consider these emotional and cultural factors when supporting prostate cancer survivors, encouraging open discussions about fears and providing personalized interventions to help manage FCR. Understanding these experiences can help improve care and support for prostate cancer survivors dealing with ongoing concerns about their health.

Evaluation of the Lay Summary

**Assessment Category**	**Score**	**Assessment Category**	**Score**	**Assessment Category**	**Score**
Comprehension Score	5	Gunning Fog Score	19.1	Words	135
Accuracy Score	0	SMOG Index	13.7	Complex words	34
Reading Age (years)	22.5	Coleman Liau Index	19.4	Percent of complex words	25.2
Flesch Kincaid Reading Ease	31.7	Automated Readability Index	18.0	Average words per sentence	22.5
Flesch Kincaid Grade Level	14.4	Sentences	6	Average syllables per word	1.8

Lay Summary (extended Prompt)

This study explored how prostate cancer survivors feel about the possibility of their cancer returning and how they cope with these fears. Researchers interviewed 13 men from Iran who had finished their treatment in the past two years. The study found that many survivors live with a constant sense of insecurity, fearing that their cancer might not be fully cured or could come back. To manage these fears, the men used various coping strategies, such as focusing on their mental well-being, drawing on spiritual beliefs, seeking ways to stay healthy, and making lifestyle changes. The study suggests that healthcare providers should consider the unique cultural backgrounds of prostate cancer survivors when helping them address their fears and should offer personalized support to reduce anxiety about cancer recurrence.

Evaluation of the Lay Summary

**Assessment Category**	**Score**	**Assessment Category**	**Score**	**Assessment Category**	**Score**
Comprehension Score	5	Gunning Fog Score	16.5	Words	128
Accuracy Score	0	SMOG Index	11.6	Complex words	20
Reading Age (years)	20.5	Coleman Liau Index	15.6	Percent of complex words	15.6
Flesch Kincaid Reading Ease	42.1	Automated Readability Index	16.5	Average words per sentence	25.6
Flesch Kincaid Grade Level	13.8	Sentences	5	Average syllables per word	1.6

Manuscript 49

Steed T, Chopra N, Yun J, Hill J, Burke B, Ghosh S, Warkentin B, Usmani N. Seminal Vesicle Treatment for Localized Prostate Cancer Treated with External Beam Radiotherapy. Curr Oncol. 2023 Jul 10;30(7):6587-6595. doi: 10.3390/curroncol30070483. PMID: 37504343; PMCID: PMC10377996.

Abstract

This study retrospectively reviewed data from men with localized prostate cancer treated with external beam radiotherapy (EBRT). We identified 359 men with localized prostate cancer treated with curative EBRT at the Cross Cancer Institute between 2010–2011. The volume of seminal vesicles (SVs) treated as well as dose values were extracted. These volumes were compared to gold standard contours drawn by a trained expert based on consensus European Society for Radiotherapy and Oncology (ESTRO) contouring guidelines. Patient and tumor characteristics were extracted for these patients. Memorial Sloan Kettering prostate cancer nomogram was used to assign a predicted risk of SV involvement for each patient based on baseline tumor characteristics. In patients with a predicted risk of SV involvement greater than 15% (n = 184), 86.5% (SD = 18.6) of the base of the SVs were treated with EBRT, compared to 66.7% (SD = 32.6) for patients with a predicted risk of SV involvement less than 15% (n = 175, p < 0.0001). Similarly, the mean percentage of proximal and total SV volumes treated with EBRT was 75.6% (SD = 24.4) and 68.7% (SD = 26.0) for patients with a predicted risk of SV involvement of greater than 15%, compared to 50.3% (SD = 31.0, p < 0.0001) and 41.0% (SD = 27.8, p < 0.0001) for patients with a risk of less than 15%. The results indicate that all parts of the SVs are more likely to be contoured in men with >15% risk of SV involvement than those with <15% risk. However, radiation oncologists still contour a high percentage of SVs in men with <15% risk of SV involvement, suggesting that there may be over-treatment of SVs that increases the risk of rectal or bladder toxicity.

Keywords:

prostate; radiotherapy; seminal vesicle; contour

Lay Summary (simple Prompt)

This study examines how radiation therapy for localized prostate cancer affects the seminal vesicles, which are glands near the prostate. Researchers reviewed data from 359 men treated with external beam radiotherapy (EBRT) to see how much of the seminal vesicles were included in the radiation treatment. They found that patients with a higher predicted risk of cancer involving the seminal vesicles had more of these glands targeted by radiation. However, even patients with a lower risk of involvement often had a significant portion of their seminal vesicles treated, which could lead to unnecessary exposure and increased risk of side effects like rectal or bladder damage. The study suggests that more precise targeting of radiation might reduce these risks while still effectively treating cancer, highlighting the need for tailored radiation strategies based on individual risk assessments.

Evaluation of the Lay Summary

**Assessment Category**	**Score**	**Assessment Category**	**Score**	**Assessment Category**	**Score**
Comprehension Score	5	Gunning Fog Score	20.9	Words	135
Accuracy Score	1	SMOG Index	15.0	Complex words	34
Reading Age (years)	22.5	Coleman Liau Index	16.9	Percent of complex words	25.2
Flesch Kincaid Reading Ease	25.3	Automated Readability Index	18.2	Average words per sentence	27.0
Flesch Kincaid Grade Level	16.4	Sentences	5	Average syllables per word	1.8

Lay Summary (extended Prompt)

This study looked at how radiation therapy for localized prostate cancer is targeted at the seminal vesicles, a part of the male reproductive system. Researchers reviewed the treatment of 359 men who received external beam radiotherapy (EBRT) and compared how much of the seminal vesicles were treated based on their risk of cancer involvement. They found that men with a higher risk (over 15%) of seminal vesicle involvement were more likely to have more of these tissues treated with radiation. However, even men with a lower risk (less than 15%) often had a significant portion of their seminal vesicles treated. This may lead to unnecessary radiation exposure, increasing the chances of side effects to nearby organs like the rectum and bladder. The study suggests that more precise targeting could help reduce these risks and improve patient outcomes.

Evaluation of the Lay Summary

**Assessment Category**	**Score**	**Assessment Category**	**Score**	**Assessment Category**	**Score**
Comprehension Score	5	Gunning Fog Score	16.7	Words	137
Accuracy Score	0	SMOG Index	12.0	Complex words	26
Reading Age (years)	19.5	Coleman Liau Index	14.1	Percent of complex words	19.0
Flesch Kincaid Reading Ease	40.4	Automated Readability Index	13.9	Average words per sentence	22.8
Flesch Kincaid Grade Level	13.3	Sentences	6	Average syllables per word	1.7

Manuscript 50

Elakshar S, Tolba M, Tisseverasinghe S, Pruneau L, Di Lalla V, Bahoric B, Niazi T. Salvage Whole-Pelvic Radiation and Long-Term Androgen-Deprivation Therapy in the Management of High-Risk Prostate Cancer: Long-Term Update of the McGill 0913 Study. Curr Oncol. 2023 Aug 1;30(8):7252-7262. doi: 10.3390/curroncol30080526. PMID: 37623007; PMCID: PMC10453184.

Abstract

Purpose: To report the long-term outcomes of the McGill 0913 study and the potential benefits of combining prostate-bed radiotherapy (PBRT), pelvic-lymph-node radiotherapy (PLNRT), and long term ADT (LT-ADT). Materials and Methods: From 2010 to 2016, 46 high-risk prostate cancer patients who experienced biochemical recurrence (BCR) after radical prostatectomy (RP) were enrolled in this single-arm phase II clinical trial. The patients were eligible if they had a Gleason score > 8, locally advanced disease (≥pT3), a preoperative PSA of >20 ng/mL, or positive lymph nodes (LN). The patients were treated with a combination of 24 months of ADT, PBRT, and PLNRT. The primary outcome was biochemical progression-free survival (bPFS) and the predefined secondary endpoints included distant-metastasis-free survival (DMFS), overall survival (OS), and toxicity. In this update, we also report the median follow-up of 8.8 years and 10 years OS. Results: At a median follow-up of 8.8 years, 43 patients were eligible for analysis. The median pre-salvage PSA was 0.30 μg/L. Half (51%) of the patients (n = 22) had positive margins, 40% (n = 17) had Gleason scores > 8, 63% (n = 27) had extracapsular extension, 42% (n = 18) had seminal vesicle invasion, and 19% (n = 8) had LN involvement. The 10-year bPFS was 68.3 %. The 10-year DMFS was 72.9%. The 10-year OS was 97%. There were two non-cancer-related deaths. The first patient died of congestive heart failure while the other died of an unknown cause. No new toxicity was observed after the initial report. Conclusions: Our study demonstrates that treatment escalation with PBRT, PLNRT, and LT-ADT improves long term outcomes. In view of the recently published SPPORT study, we conclude that this novel approach of treatment intensification in high-risk post-prostatectomy patients is safe and effective, and that it should be offered as the standard of care.

Keywords:

prostate; cancer; radiotherapy; hormonal therapy; salvage; radical prostatectomy

<u>Lay Summary (simple Prompt)</u>

This study evaluates the long-term effectiveness of combining whole pelvic radiation, prostate-bed radiation, and long-term hormone therapy for treating high-risk prostate cancer patients who experience cancer recurrence after surgery. Involving 46 patients, the research aimed to determine how well this combined treatment approach prevents cancer progression and improves survival rates. After a median follow-up of 8.8 years, the study found that 68.3% of patients remained free from biochemical progression, and 72.9% had no distant metastasis. The overall survival rate was 97% at ten years, with no new side effects reported. The findings suggest that this comprehensive treatment strategy is both safe and effective, offering a promising option for improving outcomes in high-risk prostate cancer patients who have undergone surgery. This study supports the use of intensified treatment as a standard approach for managing recurrent prostate cancer.

<u>Evaluation of the Lay Summary</u>

**Assessment Category**	**Score**	**Assessment Category**	**Score**	**Assessment Category**	**Score**
Comprehension Score	5	Gunning Fog Score	15.0	Words	145
Accuracy Score	0	SMOG Index	10.8	Complex words	31
Reading Age (years)	18.5	Coleman Liau Index	16.0	Percent of complex words	21.4
Flesch Kincaid Reading Ease	41.1	Automated Readability Index	21.1	Average words per sentence	16.1
Flesch Kincaid Grade Level	11.5	Sentences	9	Average syllables per word	1.8

Lay Summary (extended Prompt)

This study looked at a treatment plan for men with high-risk prostate cancer who had a return of the disease after surgery. The treatment combined radiation therapy to both the area where the prostate was removed and the pelvic lymph nodes, along with long-term hormone therapy. The researchers followed 46 patients for nearly nine years to see how well this approach worked. They found that 68% of the patients had no signs of cancer recurrence for 10 years, and 73% were free from distant metastasis. Overall survival was very high, with 97% of patients still alive after 10 years. The treatment was well-tolerated, with no new side effects reported. These findings suggest that this combined treatment is a safe and effective option for men with high-risk prostate cancer and could be considered as a standard care approach.

Evaluation of the Lay Summary

**Assessment Category**	**Score**	**Assessment Category**	**Score**	**Assessment Category**	**Score**
Comprehension Score	5	Gunning Fog Score	11.7	Words	141
Accuracy Score	0	SMOG Index	8.0	Complex words	13
Reading Age (years)	15.5	Coleman Liau Index	11.8	Percent of complex words	9.2
Flesch Kincaid Reading Ease	62.2	Automated Readability Index	10.7	Average words per sentence	20.1
Flesch Kincaid Grade Level	9.6	Sentences	7	Average syllables per word	1.5

Manuscript 51

Hong S, Kim SH, Yoo B, Kim JY. Deep Learning Algorithm for Tumor Segmentation and Discrimination of Clinically Significant Cancer in Patients with Prostate Cancer. Curr Oncol. 2023 Aug 1;30(8):7275-7285. doi: 10.3390/curroncol30080528. PMID: 37623009; PMCID: PMC10453750.

Abstract

Background: We investigated the feasibility of a deep learning algorithm (DLA) based on apparent diffusion coefficient (ADC) maps for the segmentation and discrimination of clinically significant cancer (CSC, Gleason score ≥ 7) from non-CSC in patients with prostate cancer (PCa). Methods: Data from a total of 149 consecutive patients who had undergone 3T-MRI and been pathologically diagnosed with PCa were initially collected. The labelled data (148 images for GS6, 580 images for GS7) were applied for tumor segmentation using a convolutional neural network (CNN). For classification, 93 images for GS6 and 372 images for GS7 were used. For external validation, 22 consecutive patients from five different institutions (25 images for GS6, 70 images for GS7) representing different MR machines were recruited. Results: Regarding segmentation and classification, U-Net and DenseNet were used, respectively. The tumor Dice scores for internal and external validation were 0.822 and 0.7776, respectively. As for classification, the accuracies of internal and external validation were 73 and 75%, respectively. For external validation, diagnostic predictive values for CSC (sensitivity, specificity, positive predictive value and negative predictive value) were 84, 48, 82 and 52%, respectively. Conclusions: Tumor segmentation and discrimination of CSC from non-CSC is feasible using a DLA developed based on ADC maps (b2000) alone.

Keywords:

magnetic resonance imaging (MRI); diffusion-weighted imaging (DWI); prostate cancer; Gleason score; deep learning

Lay Summary (simple Prompt)

This study explores the use of a deep learning algorithm to improve the detection and diagnosis of significant prostate cancer. The researchers developed a computer model that analyzes MRI images to distinguish between more aggressive cancers (with a Gleason score of 7 or higher) and less serious cases. Using data from patients with prostate cancer, the model successfully identified tumors and classified them as significant or non-significant with an accuracy of around 73-75%. The results show that this technology could help doctors more accurately identify and assess prostate cancer using imaging alone, potentially leading to better treatment decisions. This research highlights the potential of artificial intelligence to enhance cancer diagnosis and improve outcomes for patients by providing a more precise, non-invasive assessment tool

Evaluation of the Lay Summary

**Assessment Category**	**Score**	**Assessment Category**	**Score**	**Assessment Category**	**Score**
Comprehension Score	5	Gunning Fog Score	19.6	Words	126
Accuracy Score	1	SMOG Index	14.1	Complex words	30
Reading Age (years)	22.5	Coleman Liau Index	17.3	Percent of complex words	23.8
Flesch Kincaid Reading Ease	22.8	Automated Readability Index	17.6	Average words per sentence	25.2
Flesch Kincaid Grade Level	16.3	Sentences	5	Average syllables per word	1.9

Lay Summary (extended Prompt)

This study explores the use of a deep learning computer program to help identify and classify significant prostate cancer using MRI scans. The researchers developed a tool that uses special images called apparent diffusion coefficient (ADC) maps to distinguish between more aggressive cancers (Gleason score 7 or higher) and less aggressive ones. They tested the tool with data from patients who had already been diagnosed with prostate cancer and then validated it using data from different hospitals. The program was quite accurate, correctly identifying aggressive cancers in about 75% of cases. This technology could help doctors better detect and treat prostate cancer, potentially improving patient outcomes by focusing on more significant cancers while reducing unnecessary treatments for less harmful cases.

Evaluation of the Lay Summary

**Assessment Category**	**Score**	**Assessment Category**	**Score**	**Assessment Category**	**Score**
Comprehension Score	5	Gunning Fog Score	18.9	Words	120
Accuracy Score	0	SMOG Index	13.6	Complex words	28
Reading Age (years)	22.5	Coleman Liau Index	17.6	Percent of complex words	23.3
Flesch Kincaid Reading Ease	28.8	Automated Readability Index	17.3	Average words per sentence	24.0
Flesch Kincaid Grade Level	15.2	Sentences	5	Average syllables per word	1.8

Manuscript 52

Hoshi S, Yaginuma K, Meguro S, Onagi A, Matsuoka K, Hata J, Sato Y, Akaihata H, Kataoka M, Ogawa S, Uemura M, Kojima Y. PSMA Targeted Molecular Imaging and Radioligand Therapy for Prostate Cancer: Optimal Patient and Treatment Issues. Curr Oncol. 2023 Aug 1;30(8):7286-7302. doi: 10.3390/curroncol30080529. PMID: 37623010; PMCID: PMC10453875.

Abstract

Theranostics (therapy + diagnosis) targeting prostate-specific membrane antigen (PSMA) is an emerging therapeutic modality that could alter treatment strategies for prostate cancer. Although PSMA-targeted radioligand therapy (PSMA-RLT) has a highly therapeutic effect on PSMA-positive tumor tissue, the efficacy of PSMA-RLT depends on PSMA expression. Moreover, predictors of treatment response other than PSMA expression are under investigation. Therefore, the optimal patient population for PSMA-RLT remains unclear. This review provides an overview of the current status of theranostics for prostate cancer, focusing on PSMA ligands. In addition, we summarize various findings regarding the efficacy and problems of PSMA-RLT and discuss the optimal patient for PSMA-RLT.

Keywords:

theranostics; PSMA; prostate cancer; molecular imaging; radioligand therapy

Lay Summary (simple Prompt)

This study reviews the use of a new approach called theranostics, which combines therapy and diagnosis to treat prostate cancer by targeting a specific protein on cancer cells called PSMA. PSMA-targeted radioligand therapy (PSMA-RLT) can effectively treat tumors that express this protein, but its success varies based on how much PSMA is present on the cancer cells. The study discusses the current knowledge about PSMA-RLT, including its effectiveness and challenges, and explores which patients might benefit most from this treatment. The findings suggest that while PSMA-RLT shows promise, more research is needed to identify which patients are best suited for this therapy and to understand other factors that may predict treatment response. This research could help refine prostate cancer treatments, offering more personalized and effective options for patients.

Evaluation of the Lay Summary

Assessment Category	Score	Assessment Category	Score	Assessment Category	Score
Comprehension Score	5	Gunning Fog Score	16.6	Words	132
Accuracy Score	0	SMOG Index	11.6	Complex words	20
Reading Age (years)	20.5	Coleman Liau Index	16.5	Percent of complex words	15.1
Flesch Kincaid Reading Ease	37.8	Automated Readability Index	17.6	Average words per sentence	26.4
Flesch Kincaid Grade Level	14.6	Sentences	5	Average syllables per word	1.7

Lay Summary (extended Prompt)

This study looks at a new approach to treating prostate cancer using a technique called theranostics, which combines therapy and diagnosis. The focus is on a method that targets a specific protein found on prostate cancer cells, known as PSMA. By using special drugs that attach to PSMA, doctors can both detect and treat cancer more effectively. However, the success of this treatment depends on how much PSMA is present in the tumor. The study reviews current knowledge about this treatment, including how well it works and which patients might benefit the most. Understanding these factors can help doctors make better decisions about using this therapy and improve outcomes for patients with prostate cancer.

Evaluation of the Lay Summary

**Assessment Category**	**Score**	**Assessment Category**	**Score**	**Assessment Category**	**Score**
Comprehension Score	5	Gunning Fog Score	11.8	Words	114
Accuracy Score	1	SMOG Index	8.3	Complex words	12
Reading Age (years)	17.5	Coleman Liau Index	14.6	Percent of complex words	10.5
Flesch Kincaid Reading Ease	52.5	Automated Readability Index	12.4	Average words per sentence	19.0
Flesch Kincaid Grade Level	10.7	Sentences	6	Average syllables per word	1.6

Manuscript 53

Sciarra A, Santarelli V, Santodirocco L, Frisenda M, Salciccia S, Casale P, Forte F, Mariotti G, Moriconi M, Cattarino S, Sciarra B, Bevilacqua G, Gentilucci A. Is It Time to Anticipate the Use of PARP Inhibition in Prostate Cancer Patients? Curr Oncol. 2023 Aug 30;30(9):8054-8067. doi: 10.3390/curroncol30090584. PMID: 37754499; PMCID: PMC10528501.

Abstract

The increasing diffusion of genetic analysis regarding the pathogenetic variants (PVs) of genes involved in DNA Damage Repair (DDR) mechanisms and the development of Poly ADP ribose polymerase (PARP) inhibitors (PARPis) led to the first valid precision medicine option tailored toward metastatic prostate cancer (mPC). The concept of anticipation in the systemic treatment of mPC was initially adopted for androgen receptor signaling inhibitors (ARSIs) to describe the expansion of their indications, from the setting of the late-stage second-line treatment of metastatic castration-resistant prostate cancer (mCRPC) to first-line therapy in selected cases. There is already mounting evidence in favor of the anticipation of PARPis in the first line of mCRPC therapy, and further evidence in favor of mHSPC is emerging. Many studies have demonstrated the synergism between ARSIs and PARP inhibitors. Recent discoveries regarding the crosstalk between the androgen receptor (AR) and DNA repair mechanisms are disconnecting the use of PARPis from genetic analysis. The new message emerging is that the combination of PARPis with ARSIs may work independently of DDR mutational status. As a matter of fact, most of the recent trials analyzing the combination of PARPis with abiraterone or enzalutamide as a first-line therapy enrolled mCRPC patients irrespective of their mutational status. The PROPEL trial concluded that the advantage of the combination was independent of PV status, despite a higher advantage being reported in the BRCA1/2 mutated subgroup. The MAGNITUDE trial, however, showed a significant advantage only in the DDR mutated subgroup, and the DDR non-mutated cohort was closed for further enrollment. The combination of PARPis with ARSIs represents a significant strategy with a view to the anticipation and intensification of care in mPC. However, it should not nullify the advantages of precision medicine linked to the genetic analysis of DDR genes.

Keywords:

prostate cancer; PARP inhibitors; DDR gene; CRPC

Lay Summary (simple Prompt)

This study explores the potential benefits of using PARP inhibitors earlier in the treatment of metastatic prostate cancer. PARP inhibitors, which target cancer cells with specific DNA repair defects, are currently used later in treatment after other therapies. However, new research suggests they could be effective as a first-line treatment, particularly when combined with androgen receptor inhibitors, which are another type of prostate cancer therapy. Some studies show that this combination works well regardless of specific genetic mutations, while others indicate it is more effective in patients with certain gene mutations. The findings highlight the potential for expanding the use of PARP inhibitors to provide more personalized and effective treatment options for prostate cancer patients earlier in their care, improving outcomes by tailoring treatments to the cancer's genetic characteristics.

Evaluation of the Lay Summary

**Assessment Category**	**Score**	**Assessment Category**	**Score**	**Assessment Category**	**Score**
Comprehension Score	5	Gunning Fog Score	21.2	Words	130
Accuracy Score	1	SMOG Index	15.2	Complex words	35
Reading Age (years)	23.5	Coleman Liau Index	18.9	Percent of complex words	27.0
Flesch Kincaid Reading Ease	21.0	Automated Readability Index	19.3	Average words per sentence	26.0
Flesch Kincaid Grade Level	16.8	Sentences	5	Average syllables per word	1.9

Lay Summary (extended Prompt)

This study explores whether it might be beneficial to use a type of medication called PARP inhibitors earlier in treating prostate cancer, especially for advanced cases. PARP inhibitors help repair damaged DNA in cancer cells, and their use has shown promise when combined with other prostate cancer treatments that target hormone signals. The research suggests that using PARP inhibitors earlier, rather than waiting until later stages of cancer, could improve outcomes for some patients. However, the results are mixed: while some studies found benefits regardless of a patient's specific genetic makeup, others saw advantages only in those with certain genetic mutations. The findings highlight a potential shift towards more personalized and intensified treatment strategies for prostate cancer, emphasizing the importance of genetic testing to guide therapy choices.

Evaluation of the Lay Summary

**Assessment Category**	**Score**	**Assessment Category**	**Score**	**Assessment Category**	**Score**
Comprehension Score	4	Gunning Fog Score	17.4	Words	127
Accuracy Score	0	SMOG Index	12.4	Complex words	23
Reading Age (years)	21.5	Coleman Liau Index	18.1	Percent of complex words	18.1
Flesch Kincaid Reading Ease	31.2	Automated Readability Index	18.4	Average words per sentence	25.4
Flesch Kincaid Grade Level	15.2	Sentences	5	Average syllables per word	1.8

Manuscript 54

Burge L, Ilie G, MacDonald C, Riel H, Rutledge RDH. Changes in Stress Reduction Following a 28-Day Prostate Cancer Patient Empowerment Program (PC-PEP) among Prostate Cancer Survivors. Curr Oncol. 2023 Aug 29;30(9):7936-7949. doi: 10.3390/curroncol30090577. PMID: 37754492; PMCID: PMC10528192.

Abstract

Prostate cancer (PCa) survivors often experience post-treatment challenges that impact their well-being and mental health. The Prostate Cancer Patient Empowerment Program (PC-PEP) aims to address these issues through a comprehensive intervention, involving daily meditation/breathing exercises, physical activity, pelvic floor exercises, emotional connection strategies, and peer support. This study presents a secondary analysis of a Phase 2 feasibility study that evaluated the effects of a 28-day PC-PEP intervention on stress reduction. Thirty patients with PCa from the Maritimes, Canada, underwent pre- and post-intervention assessments to measure brainwave activity (delta, theta, alpha, beta, and gamma) using the Muse™ headband, and heart rate variability (HRV) using the HeartMath® Inner Balance™ as indicators of stress reduction. A statistically significant Time × Sensor Scalp Assessment Time interaction emerged for all brain waves. Amplitudes were generally higher during the first half of the meditation assessment time but became comparable afterward. A statistically significant Time × Sensor Scalp Location × Sensor Scalp Assessment Time interaction also emerged for alpha waves, indicating higher prefrontal lobe amplitudes than temporal lobe amplitudes from pre- to post-assessment. There were no statistically significant differences in HRV metrics from pre- to post-intervention, except for a marginally significant achievement score, indicating increased HRV coherence post-intervention. The findings suggest that the stress reduction component of PC-PEP successfully improved outcomes related to decreased stress. These results have implications for the development of future iterations of PC-PEP interventions, aiming to optimize participant benefits.

Keywords:

prostate cancer; stress reduction; heart rate variability; brain wave activity; PC-PEP; prostate cancer survivorship

Lay Summary (simple Prompt)

This study examines how a 28-day Prostate Cancer Patient Empowerment Program (PC-PEP) can help reduce stress among prostate cancer survivors. The program includes daily activities such as meditation, breathing exercises, physical activity, and emotional support strategies. Thirty participants from Canada were assessed before and after the program using devices that measure brainwave activity and heart rate variability, both indicators of stress levels. The results showed significant improvements in brainwave patterns associated with relaxation, particularly in the prefrontal brain region, suggesting reduced stress. While there were no major changes in heart rate variability, there was a slight improvement in heart rate coherence, indicating better stress management. These findings suggest that the PC-PEP program effectively reduces stress, which could improve the well-being of prostate cancer survivors. This research supports further development of such programs to enhance mental health and quality of life for cancer survivors.

Evaluation of the Lay Summary

**Assessment Category**	**Score**	**Assessment Category**	**Score**	**Assessment Category**	**Score**
Comprehension Score	5	Gunning Fog Score	16.6	Words	147
Accuracy Score	1	SMOG Index	12.4	Complex words	32
Reading Age (years)	21.5	Coleman Liau Index	19.3	Percent of complex words	21.8
Flesch Kincaid Reading Ease	25.0	Automated Readability Index	17.2	Average words per sentence	21.0
Flesch Kincaid Grade Level	15.0	Sentences	7	Average syllables per word	1.9

Lay Summary (extended Prompt)

This study looked at how a 28-day program called the Prostate Cancer Patient Empowerment Program (PC-PEP) can help reduce stress in men who have survived prostate cancer. The program included daily meditation and breathing exercises, physical activity, pelvic floor exercises, emotional support, and peer connections. Thirty prostate cancer survivors from Canada participated in this program, and their stress levels were measured before and after using devices that track brain activity and heart rate. The study found that the program significantly reduced stress, as shown by changes in brain wave patterns and a small improvement in heart rate variability. These results suggest that the PC-PEP program can be effective in helping prostate cancer survivors manage stress and improve their overall well-being. This study could lead to better programs for supporting men's mental and emotional health after cancer treatment.

Evaluation of the Lay Summary

**Assessment Category**	**Score**	**Assessment Category**	**Score**	**Assessment Category**	**Score**
Comprehension Score	5	Gunning Fog Score	14.8	Words	141
Accuracy Score	0	SMOG Index	10.8	Complex words	21
Reading Age (years)	19.5	Coleman Liau Index	16.3	Percent of complex words	14.9
Flesch Kincaid Reading Ease	40.8	Automated Readability Index	16.0	Average words per sentence	23.5
Flesch Kincaid Grade Level	13.4	Sentences	6	Average syllables per word	1.7

Manuscript 55

Vavassori A, Mauri G, Mazzola GC, Mastroleo F, Bonomo G, Durante S, Zerini D, Marvaso G, Corrao G, Ferrari ED, Rondi E, Vigorito S, Cattani F, Orsi F, Jereczek-Fossa BA. Cyberknife Radiosurgery for Prostate Cancer after Abdominoperineal Resection (CYRANO): The Combined Computer Tomography and Electromagnetic Navigation Guided Transperineal Fiducial Markers Implantation Technique. Curr Oncol. 2023 Aug 28;30(9):7926-7935. doi: 10.3390/curroncol30090576. PMID: 37754491; PMCID: PMC10529393.

Abstract

In this technical development report, we present the strategic placement of fiducial markers within the prostate under the guidance of computed tomography (CT) and electromagnetic navigation (EMN) for the delivery of ultra-hypofractionated cyberknife (CK) therapy in a patient with localized prostate cancer (PCa) who had previously undergone chemo-radiotherapy for rectal cancer and subsequent abdominoperineal resection due to local recurrence. The patient was positioned in a prone position with a pillow under the pelvis to facilitate access, and an electromagnetic fiducial marker was placed on the patient's skin to establish a stable position. CT scans were performed to plan the procedure, mark virtual points, and simulate the needle trajectory using the navigation system. Local anesthesia was administered, and a 21G needle was used to place the fiducial markers according to the navigation system information. A confirmatory CT scan was obtained to ensure proper positioning. The implantation procedure was safe, without any acute side effects such as pain, hematuria, dysuria, or hematospermia. Our report highlights the ability to use EMN systems to virtually navigate within a pre-acquired imaging dataset in the interventional room, allowing for non-conventional approaches and potentially revolutionizing fiducial marker positioning, offering new perspectives for PCa treatment in selected cases.

Keywords:

cyberknife; radiosurgery; prostate cancer; rectal cancer; abdominoperineal resection; Lynch Syndrome

Lay Summary (simple Prompt)

This study describes a new technique for placing fiducial markers in the prostate to guide targeted radiosurgery for prostate cancer in patients who have previously had surgery and treatment for rectal cancer. Using a combination of computed tomography (CT) and electromagnetic navigation (EMN), doctors accurately positioned the markers to help deliver precise CyberKnife therapy, a type of highly focused radiation treatment. The procedure was safely performed without any immediate side effects, such as pain or bleeding. This innovative approach allows for more accurate targeting of the prostate cancer, even in complex cases where standard methods are not possible. The findings suggest that this technique could improve the precision and effectiveness of radiosurgery for prostate cancer, offering new treatment options for patients with a history of other cancers or surgeries.

Evaluation of the Lay Summary

Assessment Category	Score	Assessment Category	Score	Assessment Category	Score
Comprehension Score	4	Gunning Fog Score	19.3	Words	129
Accuracy Score	1	SMOG Index	14.1	Complex words	30
Reading Age (years)	22.5	Coleman Liau Index	18.1	Percent of complex words	23.3
Flesch Kincaid Reading Ease	17.4	Automated Readability Index	18.6	Average words per sentence	25.8
Flesch Kincaid Grade Level	17.2	Sentences	5	Average syllables per word	1.9

Lay Summary (extended Prompt)

This study explores a new method for placing small markers inside the prostate to help guide a type of precise radiation therapy called Cyberknife, used for treating prostate cancer. The patient in this study had already undergone surgery and radiation for rectal cancer, which made the usual methods of placing these markers difficult. To solve this, the researchers used a combination of CT scans and an electromagnetic navigation system to guide the placement of the markers. The procedure was done safely, without causing any pain or side effects. This new technique could improve the accuracy of radiation therapy for prostate cancer patients, especially for those who have had previous surgeries or treatments, offering a new approach to treatment in complex cases.

Evaluation of the Lay Summary

**Assessment Category**	**Score**	**Assessment Category**	**Score**	**Assessment Category**	**Score**
Comprehension Score	5	Gunning Fog Score	17.0	Words	121
Accuracy Score	0	SMOG Index	12.4	Complex words	23
Reading Age (years)	20.5	Coleman Liau Index	15.3	Percent of complex words	19.0
Flesch Kincaid Reading Ease	35.4	Automated Readability Index	15.6	Average words per sentence	24.2
Flesch Kincaid Grade Level	14.3	Sentences	5	Average syllables per word	1.7

Manuscript 56

Numakura K, Kobayashi M, Muto Y, Sato H, Sekine Y, Sobu R, Aoyama Y, Takahashi Y, Okada S, Sasagawa H, Narita S, Kumagai S, Wada Y, Mori N, Habuchi T. The Current Trend of Radiation Therapy for Patients with Localized Prostate Cancer. Curr Oncol. 2023 Sep 1;30(9):8092-8110. doi: 10.3390/curroncol30090587. PMID: 37754502; PMCID: PMC10529045.

Abstract

A recent approach to radiotherapy for prostate cancer is the administration of high doses of radiation to the prostate while minimizing the risk of side effects. Thus, image-guided radiotherapy utilizes advanced imaging techniques and is a feasible strategy for increasing the radiation dose. New radioactive particles are another approach to achieving high doses and safe procedures. Prostate brachytherapy is currently considered as a combination therapy. Spacers are useful to protect adjacent organs, specifically the rectum, from excessive radiation exposure.

Keywords:

prostate cancer; radiation therapy; intensity-modulated radiation therapy; image-guided radiation therapy; volumetric modulated arc therapy; stereotactic body radiation therapy; brachytherapy; metastasis direct therapy; hydrogel spacer

Lay Summary (simple Prompt)

This study reviews the latest advancements in radiation therapy for treating localized prostate cancer, focusing on methods that deliver high doses of radiation directly to the prostate while minimizing side effects. Techniques such as image-guided radiotherapy use advanced imaging to precisely target the cancer, allowing for higher doses of radiation to be safely administered. New types of radioactive particles and the use of spacers, which protect nearby organs like the rectum from excessive radiation, are also explored as ways to improve treatment safety and effectiveness. Prostate brachytherapy, a form of internal radiation, is now often combined with other therapies for better outcomes. These advancements aim to improve the precision and safety of radiation therapy, offering more effective treatment options for patients with localized prostate cancer.

Evaluation of the Lay Summary

Assessment Category	Score	Assessment Category	Score	Assessment Category	Score
Comprehension Score	5	Gunning Fog Score	20.9	Words	126
Accuracy Score	1	SMOG Index	15.0	Complex words	34
Reading Age (years)	23.5	Coleman Liau Index	18.4	Percent of complex words	27.0
Flesch Kincaid Reading Ease	15.4	Automated Readability Index	18.5	Average words per sentence	25.2
Flesch Kincaid Grade Level	17.4	Sentences	5	Average syllables per word	2.0

Lay Summary (extended Prompt)

This study looks at new ways to use radiation therapy to treat localized prostate cancer. The goal is to deliver high doses of radiation directly to the prostate to kill cancer cells while reducing the risk of side effects. The study discusses advanced techniques like image-guided radiotherapy, which uses detailed imaging to target the cancer more precisely. It also looks at the use of new types of radioactive particles and a method called brachytherapy, which involves placing radiation sources inside or near the tumor. To protect nearby organs, such as the rectum, from being exposed to too much radiation, special materials called spacers can be used. These approaches aim to make radiation therapy safer and more effective for patients with prostate cancer. This research helps doctors better understand how to improve treatment options for prostate cancer patients.

Evaluation of the Lay Summary

**Assessment Category**	**Score**	**Assessment Category**	**Score**	**Assessment Category**	**Score**
Comprehension Score	5	Gunning Fog Score	14.6	Words	138
Accuracy Score	0	SMOG Index	10.5	Complex words	23
Reading Age (years)	18.5	Coleman Liau Index	15.2	Percent of complex words	16.7
Flesch Kincaid Reading Ease	44.0	Automated Readability Index	13.2	Average words per sentence	19.7
Flesch Kincaid Grade Level	12.0	Sentences	7	Average syllables per word	1.7

Manuscript 57

Karim S, Lowther J, Gyulay G, O'Sullivan D, Wallis CJD, Yip SM, Brenner DR, Boyne DJ, Cheung WY. A Real-World Evidence Study Using Alberta-Population-Based Data to Describe Treatment Patterns for Metastatic Castration-Sensitive Prostate Cancer Patients (AWARENESS). Curr Oncol. 2023 Sep 1;30(9):8149-8158. doi: 10.3390/curroncol30090591. PMID: 37754506; PMCID: PMC10528640.

Abstract

Introduction: Over the past decade, the treatment of metastatic castration-sensitive prostate cancer (mCSPC) has changed significantly. Current guidelines suggest the use of androgen deprivation therapy (ADT) plus an additional systemic therapy, regardless of disease burden and risk, based on phase 1 evidence showing improved overall survival. We sought to describe treatment patterns of patients with mCSPC in the province of Alberta. Methods: This was a retrospective, population-based, cohort study of male patients aged ≥18 with mCSPC at the time of diagnosis and who initiated ADT between 1 January 2016 and 31 December 2020. Data were obtained from the Alberta Cancer Registry. Patients were assigned to an ADT-alone cohort or a treatment intensification cohort (cohorts 2–5). The primary objectives of this study were to describe baseline characteristics and the treatment of mCSPC patients who initiated ADT with or without treatment intensification. Overall survival between cohorts was a secondary objective. Descriptive statistics were used to describe differences in baseline characteristics of each cohort. Overall survival was calculated using the Kaplan–Meier method. All statistical tests were two-sided and are used to call out likely cohort differences descriptively. Results: Between 1 January 2016 and 31 December 2020, we identified a total of 960 patients with mCSPC (median age 74 years, IQR 66–82). Most patients received ADT alone (67%), followed by ADT plus abiraterone (18%), ADT plus docetaxel (12%), and ADT plus enzalutamide or apalutamide (3%). Over the study period, we observed an increase in the utilization of treatment intensification over time, in particular, the increased use of androgen-receptor-axis-targeted (ARAT) therapies. Patients who received ADT alone were older, were more likely to have more than one comorbid condition, had fewer sites of metastatic disease, and were less likely to be on opioid medications. Conclusions: In this study, we show that patients who received ADT alone

as treatment for mCSPC are older, have more comorbidities, and have less extensive disease. While there has been a decline over time in the number of patients treated with ADT alone, over 50% of all patients with mCSPC continue to receive ADT alone. Further work is needed to understand barriers to treatment intensification and for knowledge translation initiatives to improve the treatment of patients with mCSPC.

Keywords:
metastatic hormone-sensitive prostate cancer; treatment intensification; real-world evidence

Lay Summary (simple Prompt)

This study investigates the treatment patterns for patients with metastatic castration-sensitive prostate cancer (mCSPC) in Alberta, Canada. It analyzes how these patients are treated, particularly focusing on those who receive only androgen deprivation therapy (ADT) versus those who receive ADT combined with additional therapies. The findings reveal that most patients (67%) were treated with ADT alone, particularly older patients with more health conditions and less extensive cancer spread. However, there has been an increase in the use of additional therapies over time, especially new targeted drugs. The study highlights that while treatment approaches are evolving, many patients still receive ADT alone, which may not be the most effective option for everyone. These insights suggest the need for better understanding and overcoming barriers to more intensive treatment to improve patient outcomes in this group.

Evaluation of the Lay Summary

Assessment Category	Score	Assessment Category	Score	Assessment Category	Score
Comprehension Score	5	Gunning Fog Score	17.0	Words	134
Accuracy Score	0	SMOG Index	12.7	Complex words	29
Reading Age (years)	21.5	Coleman Liau Index	18.0	Percent of complex words	21.6
Flesch Kincaid Reading Ease	30.8	Automated Readability Index	16.8	Average words per sentence	22.3
Flesch Kincaid Grade Level	14.5	Sentences	6	Average syllables per word	1.8

Lay Summary (extended Prompt)

This study looked at how men with metastatic prostate cancer that still responds to hormone therapy are being treated in Alberta, Canada. Researchers examined data from 960 patients who started hormone therapy (ADT) between 2016 and 2020. They found that most patients were treated with ADT alone, but some also received additional treatments like abiraterone, docetaxel, or other drugs that target cancer cells. The study showed that patients who only received ADT were typically older and had other health problems. Over time, there was an increase in using more intensive treatment options. However, more than half of the patients still received ADT alone. The findings suggest a need for better understanding of why more aggressive treatments are not used more widely and how to improve treatment strategies for these patients.

Evaluation of the Lay Summary

**Assessment Category**	**Score**	**Assessment Category**	**Score**	**Assessment Category**	**Score**
Comprehension Score	5	Gunning Fog Score	11.7	Words	130
Accuracy Score	0	SMOG Index	8.8	Complex words	16
Reading Age (years)	17.5	Coleman Liau Index	14.8	Percent of complex words	12.3
Flesch Kincaid Reading Ease	49.4	Automated Readability Index	12.3	Average words per sentence	18.6
Flesch Kincaid Grade Level	11.0	Sentences	7	Average syllables per word	1.6

Manuscript 58

Utsumi T, Suzuki H, Ishikawa H, Wakatsuki M, Okonogi N, Harada M, Ichikawa T, Akakura K, Murakami Y, Tsuji H, Yamada S. Identification of Early Biochemical Recurrence Predictors in High-Risk Prostate Cancer Patients Treated with Carbon-Ion Radiotherapy and Androgen Deprivation Therapy. Curr Oncol. 2023 Sep 27;30(10):8815-8825. doi: 10.3390/curroncol30100636. PMID: 37887536; PMCID: PMC10605605.

Abstract

The aim of this retrospective study was to identify clinical predictors of early biochemical recurrence (BCR) in patients with high-risk prostate cancer (PCa) treated with carbon-ion radiotherapy (CIRT) and androgen deprivation therapy (ADT). A total of 670 high-risk PCa patients treated with CIRT and ADT were included in the study. Early BCR was defined as recurrence occurring during adjuvant ADT after CIRT or within 2 years after completion of ADT. Univariate and multivariate analyses were performed to identify clinical predictors of early BCR. Patients were also classified according to the Systemic Therapy in Advancing or Metastatic Prostate cancer (STAMPEDE) PCa classification. Early BCR was observed in 5.4% of the patients. Multivariate analysis identified clinical T3b stage and ≥75% positive biopsy cores as clinical predictors of early BCR after CIRT and ADT. The STAMPEDE PCa classification was also significantly associated with early BCR based on univariate analysis. These predictors can help clinicians identify patients who are at risk of early BCR. In the future, combination therapy of ADT with abiraterone may be an option for high-risk PCa patients who are at risk of early BCR, based on the results of the STAMPEDE study.

Keywords:

biochemical recurrence; early recurrence; prostate cancer; high-risk; carbon-ion radiotherapy; androgen deprivation therapy

Lay Summary (simple Prompt)

This study aims to identify early signs that predict the return of prostate cancer in high-risk patients who have been treated with carbon-ion radiotherapy (a type of advanced radiation) and hormone therapy. By analyzing data from 670 patients, the researchers found that those with advanced tumor stage (T3b) or a high percentage of cancer in their biopsy samples (75% or more) were more likely to experience early biochemical recurrence, meaning the cancer might return soon after treatment. The study also found that a specific classification system for prostate cancer could help identify patients at higher risk of recurrence. These findings can help doctors better predict which patients are more likely to have a recurrence and consider additional treatments, like combining hormone therapy with abiraterone, to improve outcomes for those at high risk.

Evaluation of the Lay Summary

**Assessment Category**	**Score**	**Assessment Category**	**Score**	**Assessment Category**	**Score**
Comprehension Score	3	Gunning Fog Score	19.4	Words	134
Accuracy Score	1	SMOG Index	12.9	Complex words	20
Reading Age (years)	22.5	Coleman Liau Index	15.2	Percent of complex words	15.0
Flesch Kincaid Reading Ease	28.9	Automated Readability Index	20.1	Average words per sentence	33.5
Flesch Kincaid Grade Level	17.6	Sentences	4	Average syllables per word	1.7

Lay Summary (extended Prompt)

This study aimed to find early signs of prostate cancer returning in high-risk patients who were treated with carbon-ion radiotherapy (CIRT) and androgen deprivation therapy (ADT). Researchers looked at 670 patients and identified key factors that might predict cancer returning within two years after treatment. They found that patients with a more advanced cancer stage (T3b) or those with more than 75% of biopsy samples showing cancer were more likely to have an early recurrence. Another classification system, called STAMPEDE, was also linked to early recurrence. Identifying these risk factors helps doctors know which patients may need additional treatments, like combining ADT with other drugs such as abiraterone, to reduce the chance of cancer coming back. This information is important for improving treatment plans and outcomes for high-risk prostate cancer patients.

Evaluation of the Lay Summary

**Assessment Category**	**Score**	**Assessment Category**	**Score**	**Assessment Category**	**Score**
Comprehension Score	5	Gunning Fog Score	14.9	Words	134
Accuracy Score	0	SMOG Index	10.6	Complex words	20
Reading Age (years)	19.5	Coleman Liau Index	16.0	Percent of complex words	14.9
Flesch Kincaid Reading Ease	42.7	Automated Readability Index	15.2	Average words per sentence	22.3
Flesch Kincaid Grade Level	12.8	Sentences	6	Average syllables per word	1.7

Manuscript 59

Ilie G, MacDonald C, Richman H, Rendon R, Mason R, Nuyens A, Bailly G, Bell D, Patil N, Bowes D, Champion E, Wilke D, Massoeurs L, Hassan N, Rutledge RDH. Assessing the Efficacy of a 28-Day Comprehensive Online Prostate Cancer Patient Empowerment Program (PC-PEP) in Facilitating Engagement of Prostate Cancer Patients in Their Survivorship Care: A Qualitative Study. Curr Oncol. 2023 Sep 21;30(9):8633-8652. doi: 10.3390/curroncol30090626. PMID: 37754541; PMCID: PMC10528560.

Abstract

A 28-day Prostate Cancer-Patient Empowerment Program (PC-PEP) developed through patient engagement was successful at promoting mental and physical health. Thirty prostate cancer patients from Halifax, Canada participated in the 28-day PC-PEP intervention in early 2019. PC-PEP encompassed daily patient education and empowerment videos, prescribed physical activities (including pelvic floor exercises), a mostly plant-based diet, stress reduction techniques, intimacy education, social connection, and support. Quantitative exit surveys and semi-structured interviews (conducted in focus groups of ten) were used to assess perceived factors that facilitated or impeded adherence to the program. The program received high praise from the patients and was deemed extremely useful by the participating men, who rated it 9 out of 10. Patients expressed that the multifaceted, online, home-based nature of the program helped them adhere to it better than they would have had to a single or less comprehensive intervention. Feedback from the participants indicated that the program, when viewed as a whole, was perceived as greater than the sum of its individual parts. Furthermore, the program addressed various issues, including emotional vulnerability and distress, physical fitness, urinary incontinence, challenges in expressing emotions, perceived lack of control over healthcare decisions, emotional fragility, and hesitancy to discuss prostate cancer-related matters in social settings. Patients highly (9.6/10) endorsed integrating the program into the standard care regimen from the very beginning of diagnosis. However, challenges such as work commitments were noted. Patients' high endorsement of PC-PEP suggests that its implementation into the standard of care from day one of diagnosis may be warranted.

Keywords:

prostate cancer; survivorship; patient education; patient advocacy; patient empowerment; patient engagement; patient activation; cancer survivorship; quality of life; mental health

Lay Summary (simple Prompt)

This study looked at a 28-day online program called PC-PEP, designed to help prostate cancer patients manage their health after diagnosis. Thirty men from Halifax, Canada took part in early 2019. The program included daily videos, physical exercises, a mostly plant-based diet, and tips on reducing stress, improving relationships, and connecting with others. Patients rated the program very highly, giving it 9 out of 10. They felt it was more helpful than other single-focus programs because it covered many different areas of health. The program also helped with emotional challenges, such as feeling vulnerable or stressed, and improved physical issues like urinary control. Patients said they would recommend starting the program right after diagnosis and gave it an even higher score (9.6 out of 10) for how useful it was. However, some participants mentioned difficulties balancing the program with work.

Evaluation of the Lay Summary

**Assessment Category**	**Score**	**Assessment Category**	**Score**	**Assessment Category**	**Score**
Comprehension Score	4	Gunning Fog Score	11.7	Words	145
Accuracy Score	0	SMOG Index	8.9	Complex words	21
Reading Age (years)	16.5	Coleman Liau Index	14.0	Percent of complex words	14.5
Flesch Kincaid Reading Ease	49.9	Automated Readability Index	10.4	Average words per sentence	16.1
Flesch Kincaid Grade Level	10.3	Sentences	9	Average syllables per word	1.7

Lay Summary (extended Prompt)

This study examined the effects of a 28-day online program called PC-PEP, designed to help prostate cancer patients manage their health after diagnosis. Thirty men from Halifax, Canada, participated in early 2019. The program included daily educational videos, simple exercises (such as pelvic floor workouts), stress reduction techniques, a mostly plant-based diet, and advice on improving relationships and social connections. Patients rated the program highly, giving it 9 out of 10. They found it more helpful than other programs because it addressed both physical and emotional needs. Participants said it improved their ability to cope with issues like stress, feeling vulnerable, urinary problems, and the difficulty of discussing their cancer. Many strongly recommended starting the program immediately after diagnosis, giving it an even higher rating of 9.6 out of 10 for its overall benefit. However, some mentioned that balancing the program with work was challenging.

Evaluation of the Lay Summary

**Assessment Category**	**Score**	**Assessment Category**	**Score**	**Assessment Category**	**Score**
Comprehension Score	5	Gunning Fog Score	13.9	Words	149
Accuracy Score	0	SMOG Index	10.4	Complex words	29
Reading Age (years)	18.5	Coleman Liau Index	15.6	Percent of complex words	19.5
Flesch Kincaid Reading Ease	39.0	Automated Readability Index	12.0	Average words per sentence	16.6
Flesch Kincaid Grade Level	11.9	Sentences	9	Average syllables per word	1.8

Manuscript 60

Alameddine Z, Niazi MRK, Rajavel A, Behgal J, Keesari PR, Araji G, Mustafa A, Wei C, Jahangir A, Terjanian TO. A Meta-Analysis of Randomized Clinical Trials Assessing the Efficacy of PARP Inhibitors in Metastatic Castration-Resistant Prostate Cancer. Curr Oncol. 2023 Oct 19;30(10):9262-9275. doi: 10.3390/curroncol30100669. PMID: 37887569; PMCID: PMC10605202.

Abstract

Prostate cancer ranks as the second most common malignancy in males. Prostate cancer progressing on androgen deprivation therapy (ADT) is castration-resistant prostate cancer (CRPC). Poly-ADP ribose polymerase (PARP) inhibitors (PARPis) have been at the forefront of the treatment of CRPC. We aim to better characterize the progression-free survival (PFS) and overall survival (OS) in metastatic CRPC patients treated with PARPis. A systemic review search was conducted using National Clinical Trial (NCT), PubMed, Embase, Scopus, and Central Cochrane Registry. The improvement in overall survival was statistically significant, favoring PARPis (hazard ratio (HR) 0.855; 95% confidence interval (CI) 0.752–0.974; p = 0.018). The improvement in progression-free survival was also statistically significant, with results favoring PARPis (HR 0.626; 95%CI 0.566–0.692; p = 0.000). In a subgroup analysis, similar results were observed where the efficacy of PARPis was evaluated in a subgroup of patients without homologous recombination repair (HRR) gene mutation, which showed improvement in PFS favoring PARPis (HR 0.747; 95%CI 0.0.637–0.877; p = 0.000). Our meta-analysis of seven RCTs showed that PARPis significantly increased PFS and OS when used with or without antihormonal agents like abiraterone or enzalutamide.

Keywords:

castrate-resistant prostate cancer; PARP inhibitors; progression-free survival; overall survival; homologous recombination repair genes

Lay Summary (simple Prompt)

This study analyzes the effectiveness of a type of drug called PARP inhibitors in treating advanced prostate cancer that no longer responds to standard hormone therapy. The researchers reviewed data from several clinical trials to determine how well these drugs improve patient outcomes, specifically focusing on how long patients live without their cancer getting worse (progression-free survival) and overall survival. The findings show that PARP inhibitors significantly improve both progression-free survival and overall survival in men with metastatic castration-resistant prostate cancer, even in those without specific genetic mutations. These results suggest that PARP inhibitors, alone or combined with other treatments, could be a valuable option for extending the lives of patients with advanced prostate cancer. This study highlights the potential benefits of PARP inhibitors and supports their use as an important treatment strategy for this challenging condition.

Evaluation of the Lay Summary

**Assessment Category**	**Score**	**Assessment Category**	**Score**	**Assessment Category**	**Score**
Comprehension Score	5	Gunning Fog Score	21.5	Words	140
Accuracy Score	1	SMOG Index	15.4	Complex words	36
Reading Age (years)	23.0	Coleman Liau Index	19.0	Percent of complex words	25.7
Flesch Kincaid Reading Ease	20.7	Automated Readability Index	20.4	Average words per sentence	28.0
Flesch Kincaid Grade Level	17.3	Sentences	5	Average syllables per word	1.9

Lay Summary (extended Prompt)

This study looks at how effective a type of medicine called PARP inhibitors are for treating a serious form of prostate cancer that continues to grow even after standard hormone treatments. Researchers reviewed multiple clinical trials to see if these drugs help patients live longer and prevent the cancer from getting worse. The findings showed that PARP inhibitors significantly improved both overall survival and the time it took for the cancer to progress, even in patients without certain gene mutations. This suggests that PARP inhibitors could be a valuable treatment option for many men with advanced prostate cancer, providing new hope for better management of this challenging disease.

Evaluation of the Lay Summary

**Assessment Category**	**Score**	**Assessment Category**	**Score**	**Assessment Category**	**Score**
Comprehension Score	5	Gunning Fog Score	17.5	Words	108
Accuracy Score	1	SMOG Index	12.3	Complex words	18
Reading Age (years)	21.5	Coleman Liau Index	16.0	Percent of complex words	16.7
Flesch Kincaid Reading Ease	36.1	Automated Readability Index	17.5	Average words per sentence	27.0
Flesch Kincaid Grade Level	14.9	Sentences	4	Average syllables per word	1.7

Manuscript 61

Thomas PB, Alinezhad S, Joshi A, Sweeney K, Tse BWC, Tevz G, McPherson S, Nelson CC, Williams ED, Vela I. Introduction of Androgen Receptor Targeting shRNA Inhibits Tumor Growth in Patient-Derived Prostate Cancer Xenografts. Curr Oncol. 2023 Oct 24;30(11):9437-9447. doi: 10.3390/curroncol30110683. PMID: 37999103; PMCID: PMC10670201.

Abstract

Patient-derived xenograft (PDX) models have been established as important preclinical cancer models, overcoming some of the limitations associated with the use of cancer cell lines. The utility of prostate cancer PDX models has been limited by an inability to genetically manipulate them in vivo and difficulties sustaining PDX-derived cancer cells in culture. Viable, short-term propagation of PDX models would allow in vitro transfection with traceable reporters or manipulation of gene expression relevant to different studies within the prostate cancer field. Here, we report an organoid culture system that supports the growth of prostate cancer PDX cells in vitro and permits genetic manipulation, substantially increasing the scope to use PDXs to study the pathobiology of prostate cancer and define potential therapeutic targets. We have established a short-term PDX-derived in vitro cell culture system which enables genetic manipulation of prostate cancer PDXs LuCaP35 and BM18. Genetically manipulated cells could be re-established as viable xenografts when re-implanted subcutaneously in immunocompromised mice and were able to be serially passaged. Tumor growth of the androgen-dependent LuCaP35 PDX was significantly inhibited following depletion of the androgen receptor (AR) in vivo. Taken together, this system provides a method to generate novel preclinical models to assess the impact of controlled genetic perturbations and allows for targeting specific genes of interest in the complex biological setting of solid tumors.

Keywords:

prostate cancer; organoids; precision medicine; patient-derived xenograft; androgen receptor

Lay Summary (simple Prompt)

This study investigates a new method to improve research on prostate cancer by using patient-derived tumor models, known as xenografts, which are grown in mice. Researchers developed a way to grow these cancer cells in a lab and modify their genes to study specific genetic changes. By targeting the androgen receptor—a key protein that helps prostate cancer grow—with a special genetic tool called shRNA, they were able to significantly slow down tumor growth in these models. This approach allows scientists to better understand the role of specific genes in prostate cancer and could lead to the development of more targeted therapies. The findings suggest that this method could be a powerful tool for studying prostate cancer biology and testing new treatments, potentially leading to more effective ways to combat this disease.

Evaluation of the Lay Summary

**Assessment Category**	**Score**	**Assessment Category**	**Score**	**Assessment Category**	**Score**
Comprehension Score	5	Gunning Fog Score	17.2	Words	132
Accuracy Score	0	SMOG Index	12.1	Complex words	22
Reading Age (years)	20.5	Coleman Liau Index	15.0	Percent of complex words	16.7
Flesch Kincaid Reading Ease	39.0	Automated Readability Index	16.4	Average words per sentence	26.4
Flesch Kincaid Grade Level	14.4	Sentences	5	Average syllables per word	1.7

Lay Summary (extended Prompt)

This study aimed to find better ways to study prostate cancer and develop new treatments. Researchers used patient-derived models of prostate cancer to grow cancer cells in the lab. They created a new method that allows them to manipulate these cells more easily, such as turning off specific genes to see how this affects tumor growth. By targeting the androgen receptor, a key driver in many prostate cancers, they found that tumor growth was significantly slowed down in mice. This approach could help scientists understand how different genes affect prostate cancer and lead to more personalized treatments for patients in the future. The findings show promise for developing new ways to test potential therapies and improve cancer treatment.

Evaluation of the Lay Summary

Assessment Category	Score	Assessment Category	Score	Assessment Category	Score
Comprehension Score	5	Gunning Fog Score	13.0	Words	119
Accuracy Score	1	SMOG Index	9.2	Complex words	15
Reading Age (years)	17.5	Coleman Liau Index	14.8	Percent of complex words	12.6
Flesch Kincaid Reading Ease	51.6	Automated Readability Index	13.0	Average words per sentence	19.8
Flesch Kincaid Grade Level	11.0	Sentences	6	Average syllables per word	1.6

Manuscript 62

Kato M, Higashi S, Sugino Y, Kajiwara S, Tanaka S, Kitano G, Yamashita Y, Ogura Y, Tachibana H, Kojima T, Inoue T. Clinical Efficacy and Openness to New Challenges of Low Dose Rate Brachytherapy for Prostate Cancer. Curr Oncol. 2023 Nov 8;30(11):9824-9835. doi: 10.3390/curroncol30110713. PMID: 37999133; PMCID: PMC10670683.

Abstract

Over a century ago, low-dose-rate (LDR) brachytherapy was introduced to treat prostate cancer (PCa). Since then, it has been widely applied worldwide, including in East Asia. LDR brachytherapy has been performed in 88 institutes in Japan. Beneficial clinical outcomes of LDR brachytherapy for intermediate-to-high-risk PCa have been demonstrated in large clinical trials. These clinical outcomes were achieved through advances in methods, such as urological precise needle puncture and seed placement, and the quantitative decision making regarding radiological parameters by radiation oncologists. The combined use of LDR brachytherapy with other therapeutic modalities, such as external beam radiation and androgen deprivation therapy, for the clinical risk classification of PCa has led to better anticancer treatment efficacy. In this study, we summarized basic LDR brachytherapy findings that should remain unchanged and be passed down in urology departments. We also discussed the applications of LDR brachytherapy for PCa in various clinical settings, including focal and salvage therapies. In addition, we highlighted technologies associated with brachytherapy that are under development.

Keywords:

prostate cancer; brachytherapy; low-dose-rate brachytherapy; tri-modality therapy; salvage brachytherapy; focal brachytherapy

Lay Summary (simple Prompt)

This study reviews the effectiveness and evolving use of low-dose-rate (LDR) brachytherapy, a type of radiation treatment for prostate cancer. LDR brachytherapy has been used for over a century and remains a common treatment, especially for patients with intermediate to high-risk prostate cancer. The study highlights improvements in techniques, such as precise placement of radiation seeds, which have enhanced treatment outcomes. Combining LDR brachytherapy with other therapies, like external radiation and hormone therapy, has shown even better results. The research also explores new applications of LDR brachytherapy in different clinical settings, such as focused treatment for specific tumor areas and salvage therapy for recurrent cancer. The findings suggest that LDR brachytherapy continues to be a valuable treatment option and could benefit from ongoing technological advancements, potentially improving outcomes for more prostate cancer patients.

Evaluation of the Lay Summary

**Assessment Category**	**Score**	**Assessment Category**	**Score**	**Assessment Category**	**Score**
Comprehension Score	5	Gunning Fog Score	19.1	Words	136
Accuracy Score	1	SMOG Index	13.7	Complex words	34
Reading Age (years)	22.5	Coleman Liau Index	19.3	Percent of complex words	25.0
Flesch Kincaid Reading Ease	19.0	Automated Readability Index	18.0	Average words per sentence	22.7
Flesch Kincaid Grade Level	16.2	Sentences	6	Average syllables per word	2.0

Lay Summary (extended Prompt)

This study looks at the use of low-dose-rate (LDR) brachytherapy, a type of radiation treatment, for prostate cancer. LDR brachytherapy involves placing tiny radioactive seeds directly into the prostate to target cancer cells. This method has been used for over a century and has shown good results, especially for patients with intermediate to high-risk prostate cancer. Recent improvements, such as more precise placement of the seeds and better use of additional treatments like external radiation and hormone therapy, have made this approach even more effective. The study also explores new uses for LDR brachytherapy, such as treating specific areas of the prostate or as a follow-up treatment when other therapies don't work. These findings help guide future research and improve the treatment of prostate cancer.

Evaluation of the Lay Summary

**Assessment Category**	**Score**	**Assessment Category**	**Score**	**Assessment Category**	**Score**
Comprehension Score	5	Gunning Fog Score	14.5	Words	129
Accuracy Score	0	SMOG Index	10.3	Complex words	19
Reading Age (years)	18.5	Coleman Liau Index	15.1	Percent of complex words	14.7
Flesch Kincaid Reading Ease	42.0	Automated Readability Index	14.0	Average words per sentence	21.5
Flesch Kincaid Grade Level	12.7	Sentences	6	Average syllables per word	1.7

Manuscript 63

Urquhart R, Scruton S, Radford S, Kendell C, Hirsch E. Exploring Men's Experiences with Follow-Up Care following Primary Treatment for Prostate Cancer in Atlantic Canada: A Qualitative Study. Curr Oncol. 2023 Nov 25;30(12):10111-10123. doi: 10.3390/curroncol30120735. PMID: 38132369; PMCID: PMC10742932.

Abstract

Prostate cancer is a common and life-altering condition among Canadian men, yet little is known about how follow-up care is provided to those who have completed treatment. Despite improving survival rates, survivors experience ongoing needs and are often not provided with support to manage them. This study sought to investigate the post-treatment experiences and needs of prostate cancer survivors and to determine if and how these needs are being met. Using a qualitative description design, prostate cancer survivors who had completed treatment took part in semi-structured interviews. The interviews were recorded and analyzed thematically. The participants experienced varying levels of satisfaction with their follow-up care. While primary care providers played significant roles, continuity of care and specialist involvement varied. Most participants felt unprepared to manage the long-term effects of their cancer due to a lack of information and resources from their healthcare providers. Instead, participants turned to their peers for support. Ongoing physical and psychosocial needs went unmet and had significant impacts on their daily lives. Participants felt that support for these issues should be automatically integrated into their follow-up care. In summary, this study revealed the importance of integrated, patient-centered follow-up care for prostate cancer in Atlantic Canada.

Keywords:

follow-up care; prostate cancer; survivorship; qualitative methods

Lay Summary (simple Prompt)

This study explores the experiences of men in Atlantic Canada who have completed primary treatment for prostate cancer, focusing on their follow-up care. Through interviews with prostate cancer survivors, the researchers found that many men felt unprepared to handle the long-term effects of their cancer due to a lack of information and support from healthcare providers. While some received good follow-up care from their primary doctors, many reported gaps in care, especially regarding specialist involvement and continuity of care. As a result, many survivors turned to peers for support and guidance. The study highlights that ongoing physical and emotional needs are often unmet and significantly impact survivors' daily lives. The findings suggest that follow-up care for prostate cancer should be more integrated and patient-centered, with better resources and support automatically provided to help survivors manage their health and well-being after treatment.

Evaluation of the Lay Summary

**Assessment Category**	**Score**	**Assessment Category**	**Score**	**Assessment Category**	**Score**
Comprehension Score	4	Gunning Fog Score	17.1	Words	147
Accuracy Score	0	SMOG Index	12.7	Complex words	29
Reading Age (years)	21.5	Coleman Liau Index	16.7	Percent of complex words	19.7
Flesch Kincaid Reading Ease	32.9	Automated Readability Index	16.8	Average words per sentence	24.5
Flesch Kincaid Grade Level	14.8	Sentences	6	Average syllables per word	1.8

Lay Summary (extended Prompt)

This study looked at the experiences of men in Atlantic Canada who have finished their treatment for prostate cancer. Many of these men felt unprepared to handle the long-term effects of their cancer because they did not receive enough information or resources from their healthcare providers. The study found that while some patients were happy with their follow-up care, others felt their ongoing physical and emotional needs were not being met. Many men had to rely on friends and other patients for support instead of their doctors. The research highlights the need for better follow-up care that includes more support and information to help prostate cancer survivors manage their health and well-being after treatment. This kind of care could improve their quality of life by addressing both their physical and emotional needs.

Evaluation of the Lay Summary

**Assessment Category**	**Score**	**Assessment Category**	**Score**	**Assessment Category**	**Score**
Comprehension Score	5	Gunning Fog Score	12.9	Words	136
Accuracy Score	0	SMOG Index	9.2	Complex words	15
Reading Age (years)	17.5	Coleman Liau Index	14.1	Percent of complex words	11.0
Flesch Kincaid Reading Ease	54.4	Automated Readability Index	13.8	Average words per sentence	22.7
Flesch Kincaid Grade Level	11.3	Sentences	6	Average syllables per word	1.5

Manuscript 64

Vermeille M, Koster KL, Benzaquen D, Champion A, Taussky D, Kaulanjan K, Früh M. A Literature Review of Racial Disparities in Prostate Cancer Research. Curr Oncol. 2023 Nov 12;30(11):9886-9894. doi: 10.3390/curroncol30110718. PMID: 37999138; PMCID: PMC10670533.

Abstract

Background: Despite recent awareness of institutional racism, there are still important racial disparities in prostate cancer medical research. We investigated the historical development of research on racial disparities and bias. Methods: PubMed was searched for the term 'prostate cancer race' and added key terms associated with racial disparity. As an indicator of scientific interest in the topic, we analyzed whether the number of publications increased linearly as an indicator of growing interest. The linearity is expressed as R2. Results: The general search term "prostate cancer race" yielded 4507 publications. More specific search terms with ≥12 publications showing a higher scientific interest were found after 2005. The terms with the most publications when added to the general term were "genetic" (n = 1011), "PSA" (n = 995), and "detection" (n = 861). There was a linear increase in publications for "prostate cancer race" (R2 = 0.75) since 1980. Specific terms added to the general terms with a high linear increase (R2 ≥ 0.7) were "screening" (R2 = 0.82), "detection" (R2 = 0.72), "treatment access" (R2 = 0.71), and "trial underrepresentation" (R2 = 0.71). However, only a few studies have investigated its association with sexual activity. A combination with "sexual" showed 157 publications but only two years with ≥12 publications/year. Conclusion: The terms "genetic", "PSA", and "detection" have been the focus of recent research on racial differences in prostate cancer. We found that old stereotypes are still being mentioned but seem to find little interest in the current literature. Further research interest was found in "treatment access". Recently, interest in socioeconomic factors has decreased.

Keywords:

prostate cancer; racial disparity; detection; treatment access; socioeconomic factors

Lay Summary (simple Prompt)

This study reviews the current state of research on racial disparities in prostate cancer, highlighting areas that have received the most scientific attention. By analyzing publications on topics such as genetics, PSA (prostate-specific antigen) testing, and detection, the researchers found that interest in these areas has increased significantly over time. However, topics like treatment access and trial underrepresentation are still under-explored, and there is limited research on how prostate cancer is associated with sexual activity among different racial groups. The study suggests that while there has been progress in understanding some aspects of racial disparities in prostate cancer, more research is needed to address gaps, particularly in access to care and socioeconomic factors. This work emphasizes the importance of continuing to investigate racial disparities to improve healthcare equity and outcomes for all patients with prostate cancer.

Evaluation of the Lay Summary

Assessment Category	Score	Assessment Category	Score	Assessment Category	Score
Comprehension Score	5	Gunning Fog Score	19.7	Words	138
Accuracy Score	1	SMOG Index	14.1	Complex words	30
Reading Age (years)	23.5	Coleman Liau Index	18.8	Percent of complex words	21.7
Flesch Kincaid Reading Ease	15.8	Automated Readability Index	20.0	Average words per sentence	27.6
Flesch Kincaid Grade Level	17.9	Sentences	5	Average syllables per word	1.9

Lay Summary (extended Prompt)

This study reviews research on racial differences in prostate cancer to see how interest in this topic has changed over time. The researchers looked at thousands of studies to see which areas have been most focused on. They found that studies on genetic differences, PSA testing, and cancer detection are the most common. There has been an increase in research on racial disparities in screening, access to treatment, and the underrepresentation of certain groups in clinical trials. However, there has been less focus on how socioeconomic factors affect these disparities. This study highlights the need for more research into the barriers that different racial groups face in accessing prostate cancer care and understanding how these barriers can be reduced.

Evaluation of the Lay Summary

**Assessment Category**	**Score**	**Assessment Category**	**Score**	**Assessment Category**	**Score**
Comprehension Score	5	Gunning Fog Score	13.6	Words	119
Accuracy Score	1	SMOG Index	9.8	Complex words	17
Reading Age (years)	18.5	Coleman Liau Index	15.5	Percent of complex words	14.3
Flesch Kincaid Reading Ease	45.9	Automated Readability Index	13.5	Average words per sentence	19.8
Flesch Kincaid Grade Level	11.8	Sentences	6	Average syllables per word	1.7

Manuscript 65

Yang J, Xiong X, Zheng W, Liao X, Xu H, Yang L, Wei Q. Combining Novel Hormonal Therapies with a Poly (ADP-Ribose) Polymerase Inhibitor for Metastatic Castration-Resistant Prostate Cancer: Emerging Evidence. Curr Oncol. 2023 Dec 4;30(12):10311-10324. doi: 10.3390/curroncol30120751. PMID: 38132385; PMCID: PMC10742907.

Abstract

Preclinical and clinical studies have suggested potential synergies of combining poly (ADP-ribose) polymerase (PARP) inhibitors and novel hormonal therapies (NHT) for patients with metastatic castration-resistant prostate cancer (mCRPC). We systematically searched PubMed, ClinicalTrials.gov and ASCO-GU annual meeting abstracts up to March 2023 to identify potential phase III trials reporting the use of combining PARP inhibitors with NHT in the first-line setting for mCRPC. A total of four phase III trials met the criteria for subsequent review. Emerging data suggested that the radiographic progression-free survival (rPFS) was significantly longer in the PARP inhibitor combined with NHT group versus the placebo plus NHT group for the first-line setting of biomarker-unselected mCRPC patients, especially for patients with homologous recombination repair (HRR) mutation (HRR m), and with the greatest benefit for BRCA1/2 mutation (BRCA1/2 m) populations. Final overall survival (OS) data of the PROpel trial indicated a significant improvement in median OS for mCRPC patients with HRR m and BRCA1/2 m receiving olaparib + abiraterone. Prior taxane-based chemotherapy might not influence the efficacy of the combination. Compared with the current standard-of-care therapies, combining NHT with PARP inhibitors could achieve a significant survival benefit in the first-line setting for mCRPC patients with HRR and BRCA1/2 mutations.

Keywords:

poly (ADP-ribose) polymerase inhibitor; mCRPC; first-line therapy; homologous recombination repair

Lay Summary (simple Prompt)

This study reviews the potential benefits of combining a type of targeted cancer drug, known as PARP inhibitors, with novel hormonal therapies for treating metastatic castration-resistant prostate cancer, a form of cancer that has spread and no longer responds to traditional hormone treatments. The researchers analyzed data from four major clinical trials and found that this combination therapy significantly extended the time patients lived without their cancer progressing, particularly in those with specific genetic mutations, such as BRCA1/2. The results also showed that this combined treatment approach could improve overall survival compared to standard therapies. These findings suggest that using PARP inhibitors together with hormonal therapies could offer a new and more effective treatment option for patients with advanced prostate cancer, especially those with certain genetic profiles, potentially leading to better outcomes and longer survival.

Evaluation of the Lay Summary

**Assessment Category**	**Score**	**Assessment Category**	**Score**	**Assessment Category**	**Score**
Comprehension Score	5	Gunning Fog Score	23.6	Words	136
Accuracy Score	1	SMOG Index	16.8	Complex words	34
Reading Age (years)	23.0	Coleman Liau Index	19.5	Percent of complex words	25.0
Flesch Kincaid Reading Ease	12.5	Automated Readability Index	23.8	Average words per sentence	34.0
Flesch Kincaid Grade Level	20.0	Sentences	4	Average syllables per word	1.9

Lay Summary (extended Prompt)

This study explores the benefits of combining two types of treatments, PARP inhibitors and new hormonal therapies, for patients with metastatic castration-resistant prostate cancer (mCRPC). Researchers reviewed four clinical trials to see how well these combined treatments work compared to current therapies. They found that using both PARP inhibitors and hormonal therapies together helped patients live longer without their cancer getting worse, especially those with specific genetic mutations like BRCA1/2. These findings suggest that this combination could be more effective than the current standard treatments for certain groups of patients with advanced prostate cancer, offering a promising new approach to improve survival and outcomes.

Evaluation of the Lay Summary

**Assessment Category**	**Score**	**Assessment Category**	**Score**	**Assessment Category**	**Score**
Comprehension Score	5	Gunning Fog Score	19.6	Words	105
Accuracy Score	1	SMOG Index	14.1	Complex words	24
Reading Age (years)	23.5	Coleman Liau Index	19.5	Percent of complex words	22.9
Flesch Kincaid Reading Ease	28.7	Automated Readability Index	20.0	Average words per sentence	26.3
Flesch Kincaid Grade Level	15.8	Sentences	4	Average syllables per word	1.8

Manuscript 66

Choi MH, Lee YJ, Han D, Kim DH. Quantitative Analysis of Prostate MRI: Correlation between Contrast-Enhanced Magnetic Resonance Fingerprinting and Dynamic Contrast-Enhanced MRI Parameters. Curr Oncol. 2023 Dec 4;30(12):10299-10310. doi: 10.3390/curroncol30120750. PMID: 38132384; PMCID: PMC10743035.

Abstract

This research aimed to assess the relationship between contrast-enhanced (CE) magnetic resonance fingerprinting (MRF) values and dynamic contrast-enhanced (DCE) MRI parameters including (Ktrans, Kep, Ve, and iAUC). To evaluate the correlation between the MRF-derived values (T1 and T2 values, CE T1 and T2 values, T1 and T2 change) and DCE-MRI parameters and the differences in the parameters between prostate cancer and noncancer lesions in 68 patients, two radiologists independently drew regions-of-interest (ROIs) at the focal prostate lesions. Prostate cancer was identified in 75% (51/68) of patients. The CE T2 value was significantly lower in prostate cancer than in noncancer lesions in the peripheral zone and transition zone. Ktrans, Kep, and iAUC were significantly higher in prostate cancer than noncancer lesions in the peripheral zone ($p < 0.05$), but not in the transition zone. The CE T1 value was significantly correlated with Ktrans, Ve, and iAUC in prostate cancer, and the CE T2 value was correlated to Ve in noncancer. Some CE MRF values are different between prostate cancer and noncancer tissues and correlate with DCE-MRI parameters. Prostate cancer and noncancer tissues may have different characteristics regarding contrast enhancement.

Keywords:

magnetic resonance fingerprinting; dynamic contrast-enhanced magnetic resonance imaging; quantitative analysis; prostate; prostate neoplasm

Lay Summary (simple Prompt)

This study explores the use of two advanced MRI techniques to better differentiate between cancerous and noncancerous tissues in the prostate. The researchers compared contrast-enhanced magnetic resonance fingerprinting (MRF) with dynamic contrast-enhanced MRI (DCE-MRI) to see how their measurements relate and whether they can more accurately identify prostate cancer. By analyzing MRI scans from 68 patients, they found that certain MRI values were significantly different between cancerous and noncancerous tissues, particularly in specific areas of the prostate. They also discovered that some measurements from the two MRI techniques were correlated, indicating they might provide complementary information. These findings suggest that combining these imaging methods could improve the accuracy of prostate cancer diagnosis, helping doctors better distinguish between cancerous and healthy tissue. This research could lead to more precise and effective ways to diagnose and manage prostate cancer.

Evaluation of the Lay Summary

**Assessment Category**	**Score**	**Assessment Category**	**Score**	**Assessment Category**	**Score**
Comprehension Score	5	Gunning Fog Score	19.0	Words	140
Accuracy Score	1	SMOG Index	13.7	Complex words	34
Reading Age (years)	22.5	Coleman Liau Index	19.9	Percent of complex words	24.3
Flesch Kincaid Reading Ease	23.6	Automated Readability Index	18.8	Average words per sentence	23.3
Flesch Kincaid Grade Level	15.8	Sentences	6	Average syllables per word	1.9

Lay Summary (extended Prompt)

This study looked at how different types of MRI scans can help distinguish between cancerous and noncancerous tissue in the prostate. Researchers used two advanced imaging techniques, contrast-enhanced magnetic resonance fingerprinting (MRF) and dynamic contrast-enhanced (DCE) MRI, to measure various properties of the prostate tissue in 68 patients. They found that certain values from these scans, like CE T2, were lower in cancerous tissue compared to noncancerous tissue. Other measurements, like Ktrans and iAUC, were higher in cancerous areas. These findings suggest that these imaging techniques could be useful tools in identifying prostate cancer more accurately, which could help doctors make better treatment decisions and improve patient outcomes.

Evaluation of the Lay Summary

**Assessment Category**	**Score**	**Assessment Category**	**Score**	**Assessment Category**	**Score**
Comprehension Score	5	Gunning Fog Score	17.2	Words	110
Accuracy Score	1	SMOG Index	12.4	Complex words	23
Reading Age (years)	21.5	Coleman Liau Index	18.0	Percent of complex words	20.9
Flesch Kincaid Reading Ease	36.1	Automated Readability Index	16.6	Average words per sentence	22.0
Flesch Kincaid Grade Level	13.7	Sentences	5	Average syllables per word	1.8

Manuscript 67

Batra A, Glick D, Valdes M. Contemporary Systemic Therapy Intensification for Prostate Cancer: A Review for General Practitioners in Oncology. Curr Oncol. 2024 Feb 15;31(2):1047-1062. doi: 10.3390/curroncol31020078. PMID: 38392072; PMCID: PMC10888029.

Abstract

Prostate cancer accounts for a significant proportion of cancer diagnoses in Canadian men. Over the past decade, the therapeutic landscape for the management of metastatic prostate cancer has undergone rapid changes. Novel strategies use hormonal agents, chemotherapy, homologous recombination repair inhibitors, and radioligand therapy or combination strategies in addition to androgen deprivation therapy. In this review, we summarize the available data addressing key therapeutic areas along the disease continuum and focus on practical aspects for general practitioners in oncology managing patients with metastatic prostate cancer.

Keywords:

prostate cancer; ARAT; androgen receptor axis-targeted drugs; docetaxel; BRCA1/2; breast cancer gene 1/2; PARP inhibitors; poly ADP ribose polymerase inhibitors; prostate-specific membrane antigen; PSMA; CAGPO; Canadian Association of General Practitioners in Oncology

Lay Summary (simple Prompt)

This study reviews the latest advancements in treatment options for metastatic prostate cancer, which is cancer that has spread beyond the prostate. Over the past decade, new therapies have emerged, including advanced hormonal treatments, chemotherapy, targeted drugs for specific genetic mutations, and radioligand therapy. The review focuses on providing general practitioners in oncology with practical guidance on using these treatments in combination with traditional androgen deprivation therapy to improve patient outcomes. By summarizing the current evidence and highlighting key therapeutic strategies, the study aims to help doctors better manage patients with advanced prostate cancer, offering more personalized and effective treatment plans. This research is important for the medical community as it provides up-to-date knowledge on managing prostate cancer and can lead to better patient care and improved survival rates.

Evaluation of the Lay Summary

**Assessment Category**	**Score**	**Assessment Category**	**Score**	**Assessment Category**	**Score**
Comprehension Score	5	Gunning Fog Score	21.2	Words	131
Accuracy Score	1	SMOG Index	15.2	Complex words	35
Reading Age (years)	23.0	Coleman Liau Index	19.8	Percent of complex words	26.7
Flesch Kincaid Reading Ease	17.5	Automated Readability Index	20.2	Average words per sentence	26.2
Flesch Kincaid Grade Level	17.3	Sentences	5	Average syllables per word	1.9

Lay Summary (extended Prompt)

This study reviews the latest advancements in treatments for metastatic prostate cancer, a common cancer in Canadian men. Over the past decade, new treatments, including hormonal therapies, chemotherapy, targeted drugs, and radioligand therapy, have been developed to improve patient outcomes. The study aims to provide general oncologists with practical guidance on how to use these new therapies effectively. By summarizing current research and treatment strategies, the review helps doctors better understand how to manage prostate cancer using a range of therapies, depending on each patient's specific needs. This knowledge is important for improving the quality of care and survival rates for patients with advanced prostate cancer.

Evaluation of the Lay Summary

**Assessment Category**	**Score**	**Assessment Category**	**Score**	**Assessment Category**	**Score**
Comprehension Score	5	Gunning Fog Score	17.9	Words	106
Accuracy Score	1	SMOG Index	13.2	Complex words	26
Reading Age (years)	21.5	Coleman Liau Index	18.5	Percent of complex words	24.5
Flesch Kincaid Reading Ease	27.3	Automated Readability Index	16.6	Average words per sentence	21.2
Flesch Kincaid Grade Level	14.7	Sentences	5	Average syllables per word	1.9

Manuscript 68

De Bari B, Guibert G, Slimani S, Bashar Y, Risse T, Guisolan N, Trouillot J, Abel J, Weber P. Electromagnetic Transmitter-Based Prostate Gating for Dose-Escalated Linac-Based Stereotactic Body Radiation Therapy: An Evaluation of Intrafraction Motion. Curr Oncol. 2024 Feb 9;31(2):962-974. doi: 10.3390/curroncol31020072. PMID: 38392066; PMCID: PMC10887766.

Abstract

Background: Stereotactic Body Radiotherapy (SBRT) is as a standard treatment for prostate cancer (PCa). Tight margins and high dose gradients are needed, and the precise localization of the target is mandatory. Our retrospective study reports our experience regarding the evaluation of intrafraction prostate motion during LINAC-based SBRT evaluated with a novel electromagnetic (EM) tracking device. This device consists of an integrated Foley catheter with a transmitter connected to a receiver placed on the treatment table. Methods: We analyzed 31 patients who received LINAC-based SBRT using flattening filter-free (FFF) volumetric modulated arc therapy (VMAT). The patients were scheduled to be treated for primary (n = 27) or an intraprostatic recurrent PCa (n = 4). A simulation CT scan was conducted while the patients had a filled bladder (100–150 cc) and an empty rectum, and an EM tracking device was used. The same rectal and bladder conditions were employed during the treatment. The patients received 36.25 Gy delivered over five consecutive fractions on the whole prostate and 40 Gy on the nodule(s) visible via MRI, both delivered with a Simultaneous Integrated Boost approach. The CTV-to-PTV margin was 2 mm for both the identified treatment volumes. Patient positioning was verified with XVI ConeBeam-CT (CBCT) matching before each fraction. When the signals exceeded a 2 mm threshold in any of the three spatial directions, the treatment was manually interrupted. A new XVI CBCT was performed if this offset lasted >20 s. Results: We analyzed data about 155 fractions. The median and mean treatment times, calculated per fraction, were 10 m31 s and 12 m44 s (range: 6 m36 s–65 m28 s), and 95% of the fractions were delivered with a maximum time of 27 m48 s. During treatment delivery, the mean and median number of XVI CBCT operations realized during the treatment were 2 and 1 (range: 0–11). During the treatment, the prostate was outside the CTV-to-PTV margin (2 mm), thus necessitating the stoppage of the delivery +/− a

reacquisition of the XVI CBCT for 11.2%, 8.9%, and 3.9% of the delivery time in the vertical, longitudinal, and lateral direction, respectively. Conclusions: We easily integrated an EM-transmitter-based gating for prostate LINAC-based SBRT into our normal daily workflow. Using this system, a 2 mm CTV-to-PTV margin could be safely applied. A small number of fractions showed a motion exceeding the predefined 2 mm threshold, which would have otherwise gone undetected without intrafraction motion management.

Keywords:

prostate cancer; stereotactic body radiation therapy (SBRT); extreme hypofractionation; image-guided radiation therapy (IGRT); intrafraction motion mitigation; real-time electromagnetic tracking

Lay Summary (simple Prompt)

This study explores a new technique for improving the accuracy of a type of radiation therapy, called Stereotactic Body Radiotherapy (SBRT), used to treat prostate cancer. The researchers used an electromagnetic tracking device to monitor prostate movement during treatment to ensure the radiation targets the cancer precisely. They analyzed data from 31 patients who received SBRT and found that the tracking device effectively detected small movements of the prostate, which allowed for adjustments during therapy. This precision is crucial because even small shifts can affect treatment effectiveness and safety. The study showed that using this tracking technology makes it possible to use tighter margins, meaning less healthy tissue is exposed to radiation, potentially reducing side effects. These findings suggest that real-time tracking could become an important tool in delivering more accurate and effective radiation therapy for prostate cancer patients.

Evaluation of the Lay Summary

**Assessment Category**	**Score**	**Assessment Category**	**Score**	**Assessment Category**	**Score**
Comprehension Score	5	Gunning Fog Score	18.2	Words	140
Accuracy Score	1	SMOG Index	13.5	Complex words	33
Reading Age (years)	22.5	Coleman Liau Index	18.3	Percent of complex words	23.6
Flesch Kincaid Reading Ease	24.2	Automated Readability Index	17.6	Average words per sentence	23.3
Flesch Kincaid Grade Level	15.7	Sentences	6	Average syllables per word	1.9

Lay Summary (extended Prompt)

This study looked at using a new technology to help guide radiation treatment for prostate cancer more accurately. The technology uses a small device placed inside the body to track the prostate's position in real-time during treatment. This is important because the prostate can move slightly, which can affect how the radiation is delivered. By using this tracking device, doctors were able to stop and adjust the treatment whenever the prostate moved more than 2 millimeters from its target position. The study found that using this tracking method made the radiation treatment safer and more precise, potentially improving outcomes for patients with prostate cancer. This technology could help ensure that patients receive the most effective treatment with fewer side effects.

Evaluation of the Lay Summary

**Assessment Category**	**Score**	**Assessment Category**	**Score**	**Assessment Category**	**Score**
Comprehension Score	5	Gunning Fog Score	13.4	Words	121
Accuracy Score	0	SMOG Index	9.5	Complex words	16
Reading Age (years)	18.5	Coleman Liau Index	15.8	Percent of complex words	13.2
Flesch Kincaid Reading Ease	46.5	Automated Readability Index	13.9	Average words per sentence	20.2
Flesch Kincaid Grade Level	11.8	Sentences	6	Average syllables per word	1.7

Manuscript 69

Maggio A, Rancati T, Gatti M, Cante D, Avuzzi B, Bianconi C, Badenchini F, Farina B, Ferrari P, Giandini T, Girelli G, Landoni V, Magli A, Moretti E, Petrucci E, Salmoiraghi P, Sanguineti G, Villa E, Waskiewicz JM, Guarneri A, Valdagni R, Fiorino C, Cozzarini C. Quality of Life Longitudinal Evaluation in Prostate Cancer Patients from Radiotherapy Start to 5 Years after IMRT-IGRT. Curr Oncol. 2024 Feb 1;31(2):839-848. doi: 10.3390/curroncol31020062. PMID: 38392056; PMCID: PMC10887595.

Abstract

Purpose: The purpose of this study is to study the evolution of quality of life (QoL) in the first 5 years following Intensity-modulated radiation therapy (IMRT) for prostate cancer (PCa) and to determine possible associations with clinical/treatment data. Material and methods: Patients were enrolled in a prospective multicentre observational trial in 2010-2014 and treated with conventional (74–80 Gy, 1.8–2 Gy/fr) or moderately hypofractionated IMRT (65–75.2 Gy, 2.2–2.7 Gy/fr). QoL was evaluated by means of EORTC QLQ-C30 at baseline, at radiation therapy (RT) end, and every 6 months up to 5 years after IMRT end. Fourteen QoL dimensions were investigated separately. The longitudinal evaluation of QoL was analysed by means of Analysis of variances (ANOVA) for multiple measures. Results: A total of 391 patients with complete sets of questionnaires across 5 years were available. The longitudinal analysis showed a trend toward the significant worsening of QoL at RT end for global health, physical and role functioning, fatigue, appetite loss, diarrhoea, and pain. QoL worsening was recovered within 6 months from RT end, with the only exception being physical functioning. Based on ANOVA, the most impaired time point was RT end. QoL dimension analysis at this time indicated that acute Grade ≥ 2 gastrointestinal (GI) toxicity significantly impacted global health, physical and role functioning, fatigue, appetite loss, diarrhoea, and pain. Acute Grade ≥ 2 genitourinary (GU) toxicity resulted in lower role functioning and higher pain. Prophylactic lymph-nodal irradiation (WPRT) resulted in significantly lower QoL for global health, fatigue, appetite loss, and diarrhoea; lower pain with the use of neoadjuvant/concomitant hormonal therapy; and lower fatigue with the use of an anti-androgen. Conclusions: In this prospective, longitudinal, observational study, high radiation IMRT doses delivered for PCa led to a temporary worsening of QoL, which

tended to be completely resolved at six months. Such transient worsening was mostly associated with acute GI/GU toxicity, WPRT, and higher prescription doses.

Keywords:
prostate cancer; radiotherapy; EORTCQLQ-C30 questionnaire; quality of life

Lay Summary (simple Prompt)

This study examines how prostate cancer patients' quality of life changes over the first five years after receiving a type of advanced radiation therapy called intensity-modulated radiation therapy (IMRT). Researchers followed 391 patients, tracking their well-being from the start of treatment and at regular intervals afterward. The findings showed that many patients experienced a temporary decline in quality of life immediately following treatment, particularly in terms of overall health, physical ability, fatigue, appetite, and pain. However, most of these issues improved within six months after treatment ended, except for physical functioning, which took longer to recover. The study also found that certain treatment-related factors, like high radiation doses and specific types of therapy, were linked to a greater decline in quality of life. Understanding these effects can help doctors better manage and support prostate cancer patients during and after treatment.

Evaluation of the Lay Summary

**Assessment Category**	**Score**	**Assessment Category**	**Score**	**Assessment Category**	**Score**
Comprehension Score	5	Gunning Fog Score	18.5	Words	143
Accuracy Score	1	SMOG Index	13.3	Complex words	32
Reading Age (years)	21.5	Coleman Liau Index	17.7	Percent of complex words	22.4
Flesch Kincaid Reading Ease	28.2	Automated Readability Index	17.3	Average words per sentence	23.8
Flesch Kincaid Grade Level	15.2	Sentences	6	Average syllables per word	1.8

Lay Summary (extended Prompt)

This study looked at how the quality of life changes for men with prostate cancer in the first five years after receiving a type of radiation therapy called IMRT. Researchers followed 391 patients, measuring their quality of life from the start of treatment, at the end, and every six months for five years. They found that many patients experienced a decline in their overall health, energy, and comfort levels, especially right after finishing treatment. This included issues like fatigue, appetite loss, and pain. However, these problems generally improved within six months, except for physical functioning, which took longer to recover. The study highlights that the temporary drop in quality of life is often linked to side effects from the radiation treatment. This information can help doctors better prepare patients for what to expect and manage any side effects more effectively.

Evaluation of the Lay Summary

Assessment Category	Score	Assessment Category	Score	Assessment Category	Score
Comprehension Score	5	Gunning Fog Score	14.9	Words	140
Accuracy Score	0	SMOG Index	10.7	Complex words	24
Reading Age (years)	18.5	Coleman Liau Index	14.9	Percent of complex words	17.1
Flesch Kincaid Reading Ease	48.2	Automated Readability Index	13.1	Average words per sentence	20.0
Flesch Kincaid Grade Level	11.5	Sentences	7	Average syllables per word	1.6

Manuscript 70

Kalampokis N, Zabaftis C, Spinos T, Karavitakis M, Leotsakos I, Katafigiotis I, van der Poel H, Grivas N, Mitropoulos D. Review on the Role of BRCA Mutations in Genomic Screening and Risk Stratification of Prostate Cancer. Curr Oncol. 2024 Feb 22;31(3):1162-1169. doi: 10.3390/curroncol31030086. PMID: 38534919; PMCID: PMC10969585.

Abstract

(1) Background: Somatic and germline alterations can be commonly found in prostate cancer (PCa) patients. The aim of our present study was to perform a comprehensive review of the current literature in order to examine the impact of BRCA mutations in the context of PCa as well as their significance as genetic biomarkers. (2) Methods: A narrative review of all the available literature was performed. Only "landmark" publications were included. (3) Results: Overall, the number of PCa patients who harbor a BRCA2 mutation range between 1.2% and 3.2%. However, BRCA2 and BRCA1 mutations are responsible for most cases of hereditary PCa, increasing the risk by 3–8.6 times and up to 4 times, respectively. These mutations are correlated with aggressive disease and poor prognosis. Gene testing should be offered to patients with metastatic PCa, those with 2–3 first-degree relatives with PCa, or those aged < 55 and with one close relative with breast (age ≤ 50 years) or invasive ovarian cancer. (4) Conclusions: The individualized assessment of BRCA mutations is an important tool for the risk stratification of PCa patients. It is also a population screening tool which can guide our risk assessment strategies and achieve better results for our patients and their families.

Keywords:

prostate cancer; genomic screening; mutations

Lay Summary (simple Prompt)

This study reviews the role of BRCA gene mutations in understanding and managing prostate cancer. BRCA mutations, particularly in the BRCA1 and BRCA2 genes, are known to increase the risk of developing aggressive prostate cancer and lead to poorer outcomes. The review examines how these genetic mutations can be used as biomarkers to help identify patients who are at a higher risk of developing prostate cancer or experiencing a more severe form of the disease. It suggests that genetic testing for BRCA mutations should be offered to certain groups, such as those with a family history of prostate or breast cancer. Understanding the presence of these mutations can help doctors better assess a patient's risk and personalize their treatment, ultimately improving outcomes for patients and their families. This research emphasizes the importance of genetic screening in guiding prostate cancer diagnosis and treatment strategies.

Evaluation of the Lay Summary

Assessment Category	Score	Assessment Category	Score	Assessment Category	Score
Comprehension Score	4	Gunning Fog Score	17.6	Words	143
Accuracy Score	0	SMOG Index	12.7	Complex words	29
Reading Age (years)	20.5	Coleman Liau Index	16.2	Percent of complex words	20.3
Flesch Kincaid Reading Ease	36.5	Automated Readability Index	16.1	Average words per sentence	23.9
Flesch Kincaid Grade Level	14.1	Sentences	6	Average syllables per word	1.7

Lay Summary (extended Prompt)

This study reviews the role of BRCA gene mutations in prostate cancer and how they affect a patient's risk and treatment options. BRCA1 and BRCA2 mutations, often linked to breast and ovarian cancers, can also increase the risk of developing prostate cancer. These mutations are found in a small percentage of prostate cancer patients but are associated with more aggressive forms of the disease and worse outcomes. The study suggests that genetic testing for BRCA mutations should be considered for patients with advanced prostate cancer, a family history of prostate cancer, or those with close relatives who had breast or ovarian cancer at a young age. Understanding these mutations can help doctors better assess a patient's risk and choose the most effective treatment, potentially improving outcomes for patients and their families.

Evaluation of the Lay Summary

**Assessment Category**	**Score**	**Assessment Category**	**Score**	**Assessment Category**	**Score**
Comprehension Score	5	Gunning Fog Score	16.6	Words	131
Accuracy Score	0	SMOG Index	11.6	Complex words	20
Reading Age (years)	20.5	Coleman Liau Index	15.4	Percent of complex words	15.3
Flesch Kincaid Reading Ease	42.7	Automated Readability Index	16.6	Average words per sentence	26.2
Flesch Kincaid Grade Level	13.8	Sentences	5	Average syllables per word	1.6

Manuscript 71

Zara Rozalen A, Martin JM, Rajendran R, Jain M, Nava VE. Ductal Adenocarcinoma of the Prostate with Novel Genetic Alterations Characterized by Next-Generation Sequencing. Curr Oncol. 2024 Mar 19;31(3):1556-1561. doi: 10.3390/curroncol31030118. PMID: 38534951; PMCID: PMC10968787.

Abstract

Ductal adenocarcinoma of the prostate (DAP) is an uncommon variant of prostate cancer associated with aggressive disease and poor outcome. It presents most frequently as a mixed tumor combined with acinar adenocarcinoma. Although the histopathological features of DAP are well known, its genomic characteristics are still evolving, prompting the suggestion that all DAP would benefit from molecular analysis with the purpose of improving tumor recognition, genetic classification, and, ultimately, personalized therapy. Herein, we report a case of DAP with novel genetic alterations (BCOR P1153S, ERG M219I, KDR A750E, POLE S1896P, and RAD21 T461del).

Keywords:

ductal adenocarcinoma; prostate cancer; next-generation sequencing; genetic mutations

Lay Summary (simple Prompt)

This study focuses on ductal adenocarcinoma of the prostate (DAP), a rare and aggressive type of prostate cancer. Unlike more common forms of prostate cancer, DAP has distinct genetic features that are not yet fully understood. The researchers used advanced genetic testing to analyze a case of DAP and discovered several novel genetic mutations. Understanding these unique genetic changes could improve the way DAP is diagnosed and classified, and it may lead to more personalized treatment options for patients. The findings suggest that molecular analysis could be beneficial for all cases of DAP, helping to better identify the cancer and tailor therapies specifically to its genetic profile. This research provides new insights into the genetic basis of this rare cancer and opens the door to more targeted and effective treatments.

Evaluation of the Lay Summary

**Assessment Category**	**Score**	**Assessment Category**	**Score**	**Assessment Category**	**Score**
Comprehension Score	5	Gunning Fog Score	17.0	Words	130
Accuracy Score	0	SMOG Index	12.3	Complex words	27
Reading Age (years)	20.5	Coleman Liau Index	15.7	Percent of complex words	20.8
Flesch Kincaid Reading Ease	35.8	Automated Readability Index	14.6	Average words per sentence	21.7
Flesch Kincaid Grade Level	13.6	Sentences	6	Average syllables per word	1.8

Lay Summary (extended Prompt)

This study focuses on ductal adenocarcinoma of the prostate (DAP), a rare and aggressive form of prostate cancer. The aim was to identify new genetic changes in DAP that could help doctors better understand the disease and develop more targeted treatments. Using advanced genetic testing called next-generation sequencing, the researchers found several new genetic alterations in a patient with DAP. These findings are important because they could lead to improved ways to diagnose and treat this rare cancer. Understanding these genetic changes can help in creating more personalized treatment plans, which could improve outcomes for patients with DAP. This research emphasizes the need for molecular analysis in all cases of DAP to better recognize and treat the disease.

Evaluation of the Lay Summary

**Assessment Category**	**Score**	**Assessment Category**	**Score**	**Assessment Category**	**Score**
Comprehension Score	5	Gunning Fog Score	15.7	Words	119
Accuracy Score	1	SMOG Index	11.3	Complex words	23
Reading Age (years)	19.5	Coleman Liau Index	16.1	Percent of complex words	19.3
Flesch Kincaid Reading Ease	45.2	Automated Readability Index	14.0	Average words per sentence	19.8
Flesch Kincaid Grade Level	11.9	Sentences	6	Average syllables per word	1.7

Manuscript 72

Chi KN, Yip SM, Bauman G, Probst S, Emmenegger U, Kollmannsberger CK, Martineau P, Niazi T, Pouliot F, Rendon R, Hotte SJ, Laidley DT, Saad F. ^{177}Lu-PSMA-617 in Metastatic Castration-Resistant Prostate Cancer: A Review of the Evidence and Implications for Canadian Clinical Practice. Curr Oncol. 2024 Mar 7;31(3):1400-1415. doi: 10.3390/curroncol31030106. PMID: 38534939; PMCID: PMC10969693.

Abstract

Prostate-specific membrane antigen (PSMA) is highly expressed in prostate cancer and a therapeutic target. Lutetium-177 (177Lu)-PSMA-617 is the first radioligand therapy to be approved in Canada for use in patients with metastatic castration-resistant prostate cancer (mCRPC). As this treatment represents a new therapeutic class, guidance regarding how to integrate it into clinical practice is needed. This article aims to review the evidence from prospective phase 2 and 3 clinical trials and meta-analyses of observational studies on the use of 177Lu-PSMA-617 in prostate cancer and discuss how Canadian clinicians might best apply these data in practice. The selection of appropriate patients, the practicalities of treatment administration, including necessary facilities for treatment procedures, the assessment of treatment response, and the management of adverse events are considered. Survival benefits were observed in clinical trials of 177Lu-PSMA-617 in patients with progressive, PSMA-positive mCRPC who were pretreated with androgen receptor pathway inhibitors and taxanes, as well as in taxane-naïve patients. However, the results of ongoing trials are awaited to clarify questions regarding the optimal sequencing of 177Lu-PSMA-617 with other therapies, as well as the implications of predictive biomarkers, personalized dosimetry, and combinations with other therapies.

Keywords:

prostate cancer; mCRPC; radioligand therapy; 177Lu-PSMA-617; 177Lu vipivotide tetraxetan

Lay Summary (simple Prompt)

This study reviews the use of a new treatment called 177Lu-PSMA-617 for men with advanced prostate cancer that has not responded to traditional hormone therapy. The treatment targets a specific protein found on prostate cancer cells and delivers radiation directly to the cancer. The review examines evidence from clinical trials and studies to understand the benefits and challenges of using this treatment in Canada. It discusses how doctors can select the right patients for this therapy, how to administer it safely, and how to manage side effects. The findings show that 177Lu-PSMA-617 can improve survival in patients with advanced prostate cancer. However, more research is needed to determine the best way to combine this treatment with other therapies and to personalize it based on individual patient characteristics. This research could help guide doctors in effectively using this new therapy to improve patient outcomes.

Evaluation of the Lay Summary

**Assessment Category**	**Score**	**Assessment Category**	**Score**	**Assessment Category**	**Score**
Comprehension Score	5	Gunning Fog Score	14.7	Words	147
Accuracy Score	0	SMOG Index	10.7	Complex words	24
Reading Age (years)	18.5	Coleman Liau Index	14.8	Percent of complex words	16.3
Flesch Kincaid Reading Ease	45.7	Automated Readability Index	13.6	Average words per sentence	21.0
Flesch Kincaid Grade Level	12.1	Sentences	7	Average syllables per word	1.7

Lay Summary (extended Prompt)

This study looks at a new treatment called 177Lu-PSMA-617 for prostate cancer that has spread and no longer responds to standard hormone therapy. The treatment uses a targeted radiation approach to attack cancer cells. The review focuses on how this treatment could be used in Canada, discussing who might benefit most, how to give the treatment, and how to handle any side effects. Early clinical trials have shown that 177Lu-PSMA-617 can help patients live longer, especially those whose cancer continues to grow after other treatments. However, more research is needed to understand the best way to combine this new therapy with existing treatments and to identify which patients will benefit most. This research could help doctors make better treatment decisions for men with advanced prostate cancer.

Evaluation of the Lay Summary

**Assessment Category**	**Score**	**Assessment Category**	**Score**	**Assessment Category**	**Score**
Comprehension Score	5	Gunning Fog Score	13.3	Words	130
Accuracy Score	0	SMOG Index	9.5	Complex words	16
Reading Age (years)	17.5	Coleman Liau Index	13.8	Percent of complex words	12.3
Flesch Kincaid Reading Ease	53.4	Automated Readability Index	13.3	Average words per sentence	21.7
Flesch Kincaid Grade Level	11.2	Sentences	6	Average syllables per word	1.6

Manuscript 73

Abufaraj M, Ramadan R, Alkhatib A. Paraneoplastic Syndromes in Neuroendocrine Prostate Cancer: A Systematic Review. Curr Oncol. 2024 Mar 21;31(3):1618-1632. doi: 10.3390/curroncol31030123. PMID: 38534956; PMCID: PMC10969281.

Abstract

Neuroendocrine prostate cancer (NEPC) is a rare subtype of prostate cancer (PCa) that usually results in poor clinical outcomes and may be accompanied by paraneoplastic syndromes (PNS). NEPC is becoming more frequent. It can initially manifest as PNS, complicating diagnosis. Therefore, we reviewed the literature on the different PNS associated with NEPC. We systematically reviewed English-language articles from January 2017 to September 2023, identifying 17 studies meeting PRISMA guidelines for NEPC and associated PNS. A total of 17 articles were included in the review. Among these, Cushing's Syndrome (CS) due to ectopic Adrenocorticotropic hormone (ACTH) secretion was the most commonly reported PNS. Other PNS included syndrome of inappropriate Anti-Diuretic Hormone secretion (SIADH), Anti-Hu-mediated chronic intestinal pseudo-obstruction (CIPO), limbic encephalitis, Evans Syndrome, hypercalcemia, dermatomyositis, and polycythemia. Many patients had a history of prostate adenocarcinoma treated with androgen deprivation therapy (ADT) before neuroendocrine features developed. The mean age was 65.5 years, with a maximum survival of 9 months post-diagnosis. NEPC is becoming an increasingly more common subtype of PCa that can result in various PNS. This makes the diagnosis and treatment of NEPC challenging. Further research is crucial to understanding these syndromes and developing standardized, targeted treatments to improve patient survival.

Keywords:

prostate cancer; paraneoplastic; neuroendocrine tumor

Lay Summary (simple Prompt)

Neuroendocrine prostate cancer (NEPC) is a rare and aggressive form of prostate cancer that often leads to poor outcomes. It can be difficult to diagnose because it may cause paraneoplastic syndromes (PNS), which are rare conditions caused by cancer. This review looked at 17 studies published between 2017 and 2023 to explore the link between NEPC and PNS. The most common syndrome found was Cushing's Syndrome, caused by abnormal hormone production. Other PNS included conditions like SIADH, limbic encephalitis, and hypercalcemia. Many patients developed NEPC after being treated for prostate adenocarcinoma with hormone therapy. Most patients were around 65 years old, with a survival rate of up to nine months after diagnosis. Because NEPC and its related syndromes complicate diagnosis and treatment, more research is needed to find better ways to treat and improve the survival of these patients.

Evaluation of the Lay Summary

**Assessment Category**	**Score**	**Assessment Category**	**Score**	**Assessment Category**	**Score**
Comprehension Score	5	Gunning Fog Score	12.7	Words	139
Accuracy Score	0	SMOG Index	9.4	Complex words	21
Reading Age (years)	17.5	Coleman Liau Index	15.3	Percent of complex words	15.1
Flesch Kincaid Reading Ease	46.2	Automated Readability Index	12.1	Average words per sentence	17.4
Flesch Kincaid Grade Level	11.1	Sentences	8	Average syllables per word	1.7

Lay Summary (extended Prompt)

This study focuses on a rare type of prostate cancer called neuroendocrine prostate cancer (NEPC), which often leads to severe health problems and is becoming more common. NEPC can cause unusual symptoms known as paraneoplastic syndromes (PNS), which make diagnosis more difficult. The researchers reviewed recent studies to understand the different PNS associated with NEPC. They found that the most common PNS is Cushing's Syndrome, but other symptoms like abnormal hormone levels and autoimmune reactions were also reported. Many patients had previously been treated for a different type of prostate cancer before developing NEPC. On average, patients with NEPC lived about nine months after diagnosis. This study highlights the need for more research to better understand NEPC and its symptoms, aiming to improve diagnosis, treatment, and survival rates for patients with this challenging condition.

Evaluation of the Lay Summary

**Assessment Category**	**Score**	**Assessment Category**	**Score**	**Assessment Category**	**Score**
Comprehension Score	4	Gunning Fog Score	14.2	Words	134
Accuracy Score	0	SMOG Index	10.3	Complex words	22
Reading Age (years)	19.5	Coleman Liau Index	17.2	Percent of complex words	16.4
Flesch Kincaid Reading Ease	40.9	Automated Readability Index	14.5	Average words per sentence	19.1
Flesch Kincaid Grade Level	12.3	Sentences	7	Average syllables per word	1.7

Manuscript 74

MacNevin W, Ilie G, Rendon R, Mason R, Spooner J, Chedrawe E, Patil N, Bowes D, Bailly G, Bell D, Wilke D, Zahavich JBL, MacDonald C, Rutledge RDH. PC-PEP, a Comprehensive Daily Six-Month Home-Based Patient Empowerment Program Leads to Weight Loss in Men with Prostate Cancer: A Secondary Analysis of a Clinical Trial. Curr Oncol. 2024 Mar 21;31(3):1667-1688. doi: 10.3390/curroncol31030127. PMID: 38534960; PMCID: PMC10969418.

Abstract

Background: The Prostate Cancer—Patient Empowerment Program (PC-PEP) is a six-month daily home-based program shown to improve mental health and urinary function. This secondary analysis explores weight loss in male PC-PEP participants. Methods: In a randomized clinical trial with 128 men undergoing curative prostate cancer (PC) treatment, 66 received 'early' PC-PEP, while 62 were assigned to the 'late' waitlist-control group, receiving 6 months of standard-of-care treatment followed by 6 months of PC-PEP. PC-PEP comprised 182 daily emails with video-based exercise and dietary (predominantly plant-based) education, live online events, and 30 min strength training routines (using body weight and elastic bands). Weight and height data were collected via online surveys (baseline, 6 months, and 12 months) including medical chart reviews. Adherence was tracked weekly. Results: No attrition or adverse events were reported. At 6 months, the early PC-PEP group experienced significant weight loss, averaging 2.7 kg ($p < 0.001$) compared to the waitlist-control group. Weight loss was noted in the late intervention group of PC-PEP, albeit less pronounced than in the early group. Early PC-PEP surgery patients lost on average 1.4 kg (SE = 0.65) from the trial's start to surgery day. High adherence to exercise and dietary recommendations was noted. Conclusions: PC-PEP led to significant weight loss in men undergoing curative prostate cancer treatment compared to standard-of-care.
prostate cancer; curative treatment; exercise intervention; radical prostatectomy; radiation; weight loss; Body Mass Index; physical fitness; behavioural intervention; weight management

Keywords:

Body Mass Index; behavioural intervention; curative treatment; exercise intervention; physical fitness; prostate cancer; radiation; radical prostatectomy; weight loss; weight management

Lay Summary (simple Prompt)

This study examines the effects of a six-month home-based program called the Prostate Cancer—Patient Empowerment Program (PC-PEP) on weight loss in men undergoing prostate cancer treatment. The program includes daily exercise routines, dietary guidance with a focus on plant-based nutrition, and educational activities delivered through videos and online events. In a clinical trial involving 128 men, those who started the PC-PEP program early experienced significant weight loss, averaging 2.7 kg over six months, compared to a control group receiving standard care. Men in the control group also lost weight when they eventually started the program, though the effect was less pronounced. The study found high adherence to the program's exercise and dietary recommendations, with no dropouts or reported adverse effects. These results suggest that PC-PEP is an effective approach for promoting weight loss and overall health in men undergoing prostate cancer treatment

Evaluation of the Lay Summary

**Assessment Category**	**Score**	**Assessment Category**	**Score**	**Assessment Category**	**Score**
Comprehension Score	5	Gunning Fog Score	14.7	Words	149
Accuracy Score	0	SMOG Index	11.0	Complex words	25
Reading Age (years)	19.5	Coleman Liau Index	15.9	Percent of complex words	16.8
Flesch Kincaid Reading Ease	41.0	Automated Readability Index	14.6	Average words per sentence	21.3
Flesch Kincaid Grade Level	12.8	Sentences	7	Average syllables per word	1.7

Lay Summary (extended Prompt)

This study looked at how a home-based program called PC-PEP, designed for men with prostate cancer, can help with weight loss. The program lasted six months and included daily emails with exercise routines and healthy eating tips, mostly focused on plant-based diets. In a clinical trial, 128 men undergoing prostate cancer treatment were divided into two groups. One group started the program right away, while the other group began six months later. The study found that men who started the PC-PEP program right away lost more weight (an average of 2.7 kg) than those who waited. The program also encouraged participants to stay active and eat better, leading to further weight loss. These results suggest that the PC-PEP program can be an effective way to help men with prostate cancer lose weight and improve their overall health while undergoing treatment.

Evaluation of the Lay Summary

**Assessment Category**	**Score**	**Assessment Category**	**Score**	**Assessment Category**	**Score**
Comprehension Score	5	Gunning Fog Score	9.8	Words	146
Accuracy Score	0	SMOG Index	6.3	Complex words	9
Reading Age (years)	14.5	Coleman Liau Index	12.3	Percent of complex words	6.2
Flesch Kincaid Reading Ease	65.5	Automated Readability Index	10.1	Average words per sentence	18.3
Flesch Kincaid Grade Level	8.7	Sentences	8	Average syllables per word	1.5

Manuscript 75

Oyogoa E, Sonpatki M, Brinkerhoff BT, Andeen N, Meyer H, Ryan C, Sokolova AO. Mixed Adenosquamous Cell Carcinoma of the Prostate with Paired Sequencing on the Primary and Liver Metastasis. Curr Oncol. 2024 Apr 24;31(5):2393-2399. doi: 10.3390/curroncol31050178. PMID: 38785459; PMCID: PMC11119923.

Abstract

This report aims to shed light on the intricate challenges encountered during the diagnosis and treatment of an uncommon variant of prostate cancer—mixed adenosquamous cell carcinoma of the prostate. Prostate cancers of this nature pose distinctive diagnostic and therapeutic dilemmas due to their rarity and complex histological composition. We present a case of a 63-year-old man with metastatic prostate cancer, featuring adenocarcinoma with squamous cell differentiation, who underwent a multimodal treatment approach. The patient responded to first-line carboplatin, docetaxel, and androgen deprivation therapy, followed by androgen receptor pathway inhibitor (ARPI) maintenance. However, disease progression led to radiation therapy and a subsequent switch to Lutetium (177Lu) vipivotide tetraxetan after chemotherapy challenges. Comprehensive genetic profiling revealed shared mutations in the prostate and liver lesions, emphasizing the role of targeted therapies. Prostate-specific membrane antigen (PSMA)-targeted therapy resulted in a notable PSA decline. This case highlights the evolving treatment landscape for rare prostate cancers, integrating genetic insights for tailored interventions. In conclusion, squamous cell carcinoma (SCC) of the prostate is rare, emphasizing the imperative for enhanced comprehension in diagnosis and management. Our case suggests the potential efficacy of ARPI and PSMA-targeted therapies. Our findings advocate for a more nuanced approach to the management of this rare prostate cancer variant, leveraging genomic insights for personalized treatment strategies. This exploration serves as a foundation for further research and clinical considerations in addressing the challenges posed by mixed adenosquamous cell carcinoma of the prostate.

Keywords:

squamous cell carcinoma of the prostate; mixed adenosquamous cell carcinoma of the prostate; prostate cancer; prostate-specific membrane antigen positron emission tomography (PSMA PET)

Lay Summary (simple Prompt)

This study focuses on a rare and complex type of prostate cancer called mixed adenosquamous cell carcinoma, which combines features of two different cancer types, making it difficult to diagnose and treat. The report discusses the case of a 63-year-old man with this rare cancer that had spread to his liver. The patient received a combination of treatments, including chemotherapy, hormone therapy, radiation, and a novel targeted therapy called PSMA-targeted therapy. Genetic analysis revealed common mutations in both the prostate and liver cancer, suggesting that personalized treatment based on genetic profiling could be effective. The patient responded well to the treatment, with a significant decrease in PSA levels, a marker of prostate cancer. This case highlights the importance of using genetic information to guide treatment decisions and suggests new potential strategies for managing rare forms of prostate cancer more effectively.

Evaluation of the Lay Summary

**Assessment Category**	**Score**	**Assessment Category**	**Score**	**Assessment Category**	**Score**
Comprehension Score	5	Gunning Fog Score	18.5	Words	143
Accuracy Score	0	SMOG Index	13.3	Complex words	32
Reading Age (years)	21.5	Coleman Liau Index	16.6	Percent of complex words	22.4
Flesch Kincaid Reading Ease	32.4	Automated Readability Index	16.4	Average words per sentence	23.9
Flesch Kincaid Grade Level	14.7	Sentences	6	Average syllables per word	1.8

Lay Summary (extended Prompt)

This study focuses on a rare type of prostate cancer called mixed adenosquamous cell carcinoma, which is difficult to diagnose and treat due to its unique combination of cancer cells. We present the case of a 63-year-old man with this rare cancer type, who was treated with multiple therapies, including chemotherapy, radiation, and hormone treatments. When his cancer progressed, he was given a new type of therapy targeting a protein specific to prostate cancer cells, which led to a significant drop in his PSA levels, an indicator of cancer activity. Genetic tests showed the same mutations in both the primary and metastatic cancer sites, suggesting targeted therapies could be effective. This case highlights the importance of personalized treatment plans based on genetic information for better outcomes in rare prostate cancers and supports further research into tailored therapies for such challenging cases.

Evaluation of the Lay Summary

Assessment Category	Score	Assessment Category	Score	Assessment Category	Score
Comprehension Score	5	Gunning Fog Score	20.1	Words	143
Accuracy Score	0	SMOG Index	14.3	Complex words	31
Reading Age (years)	22.5	Coleman Liau Index	15.5	Percent of complex words	21.7
Flesch Kincaid Reading Ease	31.1	Automated Readability Index	17.9	Average words per sentence	28.6
Flesch Kincaid Grade Level	16.0	Sentences	5	Average syllables per word	1.7

Manuscript 76

Potkrajcic V, Gani C, Fischer SG, Boeke S, Niyazi M, Thorwarth D, Voigt O, Schneider M, Mönnich D, Kübler S, Boldt J, Hoffmann E, Paulsen F, Mueller AC, Wegener D. Online Adaptive MR-Guided Ultrahypofractionated Radiotherapy of Prostate Cancer on a 1.5 T MR-Linac: Clinical Experience and Prospective Evaluation. Curr Oncol. 2024 May 9;31(5):2679-2688. doi: 10.3390/curroncol31050203. PMID: 38785484; PMCID: PMC11120184.

Abstract

The use of hypofractionated radiotherapy in prostate cancer has been increasingly evaluated, whereas accumulated evidence demonstrates comparable oncologic outcomes and toxicity rates compared to normofractionated radiotherapy. In this prospective study, we evaluate all patients with intermediate-risk prostate cancer treated with ultrahypofractionated (UHF) MRI-guided radiotherapy on a 1.5 T MR-Linac within our department and report on workflow and feasibility, as well as physician-recorded and patient-reported longitudinal toxicity. A total of 23 patients with intermediate-risk prostate cancer treated on the 1.5 T MR-Linac with a dose of 42.7 Gy in seven fractions (seven MV step-and-shoot IMRT) were evaluated within the MRL-01 study (NCT04172753). The duration of each treatment step, choice of workflow (adapt to shape-ATS or adapt to position-ATP) and technical and/or patient-sided treatment failure were recorded for each fraction and patient. Acute and late toxicity were scored according to RTOG and CTC V4.0, as well as the use of patient-reported questionnaires. The median follow-up was 12.4 months. All patients completed the planned treatment. The mean duration of a treatment session was 38.2 min. In total, 165 radiotherapy fractions were delivered. ATS was performed in 150 fractions, 5 fractions were delivered using ATP, and 10 fractions were delivered using both ATS and ATP workflows. Severe acute bother (G3+) regarding IPS-score was reported in five patients (23%) at the end of radiotherapy. However, this tended to normalize and no G3+ IPS-score was observed later at any point during follow-up. Furthermore, no other severe genitourinary (GU) or gastrointestinal (GI) acute or late toxicity was observed. One-year biochemical-free recurrence survival was 100%. We report the excellent feasibility of UHF MR-guided radiotherapy for intermediate-risk prostate cancer patients and acceptable toxicity rates

in our preliminary study. Randomized controlled studies with long-term follow-up are warranted to detect possible advantages over current state-of-the-art RT techniques.

Keywords:
prostate cancer; hypofractionated radiotherapy; MR-Linac

Lay Summary (simple Prompt)

This study examines the use of a new radiotherapy method, called ultrahypofractionated MRI-guided radiotherapy, for treating prostate cancer. This approach delivers higher doses of radiation over fewer sessions, potentially making treatment shorter and more convenient. Researchers treated 23 patients with intermediate-risk prostate cancer using a 1.5 Tesla MR-Linac machine and monitored them for over a year. They measured treatment times, side effects, and effectiveness. The study found that all patients completed the planned treatments with manageable side effects, which mostly improved over time. No severe long-term urinary or gastrointestinal problems were observed, and all patients were free from cancer recurrence one year after treatment. These promising results suggest that this new radiotherapy method is safe and effective for treating prostate cancer, but more research with larger groups and longer follow-ups is needed to confirm its benefits over existing treatments.

Evaluation of the Lay Summary

Assessment Category	Score	Assessment Category	Score	Assessment Category	Score
Comprehension Score	5	Gunning Fog Score	13.3	Words	145
Accuracy Score	0	SMOG Index	9.7	Complex words	22
Reading Age (years)	19.5	Coleman Liau Index	17.8	Percent of complex words	15.2
Flesch Kincaid Reading Ease	33.8	Automated Readability Index	14.5	Average words per sentence	18.1
Flesch Kincaid Grade Level	13.0	Sentences	8	Average syllables per word	1.8

Lay Summary (extended Prompt)

This study looked at a new way to treat prostate cancer using a special type of radiation therapy called ultrahypofractionated radiotherapy, guided by MRI. This method uses fewer sessions with higher doses of radiation each time. Researchers treated 23 men with intermediate-risk prostate cancer and closely monitored them for side effects and treatment success. The study found that this treatment was safe and effective, with all patients completing their therapy and no severe long-term side effects reported. Only a few patients experienced temporary discomfort, which improved over time. After one year, none of the patients had signs of cancer coming back. These early results suggest that MRI-guided ultrahypofractionated radiotherapy could be a good option for treating prostate cancer, but more research with longer follow-up is needed to see if it is better than current methods.

Evaluation of the Lay Summary

**Assessment Category**	**Score**	**Assessment Category**	**Score**	**Assessment Category**	**Score**
Comprehension Score	5	Gunning Fog Score	12.5	Words	139
Accuracy Score	0	SMOG Index	8.8	Complex words	16
Reading Age (years)	18.5	Coleman Liau Index	15.4	Percent of complex words	11.5
Flesch Kincaid Reading Ease	41.8	Automated Readability Index	13.5	Average words per sentence	19.9
Flesch Kincaid Grade Level	12.4	Sentences	7	Average syllables per word	0

Manuscript 77

Tomioka M, Nakane K, Kawase M, Iinuma K, Kato D, Kawase K, Taniguchi T, Tobisawa Y, Sugino F, Kaga T, Kato H, Matsuo M, Kito Y, Saigo C, Suzui N, Ito T, Miyazaki T, Takeuchi T, Koie T. Discrepancy in the Location of Prostate Cancer Indicated on Biparametric Magnetic Resonance Imaging and Pathologically Diagnosed Using Surgical Specimens. Curr Oncol. 2024 May 16;31(5):2846-2855. doi: 10.3390/curroncol31050216. PMID: 38785497; PMCID: PMC11119495.

Abstract

Accurate diagnosis of the localization of prostate cancer (PCa) on magnetic resonance imaging (MRI) remains a challenge. We aimed to assess discrepancy between the location of PCa pathologically diagnosed using surgical specimens and lesions indicated as possible PCa by the Prostate Imaging Reporting and Data System on MRI. The primary endpoint was the concordance rate between the site of probable clinically significant PCa (csPCa) identified using biparametric MRI (bpMRI) and location of PCa in the surgical specimen obtained using robot-assisted total prostatectomy. Among 85 lesions identified in 30 patients; 42 (49.4%) were identified as possible PCa on MRI. The 85 PCa lesions were divided into positive and negative groups based on the bpMRI results. None of the patients had missed csPCa. Although the diagnostic accuracy of bpMRI was relatively high for PCas located in the middle of the prostate ($p = 0.029$), it was relatively low for PCa located at the base of the prostate, all of which were csPCas. Although current modalities can accurately diagnose PCa, the possibility that PCa is present with multiple lesions in the prostate should be considered, even if MRI does not detect PCa.

Keywords:

prostate cancer; biparametric magnetic resonance imaging; prostate biopsy; tumor location; diagnostic discrepancy

Lay Summary (simple Prompt)

This study investigates how accurately magnetic resonance imaging (MRI) can pinpoint the exact location of prostate cancer within the prostate gland. Researchers compared the results of MRI scans, which are used to identify potential cancer areas, with the actual location of cancer found in surgical specimens from patients who underwent prostate removal surgery. The findings showed that while MRI is fairly accurate in detecting cancers in the middle of the prostate, it is less reliable in identifying cancers at the base of the prostate. These results highlight that prostate cancer can exist in multiple areas, even if MRI does not detect it in all locations. This research underscores the need for improved imaging techniques to better identify all cancer areas, which could lead to more accurate diagnoses and better treatment planning for prostate cancer patients.

Evaluation of the Lay Summary

**Assessment Category**	**Score**	**Assessment Category**	**Score**	**Assessment Category**	**Score**
Comprehension Score	5	Gunning Fog Score	19.1	Words	135
Accuracy Score	0	SMOG Index	13.6	Complex words	28
Reading Age (years)	21.5	Coleman Liau Index	15.9	Percent of complex words	20.7
Flesch Kincaid Reading Ease	30.3	Automated Readability Index	13.4	Average words per sentence	27.0
Flesch Kincaid Grade Level	15.7	Sentences	5	Average syllables per word	1.8

Lay Summary (extended Prompt)

This study looked at how well a type of MRI called biparametric MRI (bpMRI) can find the exact location of prostate cancer compared to what is found in actual surgical samples. The researchers wanted to see how often the MRI results matched the cancer locations found after surgery. They found that the MRI was good at detecting cancer in the middle of the prostate but was less accurate for cancers at the base of the prostate. Even when the MRI did not show cancer, the study suggests that prostate cancer might still be present in other areas. This finding is important because it means doctors should consider checking for cancer in multiple areas of the prostate, even if the MRI does not show it, to avoid missing any significant tumors.

Evaluation of the Lay Summary

Assessment Category	Score	Assessment Category	Score	Assessment Category	Score
Comprehension Score	5	Gunning Fog Score	14.4	Words	130
Accuracy Score	0	SMOG Index	9.4	Complex words	13
Reading Age (years)	17.5	Coleman Liau Index	11.6	Percent of complex words	10.0
Flesch Kincaid Reading Ease	52.9	Automated Readability Index	13.5	Average words per sentence	26.0
Flesch Kincaid Grade Level	12.3	Sentences	5	Average syllables per word	1.5

Manuscript 78

Sobhani S, Dadabhoy A, Ghoreifi A, Lebastchi AH. Salvage High-Intensity Focused Ultrasound for Prostate Cancer after Radiation Failure: A Narrative Review. Curr Oncol. 2024 Jun 26;31(7):3669-3681. doi: 10.3390/curroncol31070270. PMID: 39057142; PMCID: PMC11276396.

Abstract

For patients diagnosed with localized prostate cancer, there are multiple treatment options available. The traditional treatment modalities include radical prostatectomy and radiotherapy. Nevertheless, focal therapy, including high-intensity focused ultrasound (HIFU) and cryotherapy, has emerged as a less-invasive method in this setting. Some patients undergoing primary radiation therapy experience recurrence, but there is currently no consensus on the optimal approach for salvage treatment in such cases. The lack of robust data and randomized controlled trials comparing different whole-gland and focal salvage therapies presents a challenge in determining the ideal treatment strategy. This narrative review examines the prospective and retrospective data available on salvage HIFU following radiation therapy. Based on the literature, salvage HIFU for radio-recurrent prostate cancer has promising oncological outcomes, with an overall 5-year survival rate of around 85%, as well as incontinence rates of about 30% based on the patient's risk group, follow-up times, definitions used, and other aspects of the study. Salvage HIFU for prostate cancer proves to be an effective treatment modality for select patients with biochemical recurrence following radiotherapy.

Keywords:

(MeSH) salvage therapy; high-intensity focused ultrasound; prostate cancer; cancer recurrence; radiotherapy; minimally invasive surgical procedures

Lay Summary (simple Prompt)

This study reviews the use of high-intensity focused ultrasound (HIFU) as a treatment for prostate cancer that has returned after initial radiation therapy. When prostate cancer is first diagnosed, treatments like surgery or radiation are commonly used, but some patients may experience cancer recurrence after radiation. Currently, there is no clear agreement on the best treatment for these cases. This review looks at existing research on using HIFU, a less invasive treatment option, to target recurring cancer. The findings suggest that HIFU offers promising outcomes, with about 85% of patients surviving at least five years after treatment. However, around 30% of patients may experience incontinence, depending on various factors. The results indicate that HIFU could be an effective treatment for certain patients with recurring prostate cancer after radiation, offering a potential alternative to more invasive procedures.

Evaluation of the Lay Summary

**Assessment Category**	**Score**	**Assessment Category**	**Score**	**Assessment Category**	**Score**
Comprehension Score	5	Gunning Fog Score	17.5	Words	137
Accuracy Score	0	SMOG Index	12.5	Complex words	33
Reading Age (years)	20.5	Coleman Liau Index	17.4	Percent of complex words	24.1
Flesch Kincaid Reading Ease	30.7	Automated Readability Index	14.9	Average words per sentence	19.6
Flesch Kincaid Grade Level	13.8	Sentences	7	Average syllables per word	1.9

Lay Summary (extended Prompt)

This study reviews the use of high-intensity focused ultrasound (HIFU) as a treatment option for prostate cancer that comes back after initial radiation therapy. When prostate cancer is first found, it can be treated with surgery, radiation, or less invasive methods like HIFU. However, if cancer returns after radiation, there is no clear agreement on the best treatment approach. This review looked at various studies on using HIFU in these cases and found that it has good results, with about 85% of patients surviving for at least five years. HIFU also has a moderate risk of side effects, such as incontinence, in about 30% of patients. These findings suggest that HIFU could be an effective option for some patients whose prostate cancer returns after radiation.

Evaluation of the Lay Summary

**Assessment Category**	**Score**	**Assessment Category**	**Score**	**Assessment Category**	**Score**
Comprehension Score	5	Gunning Fog Score	13.8	Words	126
Accuracy Score	0	SMOG Index	9.8	Complex words	17
Reading Age (years)	17.5	Coleman Liau Index	13.2	Percent of complex words	13.5
Flesch Kincaid Reading Ease	47.2	Automated Readability Index	12.3	Average words per sentence	21.0
Flesch Kincaid Grade Level	11.9	Sentences	6	Average syllables per word	1.6

Manuscript 79

Yoo GS, Park S, Rim CH, Cho WK, Chang AR, Kim YS, Ahn YC, Chie EK; Oligometastasis Working Group, Korean Cancer Association. Radiation Oncologists' Perspectives on Oligometastatic Prostate Cancer: A Survey from Korean Oligometastasis Working Group. Curr Oncol. 2024 Jun 3;31(6):3239-3251. doi: 10.3390/curroncol31060245. PMID: 38920729; PMCID: PMC11203304.

Abstract

Background: Interest in the oligometastatic prostate cancer (OMPC) is increasing, and various clinical studies have reported the benefits of metastasis-directed radiation therapy (MDRT) in OMPC. However, the recognition regarding the adopted definitions, methodologies of assessment, and therapeutic approaches is diverse among radiation oncologists. This study aims to evaluate the level of agreement for issues in OMPC among radiation oncologists. Methods: We generated 15 key questions (KQs) for OMPC relevant to definition, diagnosis, local therapies, and endpoints. Additionally, three clinical scenarios representing synchronous metastatic prostate cancer (mPC) (case 1), metachronous mPC with visceral metastasis (case 2), and metachronous mPC with castration-resistance and history of polymetastasis (case 3) were developed. The 15 KQs were adapted according to each scenario and transformed into 23 questions with 6–9 per scenario. The survey was distributed to 80 radiation oncologists throughout the Republic of Korea. Answer options with 0.0–29.9%, 30–49.9%, 50–69.9%, 70–79.9%, 80–89.9%, and 90–100% agreements were considered as no, minimal, weak, moderate, strong, and near perfect agreement, respectively. Results: Forty-five candidates voluntarily participated in this study. Among 23 questions, near perfect (n = 4), strong (n = 3), or moderate (n = 2) agreements were shown in nine. For the case recognized as OMPC with agreements of 93% (case 1), near perfect agreements on the application of definitive radiation therapy (RT) for whole metastatic lesions were achieved. While ≥70% agreements regarding optimal dose-fractionation for metastasis-directed RT (MDRT) has not been achieved, stereotactic body RT (SBRT) is favored by clinicians with higher clinical volume. Conclusion: For the case recognized as OMPC, near perfect agreement for the application of definitive RT for whole metastatic lesions was reached. SBRT was more favored as a MDRT by clinicians with a higher clinical volume.

Keywords:

local therapy; oligometastasis; prostate cancer; radiation therapy; survey study

Lay Summary (simple Prompt)

This study looked at the opinions of radiation oncologists in South Korea on treating prostate cancer that has spread to a few other parts of the body (called oligometastatic prostate cancer). The researchers sent a survey to 80 oncologists to see if they agreed on how to define, diagnose, and treat this type of cancer. They also presented three patient cases to the doctors, each representing a different stage of the disease. Out of 23 survey questions, the oncologists strongly agreed on nine. One key finding was that most doctors (93%) agreed that radiation should be used to treat all cancer spots in patients with oligometastatic prostate cancer. However, there was less agreement on the best radiation doses to use. Doctors who treated more patients tended to prefer a specific type of radiation called stereotactic body radiation therapy (SBRT).

Evaluation of the Lay Summary

**Assessment Category**	**Score**	**Assessment Category**	**Score**	**Assessment Category**	**Score**
Comprehension Score	5	Gunning Fog Score	14.0	Words	139
Accuracy Score	0	SMOG Index	10.1	Complex words	21
Reading Age (years)	17.5	Coleman Liau Index	13.6	Percent of complex words	15.1
Flesch Kincaid Reading Ease	47.3	Automated Readability Index	12.0	Average words per sentence	19.9
Flesch Kincaid Grade Level	11.6	Sentences	7	Average syllables per word	1.7

Lay Summary (extended Prompt)

This study looked at how doctors in Korea view the treatment of a type of prostate cancer that has spread to only a few areas, known as oligometastatic prostate cancer (OMPC). The researchers wanted to see how much these doctors agree on how to define, diagnose, and treat OMPC. They sent out a survey with questions to 80 radiation doctors, and 45 of them responded. The survey showed that most doctors strongly agree that using radiation therapy on all cancer spots is the best approach for treating OMPC. However, there was less agreement on the exact doses of radiation to use. A specific type of radiation therapy called stereotactic body radiation therapy (SBRT) was preferred by doctors who see more patients. This study helps doctors better understand the best ways to treat OMPC, which could improve patient care.

Evaluation of the Lay Summary

**Assessment Category**	**Score**	**Assessment Category**	**Score**	**Assessment Category**	**Score**
Comprehension Score	5	Gunning Fog Score	12.5	Words	138
Accuracy Score	0	SMOG Index	8.8	Complex words	16
Reading Age (years)	16.5	Coleman Liau Index	12.5	Percent of complex words	11.6
Flesch Kincaid Reading Ease	54.4	Automated Readability Index	11.1	Average words per sentence	19.7
Flesch Kincaid Grade Level	10.6	Sentences	7	Average syllables per word	1.6

Manuscript 80

Ba DM, Kayembe C, Littlejohn J, Van Scoy LJ, VanDyke E, Williams J, Katoch A, Shook NC, Zhang Y, Livelsberger C, McDonald AC, Muscat JE. Navigation-Based Telehealth Informed Decision-Making for Prostate Cancer Screening in Black Men. Curr Oncol. 2024 Jun 28;31(7):3698-3712. doi: 10.3390/curroncol31070273. PMID: 39057145; PMCID: PMC11275421.

Abstract

The rapid increase in telehealth has the potential to bring informed decision-making for prostate cancer screening (PCS) at the population level to high-risk individuals. We utilized a global technology platform of electronic health records data repositories (TriNetX) to determine its utility for Navigator-guided decision-making aid for PCS in Black men ages 45–79 years with no history of prostate cancer and PSA testing. Patients from Pennsylvania were invited to participate in a telehealth-delivered informed decision-making session for PCS. Focus groups, social learning theory, visual diagrams, and quantitative data on PCS risks and benefits were used to develop the content of the sessions, which included numerical discussions of risks vs. benefits in Black men. Participants completed several surveys, including baseline demographic and numeracy questionnaires, a one-on-one telehealth session with a trained Navigator, post-Navigation surveys, and an optional follow-up session with a urologist. Eighty-seven participants were consented and recruited. Although the mean numeracy score was only 1.9 out of 6, more than 90% rated as good or excellent that the sessions aided their PCS decision-making skills. This study indicates that Navigation by telehealth offers the ability to assist in informed decision-making for PCS at the population level.

Keywords:

prostate; cancer; screening; telehealth; telemedicine; navigation; blacks; men; numeracy

Lay Summary (simple Prompt)

Prostate cancer is a serious health concern, especially for Black men, who are at higher risk. This study explores how telehealth can help Black men make informed decisions about prostate cancer screening. Using a global platform of electronic health records, researchers invited Black men in Pennsylvania, aged 45-79, with no history of prostate cancer, to participate in a telehealth session. These sessions, led by trained Navigators, used visual aids and discussions about the risks and benefits of screening. Despite participants having low scores in understanding numbers, over 90% felt that the sessions were good or excellent in helping them make informed decisions about screening. This research shows that telehealth can be an effective way to help high-risk groups, like Black men, understand and decide on prostate cancer screening, potentially improving early detection and treatment.

Evaluation of the Lay Summary

**Assessment Category**	**Score**	**Assessment Category**	**Score**	**Assessment Category**	**Score**
Comprehension Score	5	Gunning Fog Score	15.8	Words	136
Accuracy Score	0	SMOG Index	11.8	Complex words	25
Reading Age (years)	20.5	Coleman Liau Index	16.1	Percent of complex words	18.4
Flesch Kincaid Reading Ease	39.5	Automated Readability Index	15.4	Average words per sentence	22.7
Flesch Kincaid Grade Level	13.4	Sentences	6	Average syllables per word	1.7

Lay Summary (extended Prompt)

Prostate cancer is a serious risk for Black men, and making informed decisions about screening is important. This study looked at how telehealth, which is healthcare done over the phone or computer, can help Black men decide whether to get screened for prostate cancer. Researchers used a big database of health records to invite Black men from Pennsylvania to take part in online sessions. These sessions were led by trained Navigators who used pictures and simple explanations to talk about the risks and benefits of screening. Even though many participants had trouble understanding numbers, over 90% said the sessions were very helpful for making decisions. This study shows that telehealth can be a good way to help Black men learn about and decide on prostate cancer screening, which could lead to better health outcomes.

Evaluation of the Lay Summary

**Assessment Category**	**Score**	**Assessment Category**	**Score**	**Assessment Category**	**Score**
Comprehension Score	5	Gunning Fog Score	12.8	Words	134
Accuracy Score	0	SMOG Index	9.2	Complex words	15
Reading Age (years)	17.5	Coleman Liau Index	14.1	Percent of complex words	11.2
Flesch Kincaid Reading Ease	52.8	Automated Readability Index	13.6	Average words per sentence	22.3
Flesch Kincaid Grade Level	11.4	Sentences	6	Average syllables per word	1.6
